# Manipulating Estrogenic/Anti-Estrogenic Activity of Triphenylethylenes towards Development of Novel Anti-Neoplastic SERMs

**DOI:** 10.3390/ijms222212575

**Published:** 2021-11-22

**Authors:** Heba E. Elnakib, Marian M. Ramsis, Nouran O. Albably, Merna A. Vector, Jan J. Weigand, Kai Schwedtmann, Jannette Wober, Oliver Zierau, Günter Vollmer, Ashraf H. Abadi, Nermin S. Ahmed

**Affiliations:** 1 Department of Pharmaceutical Chemistry, Faculty of Pharmacy and Biotechnology, German University in Cairo, Cairo 11835, Egypt; heba.elnakib@guc.edu.eg (H.E.E.); marian.ramsis@guc.edu.eg (M.M.R.); nouran.albably@guc.edu.eg (N.O.A.); mirna.ayad@guc.edu.eg (M.A.V.); ashraf.abadi@guc.edu.eg (A.H.A.); 2 Faculty of Chemistry and Food Chemistry, Institute of Inorganic Molecular Chemistry, Technische Universität Dresden, 01062 Dresden, Germany; jan.weigand@tu-dresden.de (J.J.W.); kai.schwedtmann@tu-dresden.de (K.S.); 3 Faculty of Biology, Institute of Zoology, Technische Universität Dresden, 01062 Dresden, Germany; oliver.zierau@tu-dresden.de (O.Z.); guenter.vollmer@tu-dresden.de (G.V.)

**Keywords:** tamoxifen, CYP2D6, MCF-7, Ishikawa cells, SERM, TNBC, uterotrophic

## Abstract

Selective estrogen receptor modulators (SERMs) act as estrogen receptor (ERα) agonists or antagonists depending on the target issue. Tamoxifen (TAM) (a non-steroidal triphenylethylene derivative) was the first SERM approved as anti-estrogen for the treatment of metastatic breast cancer. On the hunt for novel SERMs with potential growth inhibitory activity on breast cancer cell lines yet no potential to induce endometrial carcinoma, we designed and synthesized 28 novel TAM analogs. The novel analogs bear a triphenylethylene scaffold. Modifications on rings **A**, **B,** and **C** aim to attenuate estrogenic/anti-estrogenic activities of the novel compounds so they can potentially inhibit breast cancer and provide positive, beneficial estrogenic effects on other tissues with no risk of developing endometrial hyperplasia. Compound **12** (*E/Z*-1-(2-{4-[1-(4-Chloro-phenyl)-2-(4-methoxy-phenyl)-propenyl]-phenoxy}-ethyl)-piperidine) showed an appreciable relative ERα agonistic activity in a yeast estrogen screen (YES) assay. It successfully inhibited the growth of the MCF-7 cell line with GI_50_ = 0.6 µM, and it was approximately three times more potent than TAM. It showed no potential estrogenicity on Ishikawa endometrial adenocarcinoma cell line via assaying alkaline phosphatase (AlkP) activity. Compound **12** was tested in vivo to assess its estrogenic properties in an uterotrophic assay in an ovariectomized rat model. Compared to TAM, it induced less increase in wet uterine wet weight and showed no uterotrophic effect. Compound **12** is a promising candidate for further development due to its inhibition activity on MCF-7 proliferation with moderate AlkP activity and no potential uterotrophic effects. The in vitro estrogenic activity encourages further investigations toward potential beneficial properties in cardiovascular, bone, and brain tissues.

## 1. Introduction

Selective estrogen receptor modulator (SERM) refers to a structurally diverse group of compounds that binds to both estrogen receptor subtypes ERα and/or ERβ despite lacking the estrogen steroid moiety. Whereas estrogens typically exert ER agonist effects, SERMs confer mixed functional ER agonist or antagonist activity depending on the target tissue [1]. An ideal SERM would have ER agonist activity in tissues where mimicking the action of estrogens is desirable (e.g., skeletal, cardiovascular, and central nervous systems), and lack of estrogenicity in tissues where estrogens have been shown to induce cancer initiation and growth (e.g., breast and endometrium) [2]. This definition led to investigations on the clinical profile of an ideal SERM. An ideal SERM prevents bone loss and fractures yet does not stimulate endometrial hyperplasia. It also provides relief of hot flushes and other menopausal symptoms. It should not increase the risk of coronary heart disease, stroke, or deep vein thrombosis. The first-generation triphenylethylene SERM included tamoxifen (TAM) and toremifene. Both SERMs are far from being ideal [3].

TAM (I) (a non-steroidal triphenylethylene derivative) was the first SERM approved as anti-estrogen for the treatment of metastatic breast cancer. It is now widely used as adjuvant chemotherapy for the treatment of hormone-dependent metastatic breast carcinoma in postmenopausal women. Although TAM (I) has been very successful in treating breast cancer, some side effects such as thromboembolic events, vasomotor symptoms, and an increased risk of endometrial hyperplasia are associated with TAM treatment [4].

**Figure 1:**
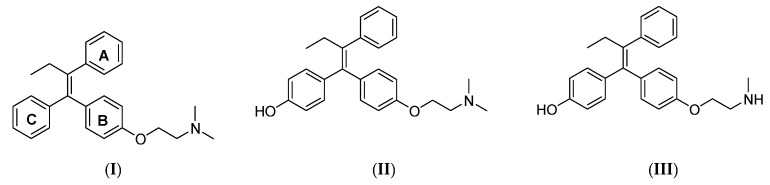
Structures of TAM (**I**), 4-OH-TAM (**II**), and endoxifen (**III**).

TAM (I) is regarded as a prodrug that is metabolized to the more active metabolites: 4-OH-TAM (II) and endoxifen (III) Figure 1 [5]. Compared to the parent drug, those metabolites have 100-times more affinity to the ER. This metabolism is mainly mediated via cytochrome P450 (CYP) enzymes, specifically the CYP2D6 and CYP3A4 isoforms. Pharmacogenetics revealed the polymorphic nature of the CYP2D6 enzyme. CYP2D6 poor metabolizers (based on CYP2D6*4 and *6) were reported to benefit less from TAM compared with extensive metabolizers [6,7,8].

The different phenotypes lead to different plasma concentrations of active metabolites among patients of different populations, and hence different clinical outcomes and may lead to drug resistance. Thus, to overcome TAM resistance, TAM is perceived as a clinical target in oncology personalized medicine [9,10,11].

On the hunt for novel SERMs that possess potential growth inhibitory activity on breast cancer cell lines yet lack the potential to induce endometrial carcinoma, we designed and synthesized 28 novel TAM analogs. The novel analogs bear a triphenylethylene scaffold. Modifications on rings **A**, **B**, and **C** aim to attenuate estrogenic/anti-estrogenic activities of the novel compounds so they can potentially inhibit breast cancer and provide positive estrogenic effects on bones and cardiovascular system without affecting endometrial tissues.

Structural modifications included introducing a chlorine atom at position 4 on ring **C** in all analogs; this ensures the blockage of the site of *para*-hydroxylation; thus, those analogs can bypass *para*-hydroxylation by polymorphic CYP2D6. The effect of this modification on the compounds estrogenic/anti-estrogenic properties is investigated.

The introduction of fluorine into a molecule can productively influence conformation, p*K*_a_, intrinsic potency, membrane permeability, metabolic pathways, and pharmacokinetic properties [12]. Based on these findings, ring **A** was kept unsubstituted or modified to 4-methoxy phenyl, 4-methoxy-3-fluoro phenyl, or 4-fluoro-3-methoxy phenyl. The introduction of a small, highly electronegative fluorine atom on ring **A** can affect the novel analogs stability and lipophilicity. The fluorine atom can further affect the binding affinity either directly or by affecting the polarity of the adjacent methoxy groups.

Previous literature focused on the effect of substitution on position 4 of ring **B** [13]; additionally, recent studies even suggested different substituents on ring **B** to design a homodimeric ER ligand that can act as ER antagonist and SERD (selective estrogen receptor degrader) [14]. In our work, the effect of the length of the alkoxy chain, size, and bulkiness of *N*-substituents and cyclization are thoroughly studied. The novel compounds were depicted in Table 1.

All synthesized compounds were tested for their relative activity in β-galactosidase in a yeast estrogen screen (YES) assay. Compounds were tested for their relative estrogenic/anti-estrogenic activities in comparison to positive and negative controls, respectively. YES assay is a gene reporter assay where the DNA sequence of human ERα is integrated into the yeast genome completed with an expression plasmid carrying estrogen response elements (ERE) in the promoter controlling the expression of the reporter gene *lacZ* (encoding the enzyme β-galactosidase). In the presence of estrogenic compounds, β-galactosidase is synthesized and secreted into the medium, where it converts the chromogenic substrate chlorophenol red-β-D-galactopyranoside (CRPG) from a yellow to a red product, whose absorbance is measured. Agonistic activity is measured directly, whereas antagonistic activity is measured in terms of reduction in color formation in the presence of 0.5 nM/1 nM estradiol (E2) [15,16,17]. Despite the ability of the YES assay to differentiate between agonists and antagonists, it becomes more and more apparent that compounds exhibit an organ-selective mode of action [18]. Therefore, we decided to test the novel compounds in an organ-specific in vitro model using the human Ishikawa endometrial adenocarcinoma cell line [19,20].

Alkaline phosphatase (AlkP) activity in these human endometrial cancer cells is markedly stimulated by estrogens [9]. In addition and in contrast to yeast assays, which do not mimic human metabolism, the Ishikawa cells, such as normal uterine cells, possess the important capacity to metabolize the compounds, which reflects their true estrogenic activity [21,22].

The anti-proliferative effects of the novel analogs were tested in vitro by the National Cancer Institute (NCI) on a panel of 60 human tumor cell lines at 10 µM. Compounds that elicited mean growth inhibition ≥50% were selected by the NCI for 5-dose testing. The concentration for 50% of maximal inhibition (GI_50_), total growth inhibition (TGI), and half-maximal lethal concentration (LC_50_) was measured for each cell line.

Compounds showing appreciable estrogenic activity in the YES assay and that were able to inhibit the growth of MCF-7 cancer cell lines yet with low estrogenic activity on Ishikawa endometrial adenocarcinoma cells might serve as potential ideal SERMS. Compounds **12** and **19** were therefore selected for the in vivo experiments to assess their estrogenic properties in an uterotrophic assay in an ovariectomized rat model. The in vivo uterotrophic rat assay is the gold standard assay to test for the estrogenic effect of compounds; the assay uses adult ovariectomized (OVX) female rats where there is no significant source of endogenous estrogens. Compounds that have estrogenic effects cause uterotrophic response due to the imbibition of water and growth of the uterine cells. Statistically significant uterine weight increases compared to controls provide a positive result [23,24,25,26,27,28,29]. Adopting both in vivo and in vitro assays was inevitable due to the limitations of each assay. The cell lines are not properly able to recapitulate the in vivo environment of the uterus within the body. On the other hand, the rat uterotrophic assay merely considers the uterine weight gain as an endpoint of estrogenicity without taking into account all factors that play a role in exerting an estrogenic effect on the organ and body [30].

All our compounds were biologically assayed as *E-Z* mixtures due to synthetic challenges and failure in separating the isomers using available chromatographic techniques. We adopted an in silico model to postulate the isomer with the lowest binding energy. The model also investigates the full agonistic activity of compound **3** despite the lack of an OH group on ring **C**. This group was reported to be essential for ER binding affinity of most synthetic ER ligands.

## 2. Results and Discussion

### 2.1. Chemistry Discussion

Compounds (**1**–**4**) were synthesized using standard McMurry coupling reaction of 4-Chloro-4-hydroxybenzophenone with commercially available ketones using titanium tetrachloride/zinc as a catalyst to yield four condensation products. The condensation products (**1**–**4**) were then treated with the appropriate base hydrochloride salts in dimethyl formamide (DMF) in the presence of potassium carbonate to form ethers (**5**–**28**) [31]. The formation of all compounds and their purity were confirmed via UPLC-ESI MS. All compounds were obtained as a mixture of E-Z isomers, as shown from UPLC-UV chromatograms. Some chromatograms showed distinct two peaks of nearly similar area (1:1.1) and having the same molecular ion peak (M+H)^+^. Attempts to isolate the E-Z isomers using column chromatography as well as preparative HPLC were not successful. ^1^H-NMR showed peaks integrating for double the number of protons, further confirming the formation of a mixture of E-Z isomers. ^13^C-NMR further confirmed the formation of isomers since most of the signals were duplicated. Such duplication of signals has been previously reported by Bedford and Richardson [32]. Their masses were confirmed via their molecular ion peaks [M+H]^+^ and [M+H+2]^+^ due to the presence of chlorine atoms in all compounds. As previously employed in similar work in the literature, compounds were assayed biologically as E-Z mixtures [33,34,35,36].

### 2.2. Anti-Estrogenic Assays

All compounds lacked significant anti-estrogenic on the ERα except compounds **27** and **28,** which were slightly able to antagonize the *β*-galactosidase reporter gene activity induced by 1 nM E2 by 11% and 12%, respectively. It seems that the para chlorine substitution at ring **C** has a detrimental effect on the anti-estrogenic activity. This modification has blocked the action of CYP2D6 and therefore prevented the formation of the anti-estrogenic hydroxy metabolite. It is reported that 4-OH-TAM and endoxifen, the active metabolites of TAM, have higher anti-estrogenic potency than the parent drug, TAM [33]. The OH group at position 4 of 4-OH-TAM is presumed to be responsible for its higher anti-estrogenic activity compared to TAM. Additionally, studies have reported that the anti-estrogenic property of SERMs depends on the ability of the cationic nitrogen on the alkylaminoethoxy side chain on ring **B** to neutralize the charge of Asp 351 [37]. Our results showed that the presence of a basic alkylaminoalkoxy group without a phenolic OH on ring **C** or a phenyl ring prone to metabolic hydroxylation could not elicit anti-estrogenic activity regardless of the size and basicity of this group, as shown in compounds **5**–**26**. Having no tertiary amino group on ring **B** as shown in compounds **3** and **4** or blocking position 4 on ring **C** as shown in compounds **5**–**26** will mostly abolish the anti-estrogenic action and shift it toward estrogenic activity (Table 2).

This drives us to the hypothesis that the alkylaminoethoxy side chain on ring **B** is not the only crucial factor for anti-estrogenicity. There are essentially two important features responsible for anti-estrogenic activity. A phenolic OH group is required for high-affinity binding to ER-forming crucial interactions (H-bonds) with Glu 353 and Arg 394 amino acids in the ligand-binding domain (LBD), and the alkylaminoalkoxy bulky group at ring **B** is essential for the ER antagonistic action where it forms a cationic interaction with Asp 351 amino acid of the ER [38].

### 2.3. Estrogenic Assays

All synthesized compounds were tested for their relative β-galactosidase activity in a yeast estrogen screen (YES) assay at a concentration of 1 µM using DMSO as control (set as 1). The hydroxylated analogs **3** and **4** showed EC_50_ values of 40.1 nM and 258 nM, respectively. E2, the endogenous ligand, showed an EC_50_ = 0.528 nM. The remarkable potency of the two novel analogs can be attributed to the introduction of a chloro group at the para position of ring **C**, the hydroxyl group of ring **B,** and the nature of the substituents on ring **A**.

Compound **3** (EC_50_ = 40 nM) bears a methoxy group at position 4 and a fluoro group at position 3 on ring **A**, and compound **3** showed six-fold more potency than its positional isomer compound **4** (EC_50_ = 258 nM). It seems that a methoxy group at position 4 is essential for agonistic activity.

This could further support the hypothesis that the introduction of a chloro group at ring **C** resulted in an estrogenic property, and the presence of an OH group at ring **B** allows better fitting into the receptor, ensures higher binding affinity, and locking the receptor drug complex into an agonistic conformation.

Replacing the OH group with different alkylaminoalkoxy side chains did not abolish the estrogenic action yet caused a decrease in activity. Comparing compounds (**5**–**9**) bearing a chloro group at ring **C**, unsubstituted ring **A** but different alkylaminoalkoxy side chains, compound **9** with an azepanethoxy side chain at ring **B** induced high relative β-galactosidase activity of 6.74 compared to control; a bulky cyclized side chain on ring **B** seems to improve estrogenic activity.

Compounds (**10**–**14**) bear a methoxy substituent on ring **A**. Both compounds **10** and **13** were the most potent congeners. They bear a dimethylaminopropoxy side chain and a morpholinylethoxy side chain, respectively, on ring **B** (relative β-galactosidase activity = 11.61 and 12.41, respectively). The para methoxy substituent led to an increase in relative estrogenic activity for compounds **10** and **13** compared to their congeners **5** and **8**. A remarkable decrease in relative estrogenic activity was observed for compound **14** compared to its congeners **9**; this may be explained by the fact that the bulky azepanylethoxy group displaced the methoxy substituent of ring **A** outside the binding pocket leading to a possible steric clash.

Compounds (**15**–**21**) bear 3-fluoro 4-methoxy on ring **A,** whereas compounds (**21**–**28**) bear 3-methoxy 4-fluoro substituents on ring **A**. The alkylaminoethoxy side chains on ring **B** were extended to include dimethylaminoethoxy and diethylaminoethoxy side chains. For all compounds (**15**–**21**), the addition of a fluoro group at position **3** enhances the relative estrogenic activity compared to their structural isomers (**22**–**28**) except for compound **18**. The unexpected behavior of compound **18** may be attributed to the less lipophilic character of this compound and lower pK_a_ value as a result of the morpholinylethoxy substituent on ring **B**. Compounds **15** and **17**, bearing a dimethylaminopropoxy side chain and a piperidinylethoxy side chain, respectively, showed relative estrogenic activities of 7.77 and 7.28, respectively. Compound **17** was the most potent among their series EC_50_ = 252 ± 8 nM. Comparing compound **17** with compound **12**, compound **17** was two-fold more estrogenic at 1 µM, the introduction of a fluoro group at the meta position had a positive impact on estrogenic activity. Compound **19** bearing azepanylethoxy group on ring **B** showed relative estrogenic activities of 3.22 and EC_50_ = 407 ± 86 nM, indicating that estrogenic activity is retained with bulky substituents. Compounds (**22**–**28**) were nearly equipotent. Modifying ring **A** to 3-methoxy 4-fluoro phenyl has resulted in a remarkable decrease in estrogenic activity. It seems that the methoxy substituent at the para position and fluoro substituent at the meta position of ring **A** is the main determinant factors for the higher agonistic action rather than the size or cyclization of substituents on ring **B** (Table 3 and Table 4).

### 2.4. NCI Growth Inhibition Assays

Compounds were submitted to the Developmental Therapeutics Program (DTP) of the National Cancer Institute (NCI). The program uses a panel of 60 human tumor cell lines representing nine tissue types, including leukemia, non-small cell lung cancer (NSCLC), melanoma, colon cancer, ovarian cancer, CNS cancer, renal cancer, prostate cancer, and breast cancer, to screen for potential new anti-cancer agents. SRB (sulforhodamine B) assay is the preferred high-throughput assay of the National Cancer Institute (NCI) and is the assay used in the NCI’s lead compound screening program. Primary screening of synthesized compounds was performed by testing a single high dose of 10 µM in the full NCI-60 panel. The percent growth of treated cells relative to the no-drug control and relative to the time zero number of cells was measured, and a mean graph was provided. The percentage inhibition was then calculated by subtracting the values obtained from 100. In general, all compounds bearing an OH group (**3** and **4**) or a morpholinylethoxy side chain on ring **B** (**8**, **13**, **18,** and **25**) lacked anti-proliferative activity. They had the least percent mean growth inhibition and the lowest percent inhibition on human breast cancer MCF-7 cells. This may be attributed to the partial hydrophilicity of ring **B** (Table 5).

Six compounds: **11** (67.76%), **12** (55.21%), **16** (77.24%), **17** (69.12%), **19** (60.79%), **28** (92.33%), showed mean percentage inhibition on all 60 cell lines higher than 50% and were escalated to a dose-dependency assay using five doses on the 60 cell panel. Five of the six compounds share two common features; they bear a para methoxy substituent on ring **A** and bear a cyclic alkylaminoethoxy group on ring **B**.

In the dose-dependency assay, compounds were evaluated against the 60-cell panel at the five doses; 10^−4^ M, 10^−5^ M, 10^−6^ M, 10^−7^ M, and 10^−8^ M. Dose-response curves for each cell line was drawn, and three response parameters are extracted by linear interpolation (GI_50_, TGI, LC_50_).

To investigate SERM-like properties of compounds, looking at results from ER-positive cell lines is particularly important. The two most potent compounds on Erα-positive MCF-7 breast cancer cell line were compounds **11** (GI_50_ = 0.89 µM) and **12** (GI_50_ = 0.60 µM). They are almost twice as active as TAM (GI_50_ = 1.58 µM; see Supplementary Materials). Both compounds bear a para methoxy substitution on ring **A** and a cyclic aminoethoxy group on ring **B,** namely a pyrolidine and piperidine, consecutively. The incorporation of the basic nitrogen in a cyclic structure enhances its basicity and significantly improves the anti-proliferative effect of the compounds.

Compounds **16**, **17,** and **19** showed GI_50_ = 2.41, 3.34, and 3.59 µM, respectively. Those compounds bear a para methoxy substituent and a meta fluorine substituent on ring **A**. They exhibited lower anti-proliferative activity on the MCF-7 breast cancer cell line compared to their congeners that lack a fluorine group on meta position, e.g., compounds **11**, **12**, and **14**. This suggests that the presence of a methoxy group increased the electron density on ring **A** resulting in a better anti-proliferative activity, whereas the introduction of an electron-withdrawing group such as fluorine at the meta position lowered the activity. We presumed that introduction of fluorine will increase compounds lipophilicity and therefore improve compounds’ cellular uptake and growth inhibition potential. Switching the positions of the methoxy and fluorine substituents in compounds (**22**–**28**) deteriorated the anti-proliferative activity except in compound **28** (GI_50_ = 2.17 µM). This further confirms that fluorine develops essential interactions with specific targets involved in novel compounds’ cytotoxic activities.

**Figure 2:**
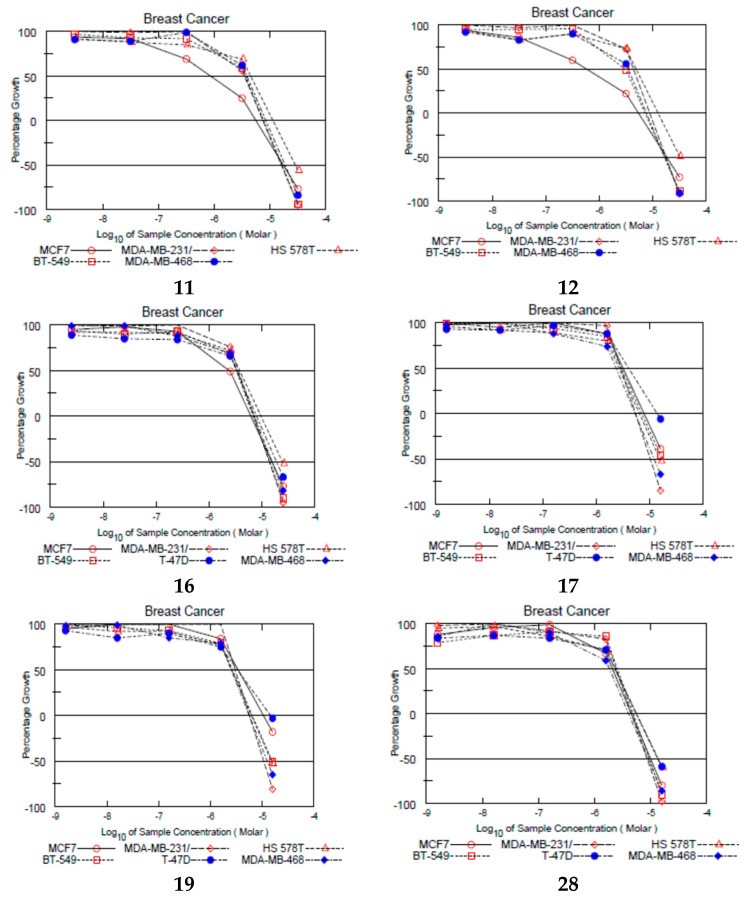
Dose-response curves of selected compounds on breast cancer cell lines. Data obtained from NCI in vitro disease-oriented human tumor cell screen (for details, see the work of [39]) compounds were tested in triplicates.

It is worth mentioning that all six escalated compounds showed more potent anti-proliferative activity than TAM on triple-negative breast cancer (TNBC) cell lines MDA-MDB-231/ATCC and BT-549. Compounds **17**, **19,** and **28** were more potent than TAM on Hs578T, whereas only compound **28** was equipotent to TAM on MDA-MB-468. Since TNBC cell lines do not express ER, this suggests that these novel TAM analogs elicit their anti-proliferative activity via a mechanism that does not involve binding to ER. The six compounds also exhibited mild to high estrogenic activity, but with anti-proliferative activity, this offers an advantage over existing SERM such as TAM (Figure 2).

The ability of the compounds to inhibit the growth of other panels rather than breast cancer was investigated. All six compounds were found to be three times more active than TAM (mean GI_50_ = 6.31 µM) on the colon cancer cell lines with (mean GI_50_ = 1.90 µM). TAM was reported to inhibit the growth of colon cancer cells, yet the mechanism of inhibition is not clear yet, and further studies are warranted before any clinical implications can be postulated (see Supplementary Materials).

Compound **28** (mean GI_50_ = 2.34 µM) was approximately three times as potent as TAM (mean GI_50_ = 6.31 µM) on NSCLC cell lines, and twice as potent as TAM (mean GI_50_ = 5.00 and 5.35 µM) on both renal (mean GI_50_ = 2.40 µM) and prostate (mean GI_50_ = 2.31 µM) cell lines. Compound **28** showed an exceptional broad-spectrum growth inhibition.

**Figure 3:**
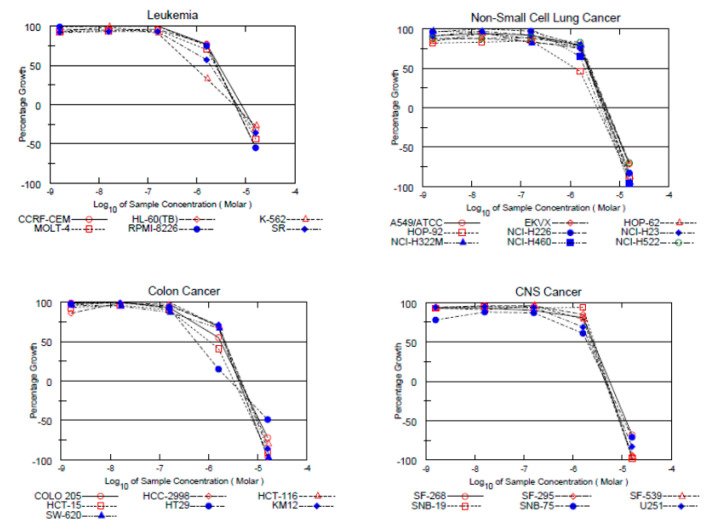

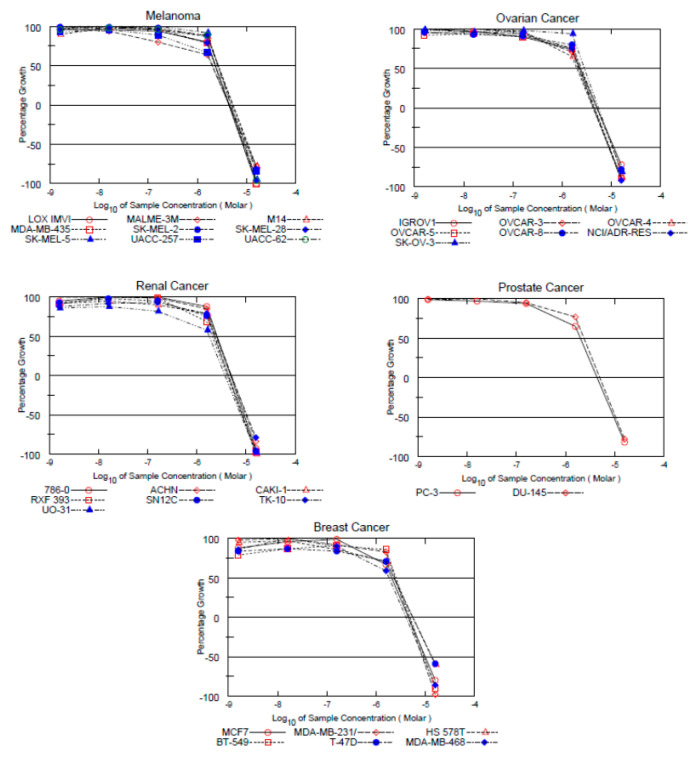
Dose-response curves of compound **28** on different subpanels. Data obtained from NCI in vitro disease-oriented human tumor cell screen (for details, see the work of [39]) compounds were tested in triplicates.

The six compounds showed the highest potency on colon cancer cell lines; this might indicate some selectivity toward this particular panel. Further investigations might help understand the reason for this selectivity (Figure 3).

### 2.5. Alkaline Phosphatase Activity in Ishikawa Cell Line

Because of the potential SERM character of the compounds tested, their estrogenic effects were studied in an endometrial-derived cell culture model, the human endometrial adenocarcinoma cell line Ishikawa. Estrogenic compounds are able to increase the alkaline phosphatase (AlkP) activity mediated by the ERα. All compounds were screened at two concentrations, 0.1 and 1 µM. Its agonistic effect was compared to the vehicle control DMSO (data shown in Supplementary Materials). Estradiol at 10 nM was used as a positive control and TAM and OH-TAM at 1 µM as comparative controls. Most of the compounds showed no significant increase in AlkP activity after a 72 h treatment. Compounds **5**, **11**, **12,** and **19** showed moderate estrogenic activity in YES assay and growth inhibition above 50% on MCF-7 cells at 10 µM; therefore, they were selected for the 5-dose AlkP assay. The four compounds were studied in a concentration range of 1 nM to 10 µM. Compounds **11**, **12**, and **19** were able to increase the AlkP activity in a dose-pendent manner with significant effects at a concentration of 100 nM and 1 µM. No significant effects were observed for compound **5**. The decreased activities at a concentration of 10 µM are caused by a negative influence of the treatment on the cell growth, observed with light microscopy. Compound **12** showed an equipotent activity when compared to TAM and 4-OH-TAM despite its higher relative estrogenic activity in the YES assay (Table 6).

The observed moderate estrogenic effects of **11**, **12**, and **19** endorse the results obtained by the other in vitro assays reported. Using this Ishikawa cell culture model only gives a hint about possible effects on uterine tissue and needs more investigations.

### 2.6. Uterotrophic Assay

The most common short-term in vivo assay for estrogenicity/anti-estrogenicity is the uterotrophic assay, suitable for screening ERα agonists and antagonists. The primary endpoint is the uterine wet weight (UWW). An increase in UWW indicates an estrogenic activity of the test compound. Compounds **12** and **19** were screened using the in vivo uterotrophic assay. Both compounds showed less increase in UWW, indicating lower endometrial estrogenic activity and potentially less tendency to induce endometrial carcinoma (Table 7).

### 2.7. In Silico Study

The most potent estrogenic compound **3** (EC_50_ = 40.1 nM) bearing an OH group at the para position of ring **B** and 3-fluoro 4-methoxy substituents on ring **A** was selected for the in silico model. Compound **3** was docked into ERα LBD co-crystallized with diethylstilbestrol (DES), a synthetic estrogen with full agonistic activity (PDB: 3ERD) [40]. To validate the docking protocol, the co-crystallized ligand DES was docked into the ERα LBD where all the resultant poses converged to a similar binding mode as that of the experimentally determined position of DES with the best ranking pose having an RMSD value of 1.71 Å.

The crystal structures of ERα bound to DES (PDB code: 3ERD) [9] were downloaded from the PDB database. Only protein molecules were considered where it was optimized using the structure preparation wizard in MOE (version 2009.10) [38] and saved as a mol file. DES was built as E-isomer, whereas compound **3** was built as pure E and Z isomers, minimized using the MMFF94x force field in MOE using a gradient of 0.0001 kcal/(mol Å), and their protonation states at pH 7.0 were generated. A conformational search was adopted for compound **3E** and **3Z** isomers and E-DES. The database obtained was saved as.mdb and used as docking ligands.

**Figure 4:**
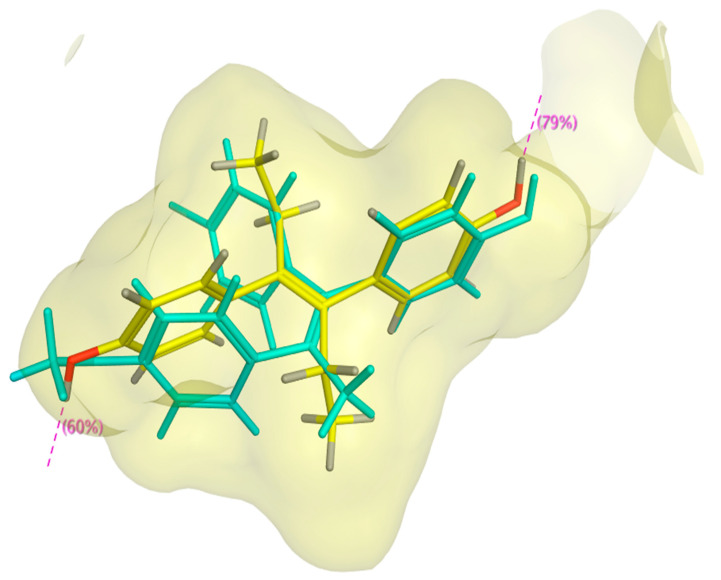
Compound **3*E*** (**cyan**) overlaid with DES (**yellow**) inside ERα LBD.

Results of the overlay of compounds **3E** and **3Z** on DES showed that the **3E** conformer with the lowest binding energy showed a partial overlay on DES (Figure 4).

**Figure 5:**
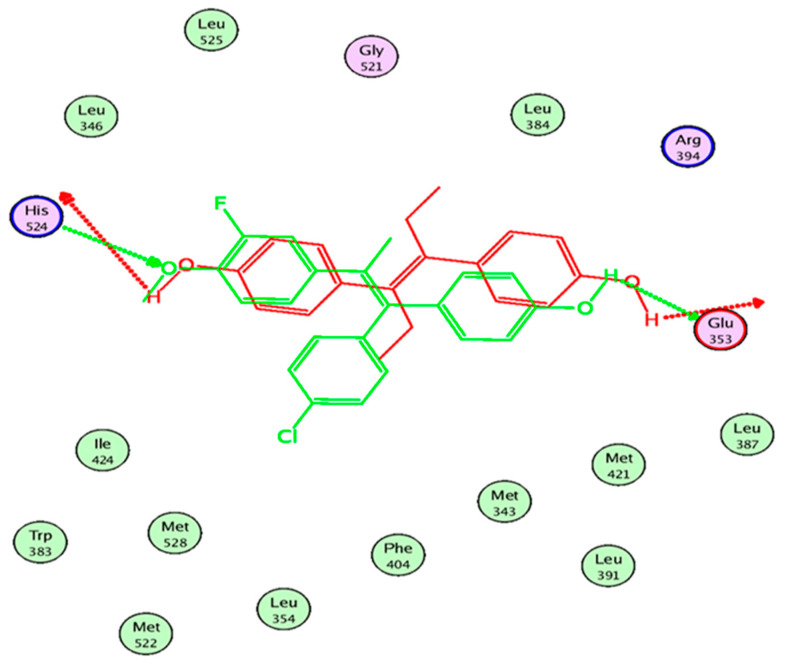
Two-dimensional interactions of DES (**red**) and compound **3E** (**green**) inside ERα LBD.

Compound **3E** retained the two essential interactions with Glu353 and His524, the oxygen of the methoxy group on ring **A** of compound **3E** acted as H-bond acceptor rather than H-bond donor (Figure 5).

## 3. Experimental Section

### 3.1. Chemistry

All reactions were carried out under nitrogen when an inert atmosphere was needed. Syntheses that required dry and oxygen-free conditions were performed in a Glovebox MB Unilab or using Schlenk techniques under an atmosphere of purified nitrogen or argon, respectively. Dry, oxygen-free solvents (CH_2_Cl_2_, distilled from CaH_2_; THF, distilled from potassium) were employed. All distilled and deuterated solvents were stored over molecular sieves (4 Å). All glassware was oven-dried at 160 °C prior to use. Solvents and reagents were obtained from commercial suppliers and were of pure analytical grade. Purification of compounds was carried out using column chromatography with silica gel 40– 60 μM mesh or using a Biotage*^®^* Isolera™ (Uppsala, Sweden) flash purification system using Biotage*^®^* KP-Sil SNAP columns. Reaction progress was monitored by TLC using fluorescent pre-coated silica gel plates, and detection of the components was made by short UV light (λ = 254 nm).

^1^H-NMR spectra were measured on either 400 MHz Bruker or on a Bruker AVANCE III HD Nanobay, 400 MHz UltraSield (^1^H (400.13 MHz), ^13^C (100.61 MHz)) or on a Bruker AVANCE III HDX, 500 MHz Ascend (^1^H (500.13 MHz), ^13^C (125.75 MHz)) spectrometer. All ^13^C NMR spectra were exclusively recorded with composite pulse decoupling. Chemical shifts were referenced to δ_TMS_ = 0.00 ppm. (^1^H, ^13^C) Chemical shifts (δ) are reported in ppm. Coupling constants (J) are reported in Hz. Multiplicities are abbreviated as: s: singlet; d: doublet; t: triplet; q: quartet; m: multiplet; dd: doublet of doublet; dt: doublet of triplet; brs: broad singlet. Mass spectrometric analysis (UPLC-ESI-MS) was performed using Waters ACQUITY Xevo TQD system, which consisted of an ACQUITY UPLC H-Class system and Xevo^TM^ TQD triple-quadrupole tandem mass spectrometer with an electrospray ionization (ESI) interface (Waters Corp., Milford, MA, USA). Acquity BEH C18 100 × 2.1 mm column (particle size, 1.7 µm) was used to separate analytes (Waters, Dublin, Ireland). The solvent system consisted of water containing 0.1% TFA (A) and 0.1% TFA in acetonitrile (B). UPLC-method: flow rate 200 μL/min. The percentage of B started at an initial of 5% and maintained for 1 min, then increased up to 100% during 10 min, kept at 100% for 2 min, and flushed back to 5% in 3 min. The MS scan was carried out at the following conditions: capillary voltage 3.5 kV, cone voltage 20 V, radio frequency (RF) lens voltage 2.5 V, source temperature 150 °C, and desolvation gas temperature 500 °C. Nitrogen was used as the desolvation and cone gas at a flow rate of 1000 and 20 L/h, respectively. System operation and data acquisition were controlled using Mass Lynx 4.1 software (Waters).

#### 3.1.1. General Procedures for Preparation of Compound **1**–**4**

Zinc powder (10.11 g, 154 mmol) was suspended in dry THF (100 mL), and the mixture was cooled to 0 °C. TiCl_4_ (7.5 mL, 70 mmol) was added dropwise under nitrogen/argon. When the addition was complete, the mixture was warmed to room temperature and heated to reflux for 2 h. After cooling down, a solution of 4-Chloro-4-hydroxybenzophenone (2.86 g, 12.3 mmol) and acetophenone/4′-methoxyacetophenone/3′-Fluoro-4′-methoxyacetophenone/4′-Fluoro-3′-methoxyacetophenone (38.4 mmol) in dry THF (100 mL) was added at 0 °C, and the mixture was heated at reflux in the dark for 2.5–7 h. After being cooled to room temperature, the zinc dust was filtered off, and THF was removed under reduced pressure. The residue was dissolved in an aqueous solution containing 30% hydrochloric acid (500 mL) and then extracted with dichloromethane (120 mL × 6). The organic layers were combined and dried over anhydrous Na_2_SO_4_, concentrated in vacuo, and further purified by silica gel column chromatography or a Biotage*^®^* Isolera™ flash purification system using Biotage*^®^* KP-Sil SNAP columns (dichloromethane) to yield compounds **1**–**4** [34].

##### *E/Z*-4-[1-(4-Chloro-phenyl)-2-phenylpropenyl]-phenol (**1**) 

C_21_H_17_ClO. Yield: 58%. Orange oil. Purity: 100%. ^1^H-NMR (400 MHz, CDCl_3_) δ: 7.33 (d, J = 2.0 Hz, 2H), 7.32 (d, J = 2.0 Hz, 2H), 7.20–7.11 (m, 12H), 6.83 (d, J = 1.5 Hz, 1H), 6.80 (d, J = 1.5 Hz, 1H), 6.74 (d, J = 2.1 Hz, 2H), 6.72 (d, J = 2.1 Hz, 2H), 6.50 (d, J = 2.1 Hz, 2H), 6.49 (d, J = 2.1 Hz, 2H), 2.15 (s, 2H), 2.12 (s, 6H). ^13^C-NMR (101 MHz, CDCl_3_) δ: 154.28, 153.60, 143.91, 143.82, 142.12, 141.78, 137.55, 137.51, 136.13, 135.67, 135.51, 135.34, 132.33, 132.18, 132.14, 131.52, 131.39, 129.19, 128.30, 128.03, 127.95, 127.58, 126.33, 126.23, 115.04, 114.45, 23.49, 23.30. MS (ESI): *m/z* = 321.1 [M+H]^+^ (100%), *m/z* = 323.1 [M+H+2]^+^ (33%). R_f_: 0.42 (100% methylene chloride).

##### *E/Z*-4-[1-(4-Chloro-phenyl)-2-(4-methoxy-phenyl)-propenyl]-phenol (**2**)

C_22_H_19_ClO_2_. Yield: 55%. Orange oil. Purity: 95%. ^1^H-NMR (400 MHz, CDCl_3_) δ: 7.32 (d, J = 2.0 Hz, 1H), 7.30 (d, J = 1.9 Hz, 1H), 7.18 (d, J = 2.0 Hz, 1H), 7.16 (d, J = 1.8 Hz, 1H), 7.13–6.99 (m, 10H), 6.88 (d, J = 2.2 Hz, 1H), 6.87 (d, J = 2.1 Hz, 1H), 6.83 (d, J = 1.7 Hz, 2H), 6.81 (d, J = 1.8 Hz, 2H), 6.74–6.71 (m, 2H), 6.54 (d, J = 2.2 Hz, 1H), 6.52 (d, J = 2.0 Hz, 1H), 3.76 (d, J = 2.7 Hz, 6H), 2.58 (s, 2H), 2.12 (d, J = 5.0 Hz, 6H). ^13^C-NMR (101 MHz, CDCl_3_) δ: 158.33, 157.94, 154.53, 153.80, 142.45, 142.15, 137.09, 137.02, 136.16, 136.07, 135.68, 135.65, 135.47, 135.39, 132.16, 132.12, 131.43, 131.31, 130.75, 130.36, 130.34, 130.15, 128.26, 127.61, 115.06, 114.55, 113.76, 113.72, 113.42, 113.34, 55.49, 55.15, 23.45, 23.24. MS (ESI): *m/z* = 351.1 [M+H]^+^ (100%) *m/z* = 353.1 [M+H+2]^+^ (33%). R_f_: 0.33 (100% methylene chloride).

##### *E/Z*-4-[1-(4-Chlorophenyl)-2-(3-fluoro-4-methoxyphenyl) propenyl]-phenol (**3**)

C_22_H_18_ClFO_2_. Yield: 70%. Orange oil. Purity: 95%. ^1^H-NMR (500 MHz, CDCl_3_) (δ 7.33–7.29 (m, 2H), 7.16–7.13 (m, 2H), 7.08–7.05 (m, 2H), 7.04–7.00 (m, 2H), 6.90–6.88 (m, 1H), 6.88–6.85 (m, 1H), 6.83–6.81 (m, 3H), 6.81–6.79 (m, 2H), 6.77–6.76 (m, 1H), 6.76–6.73 (m, 2H), 6.73–6.71 (m, 2H), 6.55–6.51 (m, 2H), 5.30 (s, 2H), 3.84 (d, J = 1.7 Hz, 6H), 2.10 (s, 3H), 2.06 (s, 3H). ^13^C-NMR (126 MHz, CDCl_3_) δ 154.47, 153.87, 151.89 (d, J = 245.2 Hz), 151.86 (d, J = 244.9 Hz), 145.94 (t, J = 10.7 Hz), 142.01, 141.70, 137.94, 136.89 (d, J = 6.3 Hz), 136.80 (d, J = 6.3 Hz), 135.45, 135.14, 134.43 (d, J = 1.4 Hz), 133.80 (d, J = 1.4 Hz), 132.42, 132.09, 132.04, 131.71, 131.33, 128.34, 127.78, 125.21 (d, J = 3.3 Hz), 125.13 (d, J = 3.3 Hz), 116.92 (d, J = 18.4 Hz), 116.86 (d, J = 18.3 Hz), 115.08, 114.66, 112.82 (d, J = 8.3 Hz), 112.81 (d, J = 8.3 Hz), 58.59, 56.16, 23.29, 23.09. MS (ESI): *m/z* = 368.83 [M+H]^+^, *m/z* = 370.83 [M+H+2]^+^. R_f_: 0.45 (100% methylene chloride).

##### *E/Z*-4-[1-(4-Chlorophenyl)-2-(4-fluoro-3-methoxyphenyl) propenyl]-phenol (**4**)

C_22_H_18_ClFO_2_. Yield: 57%. Orange oil. Purity: 97%. ^1^H-NMR (400 MHz, CDCl_3_) δ 7.32 (d, J = 8.3 Hz, 2H), 7.17 (d, J = 8.3 Hz, 2H), 7.08 (d, J = 8.5 Hz, 2H), 7.03 (d, J = 8.4 Hz, 2H), 6.94–6.86 (m, 2H), 6.83 (t, J = 7.8 Hz, 3H), 6.78–6.68 (m, 5H), 6.65 (td, J = 8.2, 1.8 Hz, 2H), 6.54 (d, J = 8.5 Hz, 2H), 3.63 (d, J = 4.4 Hz, 6H), 2.19 (s, 4H), 2.12 (d, J = 13.8 Hz, 2H), 2.10 (s, 3H). ^13^C-NMR (101 MHz, CDCl_3_) δ 154.49, 153.91, 151.00 (d, J = 245.6 Hz), 150.93 (d, J = 245.4 Hz), 146.88 (d, J = 10.8 Hz), 146.80 (d, J = 10.8 Hz), 141.88, 141.84, 140.01 (d, J = 4.0 Hz), 139.97 (d, J = 4.0 Hz), 138.01, 138.00, 135.31, 135.25, 135.09, 134.51, 132.47, 132.00, 131.95, 131.76, 131.31, 128.35, 127.77, 121.47 (d, J = 6.6 Hz), 121.36 (d, J = 6.6 Hz), 115.55 (d, J = 18.2 Hz), 115.44 (d, J = 18.3 Hz), 115.19, 115.16, 115.10, 114.64, 56.08, 56.04, 23.22, 23.00. MS (ESI): *m/z* = 368.83 [M+H]^+^ (100%), *m/z* = 370.83 [M+H+2]^+^. R_f_: 0.37 (100% methylene chloride).

#### 3.1.2. General Procedures for Preparation of Compounds **5**–**28**

A solution of compounds **1**–**4** (16.28 g, 47 mmol) in DMF (100 mL) was treated with K_2_CO_3_ (19.5 g, 141 mmol) and heated in an oil bath at 80 °C. The resulting suspension was treated with the appropriate commercially available base hydrochloride salt (51 mmol) portion-wise over a 2 h period and stirred for 16 h. The reaction mixture was cooled to room temperature. K_2_CO_3_ was filtered off, and DMF was removed under reduced pressure. The final product was purified by silica gel column chromatography or a Biotage*^®^* Isolera™ flash purification system using Biotage*^®^* KP-Sil SNAP columns (dichloromethane) to yield compounds (**5**–**28**).

##### *E/Z*-(3-{4-[1-(4-Chloro-phenyl)-2-phenyl-propenyl]-phenoxy}-propyl)-dimethyl-amine (**5**)

C_26_H_28_ClNO. Yield: 48%. Brown oil. Purity: 98%. ^1^H-NMR (400 MHz, CDCl_3_) δ: 7.30 (d, J = 8.4 Hz, 2H), 7.19–7.09 (m, 16H), 6.97 (d, J = 8.5 Hz, 2H), 6.86 (d, J = 8.6 Hz, 4H), 6.80–6.73 (m, 2H), 4.06 (t, J = 5.9 Hz, 2H), 3.91 (t, J = 5.9 Hz, 2H), 2.90 (m, 2H), 2.83 (m, 2H), 2.59 (s, 6H), 2.55 (s, 6H), 2.18–2.16 (dd, J = 9.6, 6.1 Hz, 2H), 2.14–2.08 (m, 8H). ^13^C-NMR (101 MHz, CDCl_3_) δ: 141.76, 135.78, 132.15, 131.97, 131.37, 131.23, 129.18, 129.16, 128.28, 128.00, 127.94, 127.55, 126.32, 126.22, 114.02, 113.38, 65.30, 56.11, 56.05, 44.18, 44.07, 25.95, 25.78, 23.48, 23.33. MS (ESI): *m/z* = 406.3 [M+H]^+^ (100%), *m/z* = 408.2 [M+H+2]^+^ (33%). R_f_: 0.43 (9:1 methylene chloride: methanol).

##### *E/Z*-1-(2-{4-[1-(4-Chloro-phenyl)-2-phenyl-propenyl]-phenoxy}-ethyl)-pyrrolidine (**6**)

C_27_H_28_ClNO. Yield: 44%. Faint brown oil. Purity: 95.84%. ^1^H-NMR (400 MHz, CDCl_3_) δ: 7.33–7.29 (m, J = 2.0 Hz, 2H), 7.20–7.08 (m, 14H), 6.99–6.96 (m, 2H), 6.91–6.87 (m, 2H), 6.81–6.73 (m, 4H), 6.68–6.57 (m, 2H), 4.23 (t, J = 5.6 Hz, 2H), 4.09 (t, J = 5.6 Hz, 2H), 3.09 (t, J = 5.6 Hz, 2H), 3.01 (t, J = 5.5 Hz, 2H), 2.85 (d, J = 18.8 Hz, 8H), 2.13 (s, 3H), 2.10 (s, 3H), 1.96–1.84 (m, 8H). ^13^C-NMR (101 MHz, CDCl_3_) δ: 157.17, 156.46, 143.84, 143.79, 142.13, 141.77, 137.52, 137.45, 136.14, 135.84, 135.51, 132.29, 132.18, 131.98, 131.48, 131.41, 131.23, 129.20, 129.17, 128.28, 128.01, 127.97, 127.56, 126.32, 126.25, 114.15, 113.51, 65.95, 65.65, 54.77, 54.69, 54.63, 54.58, 23.50, 23.39, 23.34. MS (ESI): *m/z* = 418.3 [M+H]^+^ (100%), *m/z* = 420.3 [M+H+2]^+^ (33%). R_f_: 0.5 (9:1 methylene chloride: methanol).

##### *E/Z*-1-(2-{4-[1-(4-Chloro-phenyl)-2-phenyl-propenyl]-phenoxy}-ethyl)-piperidine (**7**)

C_28_H_30_ClNO. Yield: 42%. Faint brown oil. Purity: 97.82%. ^1^H-NMR (400 MHz, CDCl_3_) δ: 7.32 (d, J = 2.0 Hz, 1H), 7.30 (d, J = 1.6 Hz, 1H), 7.20–7.09 (m, 14H), 6.98 (d, J = 1.9 Hz, 1H), 6.97 (d, J = 2.0 Hz, 1H), 6.89 (d, J = 2.0 Hz, 1H), 6.87 (d, J = 2.0 Hz, 1H), 6.80 (d, J = 2.0 Hz, 1H), 6.79 (d, J = 1.9 Hz, 1H), 6.76 (d, J = 2.0 Hz, 1H), 6.74 (d, J = 2.1 Hz, 1H), 6.56 (d, J = 2.1 Hz, 1H), 6.55 (d, J = 2.0 Hz, 1H), 4.20 (t, J = 5.8 Hz, 2H), 4.05 (t, J = 5.7 Hz, 2H), 2.91 (t, J = 5.7 Hz, 2H), 2.82 (t, J = 5.7 Hz, 2H), 2.72–2.55 (m, 8H), 2.14 (s, 3H), 2.10 (s, 3H), 1.72–1.64 (m, 8H), 1.52–1.44 (m, 4H). ^13^C-NMR (101 MHz, CDCl_3_) δ: 157.30, 156.61, 143.87, 143.80, 142.16, 141.79, 137.56, 137.49, 136.09, 135.69, 135.45, 135.34, 132.29, 132.18, 131.95, 131.48, 131.41, 131.21, 129.20, 129.18, 128.28, 128.01, 127.96, 127.56, 126.31, 126.23, 114.13, 113.52, 65.28, 65.03, 57.67, 57.58, 54.87, 54.80, 25.34, 25.23, 23.75, 23.68, 23.50, 23.33. MS (ESI): *m/z* = 432.3 [M+H]^+^ (100%), *m/z* = 434.3 [M+H+2]^+^ (33%). R_f_: 0.37 (93:7 methylene chloride: methanol).

##### *E/Z*-4-(2-{4-[1-(4-Chloro-phenyl)-2-phenyl-propenyl]-phenoxy}-ethyl)-morpholine (**8**)

C_27_H_28_ClNO_2_. Yield: 48%. Orange oil. Purity: 100%. ^1^H-NMR (400 MHz, CDCl_3_) δ: 7.32 (d, J = 1.9 Hz, 1H), 7.31 (d, J = 2.0 Hz, 1H), 7.20–7.09 (m, 14H), 6.99 (d, J = 1.9 Hz, 1H), 6.97 (d, J = 2.0 Hz, 1H), 6.90 (d, J = 2.0 Hz, 1H), 6.88 (d, J = 2.0 Hz, 1H), 6.81 (d, J = 2.0 Hz, 1H), 6.79 (d, J = 1.9 Hz, 1H), 6.76 (d, J = 2.0 Hz, 1H), 6.75 (d, J = 2.1 Hz, 1H), 6.57 (d, J = 2.1 Hz, 1H), 6.56 (d, J = 2.0 Hz, 1H), 4.14 (t, J = 5.7 Hz, 2H), 4.00 (t, J = 5.7 Hz, 2H), 3.76 (m, 4H), 3.72 (m, 4H), 2.84 (t, J = 5.7 Hz, 2H), 2.74 (t, J = 5.7 Hz, 2H), 2.62 (m, 4H), 2.55 (m, 4H), 2.14 (s, 3H), 2.11 (s, 3H). ^13^C-NMR (101 MHz, CDCl_3_) δ: 157.44, 156.78, 143.90, 143.80, 142.17, 141.80, 137.57, 137.51, 136.09, 135.67, 135.44, 135.28, 132.30, 132.18, 131.93, 131.49, 131.40, 131.20, 129.20, 129.19, 128.29, 128.02, 127.95, 127.56, 126.32, 126.21, 114.14, 113.53, 66.85, 66.80, 65.62, 65.41, 57.66, 57.62, 54.06, 54.02, 23.50, 23.34. MS (ESI): *m/z* = 434.3 [M+H]^+^ (100%), *m/z* = 436.3 [M+H+2]^+^ (33%). R_f_: 0.68 (95:5 methylene chloride: methanol).

##### *E/Z*-1-(2-{4-[1-(4-Chloro-phenyl)-2-phenyl-propenyl]-phenoxy}-ethyl)-azepane (**9**)

C_29_H_32_ClNO. Yield: 40%. Brown oil. Purity: 95.34%. ^1^H-NMR (400 MHz, CDCl_3_) δ: 7.32 (d, J = 1.9 Hz, 1H), 7.30 (d, J = 1.9 Hz, 1H), 7.20–7.08 (m, 14H), 6.99–6.95 (m, 2H), 6.88 (dd, J = 6.7, 4.8 Hz, 2H), 6.80 (d, J = 1.9 Hz, 1H), 6.78 (d, J = 1.9 Hz, 1H), 6.76 (d, J = 2.0 Hz, 1H), 6.74 (d, J = 2.0 Hz, 1H), 6.56 (d, J = 1.9 Hz, 1H), 6.54 (d, J = 2.0 Hz, 1H), 4.21 (t, J = 5.7 Hz, 2H), 4.06 (t, J = 5.7 Hz, 2H), 3.12 (t, J = 5.7 Hz, 2H), 3.04 (t, J = 5.7 Hz, 2H), 3.00–2.89 (m, 8H), 2.14 (s, 3H), 2.10 (s, 3H), 1.80–1.58 (m, 16H). ^13^C-NMR (101 MHz, CDCl_3_) δ: 156.76, 156.25, 143.78, 141.76, 136.13, 132.17, 131.97, 131.48, 131.40, 131.23, 129.19, 129.17, 128.29, 128.01, 127.96, 127.56, 126.31, 126.24, 114.15, 113.52, 56.32, 56.20, 55.69, 55.61, 26.96, 26.92, 26.57, 23.50, 23.33. MS (ESI): *m/z* = 446.3 [M+H]^+^ (100%), *m/z* = 448.3 [M++H+2]^+^ (33%). R_f_: 0.37 (93:7 methylene chloride: methanol).

##### *E/Z*-(3-{4-[1-(4-Chloro-phenyl)-2-(4-methoxy-phenyl)-propenyl]-phenoxy}-propyl)-dimethyl-amine (**10**)

C_27_H_30_ClNO_2_. Yield: 48%. Orange oil. Purity: 96.57%. ^1^H-NMR (400 MHz, CDCl_3_) δ: 7.30 (d, J = 1.9 Hz, 1H), 7.28 (d, J = 1.9 Hz, 1H), 7.16–6.96 (m, 12H), 6.87–6.68 (m, 8H), 6.56 (d, J = 2.1 Hz, 1H), 6.54 (d, J = 2.0 Hz, 1H), 4.05 (t, J = 6.0 Hz, 2H), 3.92 (t, J = 6.0 Hz, 2H), 3.75 (s, 6H), 2.84–2.73 (m, 4H), 2.51 (d, J = 10.2 Hz, 6H), 2.20–2.11 (m, 4H), 2.10 (s, 6H), 2.07 (s, 6H). ^13^C-NMR (101 MHz, CDCl_3_) δ: 157.96, 157.87, 157.32, 156.59, 142.39, 142.08, 136.96, 136.88, 136.05, 135.96, 135.93, 135.65, 135.54, 134.89, 132.21, 131.99, 131.42, 131.29, 131.24, 130.33, 130.31, 128.26, 127.60, 113.99, 113.43, 113.37, 113.31, 65.42, 65.16, 56.21, 56.16, 55.13, 53.44, 44.47, 44.35, 26.28, 26.13, 23.43, 23.28. MS (ESI): *m/z* = 436.3 [M+H]^+^ (100%), *m/z* = 438.3 [M+H+2]^+^ (33%). R_f_: 0.45 (9:1 methylene chloride: methanol).

##### *E/Z*-1-(2-{4-[1-(4-Chloro-phenyl)-2-(4-methoxy-phenyl)-propenyl]-phenoxy}-ethyl)-pyrrolidine (**11**)

C_28_H_30_ClNO_2_. Yield: 40%. Orange oil. Purity: 100%. ^1^H-NMR (400 MHz, CDCl_3_) δ: 7.31 (d, J = 1.9 Hz, 1H), 7.29 (d, J = 1.9 Hz, 1H), 7.17–6.96 (m, 12H), 6.89 (d, J = 2.0 Hz, 1H), 6.87 (d, J = 2.0 Hz, 1H), 6.82–6.74 (m, 4H), 6.72 (d, J = 2.1 Hz, 1H), 6.70 (d, J = 2.1 Hz, 1H), 6.59 (d, J = 2.1 Hz, 1H), 6.57 (d, J = 2.0 Hz, 1H), 4.24 (t, J = 5.6 Hz, 2H), 4.12 (t, J = 5.6 Hz, 2H), 3.76 (s, 6H), 3.09 (t, J = 5.5 Hz, 2H), 3.03 (t, J = 5.4 Hz, 2H), 2.88 (s, 8H), 2.11 (s, 3H), 2.07 (s, 3H), 1.95–1.89 (m, 8H). ^13^C-NMR (101 MHz, CDCl_3_) δ: 157.96, 157.89, 157.06, 142.06, 136.94, 136.12, 135.99, 135.94, 135.57, 132.22, 131.99, 131.43, 131.30, 131.25, 130.34, 130.31, 128.26, 127.61, 114.13, 113.56, 113.37, 113.33, 55.13, 54.78, 54.71, 54.64, 54.61, 23.44, 23.39, 23.35, 23.27. MS (ESI): *m/z* = 448.3 [M+H]^+^, *m/z* = 450.2 [M+H+2]^+^. R_f_: 0.5 (9:1 methylene chloride: methanol).

##### *E/Z*-1-(2-{4-[1-(4-Chloro-phenyl)-2-(4-methoxy-phenyl)-propenyl]-phenoxy}-ethyl)-piperidine (**12**)

C_29_H_32_ClNO_2_. Yield: 53%. Yellow oil. Purity: 100%. ^1^H-NMR (400 MHz, CDCl_3_) δ: 7.31 (d, J = 2.0 Hz, 1H), 7.29 (d, J = 2.1 Hz, 1H), 7.18–6.98 (m, 10H), 6.88 (d, J = 2.2 Hz, 1H), 6.87 (d, J = 2.1 Hz, 1H), 6.83–6.69 (m, 8H), 6.59 (d, J = 2.2 Hz, 1H), 6.57 (d, J = 2.1 Hz, 1H), 4.15 (t, J = 6.0 Hz, 2H), 4.03 (t, J = 6.0 Hz, 2H), 3.76 (s, 6H), 2.84 (t, J = 6.0 Hz, 2H), 2.76 (t, J = 6.0 Hz, 2H), 2.56 (d, J = 18.9 Hz, 8H), 2.11 (s, 3H), 2.08 (s, 2H), 1.64 (tt, J = 11.6, 5.6 Hz, 9H), 1.47 (dd, J = 13.2, 8.4 Hz, 4H). ^13^C-NMR (101 MHz, CDCl_3_) δ: 157.98, 157.89, 157.42, 156.73, 142.45, 142.12, 137.06, 136.97, 136.08, 136.00, 135.82, 135.49, 134.79, 132.21, 132.15, 131.91, 131.41, 131.30, 131.17, 130.33, 130.30, 128.25, 127.59, 114.15, 113.61, 113.38, 113.32, 65.65, 65.45, 57.86, 57.81, 55.12, 54.99, 54.95, 25.69, 25.62, 24.01, 23.95, 23.43, 23.25. MS (ESI): *m/z* = 462.3 [M+H]^+^ (100%), *m/z* = 464.2 [M+H+2]^+^ (33%). R_f_: 0.37 (93:7 methylene chloride: methanol).

##### *E/Z*-4-(2-{4-[1-(4-Chloro-phenyl)-2-(4-methoxy-phenyl)-propenyl]-phenoxy}-ethyl)-morpholine (**13**)

C_28_H_30_ClNO_3_. Yield: 45%. Dark orange oil. Purity: 97.45%. ^1^H-NMR (400 MHz, CDCl_3_) δ 7.31 (d, J = 1.7 Hz, 1H), 7.29 (d, J = 2.0 Hz, 1H), 7.17–6.97 (m, 10H), 6.89 (d, J = 2.8 Hz, 1H), 6.87 (d, J = 2.0 Hz, 1H), 6.82–6.74 (m, 4H), 6.73–6.68 (m, 4H), 6.59 (d, J = 1.9 Hz, 1H), 6.57 (d, J = 2.0 Hz, 1H), 4.14 (t, J = 5.7 Hz, 2H), 4.01 (t, J = 5.7 Hz, 2H), 3.83–3.69 (m, 14H), 2.83 (t, J = 5.6 Hz, 2H), 2.75 (t, J = 5.6 Hz, 2H), 2.59 (d, J = 20.5 Hz, 8H), 2.11 (s, 3H), 2.08 (s, 3H). ^13^C-NMR (101 MHz, CDCl_3_) δ: 157.97, 157.87, 157.36, 156.68, 142.42, 142.10, 136.99, 136.92, 136.07, 135.94, 135.92, 135.59, 135.52, 134.85, 132.22, 132.16, 131.95, 131.43, 131.31, 131.21, 130.34, 130.32, 128.26, 127.61, 114.12, 113.58, 113.37, 113.31, 66.86, 65.61, 65.42, 57.67, 57.65, 55.13, 54.06, 54.04, 53.43, 23.44, 23.28. MS (ESI): *m/z* = 464.3 [M+H]^+^ (100%), *m/z* = 466.2 [M+H+2]^+^ (33%). R_f_: 0.52 (95:5 methylene chloride: methanol).

##### *E/Z*-1-(2-{4-[1-(4-Chloro-phenyl)-2-(4-methoxy-phenyl)-propenyl]-phenoxy}-ethyl)-azepane (**14**)

C_30_H_34_ClNO_2_. Yield: 43%. Orange oil. Purity: 95%. ^1^H-NMR (400 MHz, CDCl_3_) δ 7.32–7.25 (m, 2H), 7.17–7.08 (m, 4H), 7.06–6.97 (m, 6H), 6.87 (d, J = 8.7 Hz, 2H), 6.81–6.74 (m, 4H), 6.70 (d, J = 8.7 Hz, 4H), 6.59–6.55 (m, 2H), 4.19 (t, J = 5.6 Hz, 2H), 4.07 (t, J = 5.7 Hz, 2H), 3.76 (s, 6H), 3.09 (t, J = 5.6 Hz, 2H), 3.04–3.00 (m, 2H), 2.97–2.84 (m, 8H), 2.10 (s, 3H), 2.07 (s, 3H), 1.81–1.69 (m, 8H), 1.68–1.60 (m, 8H). ^13^C-NMR (101 MHz, CDCl_3_) δ 157.94, 142.40, 142.07, 135.56, 134.91, 132.19, 131.96, 131.51–131.06, 130.31, 128.26, 127.60, 114.16, 113.60, 113.36, 56.25, 55.62, 55.12, 26.97, 23.34. MS (ESI): *m/z* = 476.4 [M+H]^+^ (100%), *m/z* = 478.4 [M+H+2]^+^ (33%). R_f_: 0.53 (9:1 methylene chloride: methanol).

##### *E/Z*-(3-{4-[1-(4-Chlorophenyl)-2-(3-fluoro-4-methoxyphenyl)-propenyl]-phenoxy}-propyl)-dimethyl-amine (**15**)

C_27_H_29_ClFNO_2_. Yield: 74%. Orange oil. Purity: 98.84%. ^1^H-NMR (400 MHz, CDCl_3_) δ 7.31–7.28 (m, 2H), 7.15–7.12 (m, 2H), 7.10–7.07 (m, 2H), 7.02–6.99 (m, 2H), 6.88 (s, 2H), 6.85 (d, J = 2.9 Hz, 2H), 6.82–6.79 (m, 3H), 6.77–6.73 (m, 5H), 6.58 (dd, J = 9.1, 2.3 Hz, 2H), 4.02 (t, J = 6.4 Hz, 2H), 3.91 (t, J = 6.4 Hz, 2H), 3.83 (s, 6H), 3.46–3.33 (m, 4H), 2.25 (dd, J = 12.1, 5.9 Hz, 10H), 2.10–2.04 (m, 12H). ^13^C-NMR: (101 MHz, CDCl_3_) δ 157.85, 157.27, 145.95 (d, J = 10.5 Hz), 145.92 (d, J = 10.5 Hz), 142.10, 141.75, 138.02, 137.95, 137.99, (d, J = 7.1 Hz), 135.18, 134.82, 134.29 (d, J = 1.4 Hz), 133.60 (d, J = 1.3 Hz), 132.32, 132.07, 131.78, 131.62, 131.30, 128.26, 127.71, 125.18 (d, J = 3.1 Hz), 125.08 (d, J = 3.1 Hz), 116.88 (d, J = 18.1 Hz), 116.83 (d, J = 18.3 Hz), 114.11, 113.66, 112.80 (d, J = 6.0 Hz), 112.78 (d, J = 5.9 Hz), 66.11, 65.93, 56.35 (d, J = 3.8 Hz), 56.10, 45.40, 45.35, 27.49, 27.41, 23.25, 23.06. MS (ESI): *m/z* = 454.30 [M+H]^+^, *m/z* = 456.30 [M+H+2]^+^ (100%). R_f_: 0.41 (93:7 methylene chloride: methanol).

##### *E/Z*-1-(2-{4-[1-(4-Chlorophenyl)-2-(3-fluoro-4-methoxyphenyl)-propenyl]-phenoxy}-ethyl)-pyrrolidine (**16**)

C_28_H_29_ClFNO_2_. Yield: 61%. Orange oil. Purity: 100%. ^1^H-NMR (500 MHz, CDCl_3_) δ 7.31–7.27 (m, 2H), 7.15–7.10 (m, 2H), 7.10–7.06 (m, 2H), 7.02–6.98 (m, 2H), 6.88 (d, J = 2.1 Hz, 1H), 6.86 (t, J = 2.6 Hz, 2H), 6.84 (dd, J = 5.9, 3.5 Hz, 1H), 6.81–6.79 (m, 2H), 6.78 (t, J = 4.8 Hz, 2H), 6.76–6.71 (m, 4H), 6.62–6.57 (m, 2H), 4.15 (t, J = 5.9 Hz, 2H), 4.04 (t, J = 5.9 Hz, 2H), 3.82 (s, 6H), 2.65 (d, J = 20.4 Hz, 8H), 2.67–2.64 (m, 8H), 2.10 (s, 3H), 2.07 (s, 3H), 1.90–1.76 (m, 8H). ^13^C-NMR: (126 MHz, CDCl_3_) δ 157.64, 157.06, 151.86 (d, J = 245.2 Hz), 151.84 (d, J = 244.9 Hz), 145.93 (t, J = 11.1 Hz), 142.08, 141.74, 138.00, 137.93, 136.87 (d, J = 6.4 Hz), 136.80 (d, J = 6.3 Hz), 135.38, 135.03, 134.36 (d, J = 1.3 Hz), 133.70 (d, J = 1.3 Hz), 132.36, 132.09, 131.80, 131.66, 131.32, 131.09, 128.30, 127.74, 125.20 (d, J = 3.3 Hz), 125.14 (d, J = 3.3 Hz), 116.87 (d, J = 18.4 Hz), 116.85 (d, J = 18.4 Hz), 114.19, 113.76, 112.80 (d, J = 5.9 Hz), 112.79 (d, J = 5.9 Hz), 66.82, 66.63, 56.12, 55.02 (d, J = 5.1 Hz), 54.70, 54.68, 23.48, 23.45, 23.28, 23.08. MS (ESI): *m/z* = 466.40 [M+H]^+^ (100%), *m/z* = 468.00 [M+H+2]^+^. R_f_: 0.34 (95:5 methylene chloride: methanol).

##### *E/Z*-1-(2-{4-[1-(4-Chlorophenyl)-2-(3-fluoro-4-methoxyphenyl)-propenyl]-phenoxy}-ethyl)-piperidine (**17**)

C_29_H_31_ClFNO_2_. Yield: 77%. Orange oil. Purity: 95.88%. ^1^H-NMR (400 MHz, CDCl_3_) δ 7.30–7.27 (m, 2H), 7.14–7.11 (m, 2H), 7.08 (d, J = 8.7 Hz, 2H), 7.02–6.98 (m, 2H), 6.88–6.84 (m, 4H), 6.82–6.77 (m, 4H), 6.76–6.73 (m, 4H), 6.60–6.56 (m, 2H), 4.12 (t, J = 6.0 Hz, 2H), 4.00 (t, J = 6.0 Hz, 2H), 3.82 (s, 6H), 2.79 (t, J = 6.0 Hz, 2H), 2.72 (t, J = 6.0 Hz, 2H), 2.58 (m, 8H), 2.07 (s, 3H), 2.05 (s, 3H), 1.60 (dd, J = 11.3, 5.6 Hz, 8H), 1.46–1.40 (m, 4H). ^13^C-NMR: (101 MHz, CDCl_3_) δ 157.65, 157.08, 151.87 (d, J = 245.2 Hz), 151.84 (d, J = 244.9 Hz), 145.97 (d, J = 10.8 Hz), 145.88 (d, J = 10.8 Hz), 142.08, 141.74, 138.01, 137.94, 136.88 (d, J = 8.5 Hz), 136.82 (d, J = 8.4 Hz), 135.34, 134.98, 134.35, 133.68, 132.36, 132.08, 131.79, 131.66, 131.31, 131.07, 128.30, 127.74, 125.19 (d, J = 3.4 Hz), 125.11 (d, J = 3.3 Hz), 116.88 (d, J = 18.4 Hz), 116.85 (d, J = 18.3 Hz) 114.20, 113.77, 112.82 (d, J = 5.0 Hz), 112.80 (d, J = 5.1 Hz), 65.72 (d, J = 17.0 Hz), 57.89 (d, J = 4.5 Hz), 56.12, 55.02, 54.99, 25.82, 25.76, 24.11, 24.07, 23.27, 23.08. MS (ESI): *m/z* = 480.01 [M+H]^+^, *m/z* = 482.01 [M+H+2]^+^ (100%). R_f_: 0.30 (94:6 methylene chloride: methanol).

##### *E/Z*-4-(2-{4-[1-(4-Chlorophenyl)-2-(3-fluoro-4-methoxyphenyl)-propenyl]-phenoxy}-ethyl)-morpholine (**18**)

C_28_H_29_ClFNO_3_. Yield: 65%. Brown oil. Purity: 100%. ^1^H-NMR (400 MHz, CDCl_3_) δ 7.32–7.25 (m, 3H), 7.16–7.07 (m, 4H), 7.03–6.99 (m, 2H), 6.90–6.73 (m, 11H), 6.62–6.57 (m, 2H), 4.11 (t, J = 5.6 Hz, 2H), 3.99–4.02 (t, J = 5.7 Hz, 2H), 3.84 (s, 6H), 3.77–3.70 (m, 8H), 2.80 (t, J = 5.6 Hz, 2H), 2.73 (t, J = 5.6 Hz, 2H), 2.64–2.50 (m, 8H), 2.09 (s, 3H), 2.06 (s, 3H).^13^C-NMR: (101 MHz, CDCl_3_) δ 157.51, 156.93, 151.82 (d, J = 245.2 Hz), 151.79 (d, J = 244.8 Hz), 145.87 (d, J = 10.3 Hz), 142.02, 141.69, 137.91, 137.85, 136.83 (d, J = 6.2 Hz), 136.71 (d, J = 6.2 Hz), 135.47, 135.11, 134.40 (d, J = 1.3 Hz), 133.76 (d, J = 1.3 Hz), 132.34, 132.04, 131.78, 131.63, 131.28, 131.07, 128.27, 127.71, 125.15 (d, J = 3.3 Hz), 125.04 (d, J = 3.4 Hz), 116.88 (d, J = 18.4 Hz), 116.80 (d, J = 18.5 Hz), 114.18, 113.75, 112.80 (d, J = 5.4 Hz), 112.78 (d, J = 5.5 Hz), 66.80, 66.76, 65.65, 65.48, 57.62, 57.58, 57.60 (d, J = 3.3 Hz), 54.03, 54.00, 53.51, 23.22, 23.04. MS (ESI): *m/z* = 482.00 [M+H]^+^ (100%), *m/z* = 484.00 [M+H+2]^+^. R_f_: 0.58 (95:5 methylene chloride: methanol).

##### *E/Z*-1-(2-{4-[1-(4-Chlorophenyl)-2-(3-fluoro-4-methoxyphenyl)-propenyl]-phenoxy}-ethyl)-azepane (**19**)

C_30_H_33_ClFNO_2_. Yield: 54%. Brown oil. Purity: 100%. ^1^H-NMR (400 MHz, CDCl_3_) δ 7.30–7.27 (m, 2H), 7.14–7.05 (m, 4H), 7.02–6.98 (m, 2H), 6.88–6.82 (m, 4H), 6.81–6.77 (m, 4H), 6.76–6.71 (m, 4H), 6.61 (dd, J = 6.8, 4.8 Hz, 2H), 4.18 (t, J = 6.0 Hz, 2H), 4.04 (t, J = 6.0 Hz, 2H), 3.83 (s, 6H), 3.03 (t, J = 6.0 Hz, 2H), 2.97 (t, J = 6.0 Hz, 2H), 2.90–2.81 (m, 8H), 2.08 (d, J = 14.2 Hz, 8H), 1.72 (d, J = 4.7 Hz, 6H), 1.65–1.60 (m, 8H). ^13^C-NMR: (101 MHz, CDCl_3_) δ 158.79 (d, J =157.7 Hz), 157.56, 151.02, 150.83, 145.97, 144.90, 141.71, 136.39 (d, J = 6.2 Hz), 136.26 (d, J = 6.2 Hz), 135.71, 135.43, 134.38 (d, J = 1.3 Hz), 134.27 (d, J = 1.3 Hz), 132.37, 132.07, 131.81, 131.66, 131.30, 131.09, 128.29, 127.73, 125.18 (d, J = 3.1 Hz), 125.09 (d, J = 3.1 Hz), 116.93 (d, J = 18.4 Hz), 116.74 (d, J = 18.4 Hz), 114.21, 113.76, 112.82 (d, J = 5.7 Hz), 112.79 (d, J = 5.7 Hz), 66.06, 56.37, 56.11, 55.77 (d, J = 5.87 Hz), 53.41, 27.11, 27.01, 26.99, 26.90, 23.26, 23.07. MS (ESI): *m/z* = 494.04 [M+H]^+^ (100%), *m/z* = 496.04 [M+H+2]^+^. R_f_: 0.34 (93:7 methylene chloride: methanol).

##### *E/Z*-(2-{4-[1-(4-Chlorophenyl)-2-(3-fluoro-4-methoxyphenyl)-propenyl]-phenoxy}-ethyl)-dimethyl-amine (**20**)

C_26_H_27_ClFNO_2_. Yield: 55%. Reddish-brown oil. Purity: 98.69%. ^1^H-NMR (400 MHz, CDCl_3_) δ 7.32–7.28 (m, 2H), 7.16–7.12 (m, 2H), 7.11–7.07 (m, 2H), 7.03–6.99 (m, 2H), 6.91–6.87 (m, 3H), 6.86 (d, J = 1.9 Hz, 1H), 6.82 (d, J = 1.9 Hz, 1H), 6.79 (dd, J = 5.7, 2.9 Hz, 2H), 6.78–6.74 (m, 4H), 6.72 (dd, J = 8.5, 1.5 Hz, 1H), 6.64–6.59 (m, 2H), 4.10 (t, J = 5.7 Hz, 2H), 3.98 (t, J = 5.7 Hz, 2H), 3.84 (s, 6H), 2.77 (t, J = 5.7 Hz, 2H), 2.70 (t, J = 5.7 Hz, 2H), 2.37 (s, 6H), 2.33 (s, 6H), 2.09 (s, 3H), 2.06 (s, 3H). ^13^C-NMR: (101 MHz, CDCl_3_) δ 157.65, 157.07, 151.97 (d, J = 245.1 Hz), 151.94 (d, J = 244.9 Hz), 145.99 (d, J = 9.6 Hz), 142.07, 141.73, 138.01, 137.95, 136.81 (d, J = 6.7 Hz), 136.77 (d, J = 6.7 Hz),135.42, 135.07, 134.37 (d, J = 1.3 Hz), 133.72 (d, J = 1.6 Hz), 132.38, 132.09, 131.79, 131.68, 131.32, 131.09, 128.31, 127.76, 125.20 (d, J = 3.4 Hz), 125.13 (d, J = 3.4 Hz), 116.86 (d, J = 18.4 Hz), 116.98 (d, J = 18.3 Hz), 114.19, 113.75, 112.82 (d, J = 5.1 Hz), 112.80 (d, J = 5.2 Hz), 65.85, 65.64, 58.27, 58.22, 56.14, 45.83, 45.80, 23.29, 23.07. MS (ESI): *m/z* = 440.30 [M+H]^+^ (100%), *m/z* = 442.30 [M+2]^+^. R_f_: 0.38 (92:8 methylene chloride: methanol).

##### *E/Z*-(2-{4-[1-(4-Chlorophenyl)-2-(3-fluoro-4-methoxyphenyl)-propenyl]-phenoxy}-ethyl)-diethyl-amine (**21**)

C_28_H_31_ClFNO_2_. Yield: 51%. Yellow oil. Purity: 98.96%. ^1^H-NMR (500 MHz, CDCl_3_) δ 7.30 (d, J = 8.4 Hz, 2H), 7.14 (d, J = 8.4 Hz, 2H), 7.09 (d, J = 8.6 Hz, 2H), 7.02 (d, J = 8.5 Hz, 2H), 6.87 (dd, J = 11.8, 2.6 Hz, 4H), 6.80 (t, J = 8.0 Hz, 3H), 6.78–6.70 (m, 5H), 6.59 (d, J = 8.7 Hz, 2H), 4.10 (d, J = 5.4 Hz, 2H), 4.00 (s, 2H), 3.84 (s, 6H), 2.95 (d, J = 4.8 Hz, 2H), 2.88 (s, 2H), 2.78–2.57 (m, 8H), 2.09 (d, J = 7.5 Hz, 3H), 2.09 (d, J = 7.5 Hz, 3H), 1.14–1.04 (m, 12H). ^13^C-NMR: (126 MHz, CDCl_3_) δ 157.58, 156.96, 151.89 (d, J = 245.2 Hz), 151.85 (d, J = 244.8 Hz), 145.94 (t, J = 11.3 Hz), 142.07, 141.73, 137.99, 137.93, 136.89 (d, J = 6.2 Hz), 136.81 (d, J = 6.4 Hz), 135.43, 135.09, 134.38 (d, J = 1.1 Hz), 133.74 (d, J = 1.1 Hz), 132.39, 132.10, 131.84, 131.69, 131.33, 131.12, 128.32, 127.77, 125.21 (d, J = 3.3 Hz), 125.13 (d, J = 3.4 Hz), 116.91 (d, J = 18.4 Hz), 116.86 (d, J = 18.4 Hz), 114.15, 113.71, 112.81 (d, J = 5.9 Hz), 112.79 (d, J = 5.9 Hz), 66.12, 56.14, 53.42, 51.67, 51.54, 47.80, 47.77, 23.30, 23.09, 11.56. MS (ESI): *m/z* = 468.30 [M+H]^+^ (100%), *m/z* = 470.00 [M+H+2]^+^. R_f_: 0.32 (93:7 methylene chloride: methanol).

##### *E/Z*-(3-{4-[1-(4-Chlorophenyl)-2-(4-fluoro-3-methoxyphenyl)-propenyl]-phenoxy}-propyl)-dimethyl-amine (**22**)

C_27_H_29_ClFNO_2_. Yield: 50%. Orange oil. Purity: 97.83%. ^1^H-NMR (500 MHz, CDCl_3_) δ 7.31 (d, J = 8.4 Hz, 1H), 7.16 (d, J = 8.3 Hz, 1H), 7.10 (t, J = 5.7 Hz, 3H), 7.02 (d, J = 8.4 Hz, 3H), 6.92–6.85 (m, 5H), 6.81 (t, J = 7.1 Hz, 3H), 6.75 (d, J = 8.7 Hz, 1H), 6.72–6.67 (m, 2H), 6.65 (dd, J = 8.4, 1.9 Hz, 1H), 6.63 (dd, J = 8.3, 1.9 Hz, 1H), 6.59 (d, J = 8.7 Hz, 1H), 4.04 (t, J = 6.3 Hz, 2H), 3.91 (t, J = 6.3 Hz, 2H), 3.62 (d, J = 2.0 Hz, 6H), 2.56 (dt, J = 27.7, 7.4 Hz, 4H), 2.37–2.31 (m, 12H), 2.11 (d, J = 17.4 Hz, 6H), 2.07–1.94 (m, 4H). ^13^C-NMR: (126 MHz, CDCl_3_) δ 157.79, 157.21, 150.97 (d, J = 245.6 Hz), 150.89 (d, J = 245.3 Hz), 146.89 (d, J = 10.8 Hz), 146.82 (d, J = 10.7 Hz), 141.93, 141.88, 140.08 (d, J = 4.0 Hz), 139.98 (d, J = 4.0 Hz), 138.09, 138.07, 135.19, 135.11, 135.02, 134.44, 132.44, 132.01, 131.75, 131.73, 131.33, 131.12, 128.34, 127.75, 121.42 (d, J = 6.6 Hz), 115.54 (d, J = 18.2 Hz), 115.43 (d, J = 18.2 Hz), 115.09, 114.13, 113.67, 65.94, 65.80, 56.37, 56.31, 56.03 (d, J = 2.6 Hz), 53.43, 45.17, 45.10, 27.16, 27.04, 23.24, 23.05.MS (ESI): *m/z* = 454.30 [M+H]^+^ (100%), *m/z* = 456.30 [M++H+2]^+^. R_f_: 0.33 (91:9 methylene chloride: methanol).

##### *E/Z*-1-(2-{4-[1-(4-Chlorophenyl)-2-(4-fluoro-3-methoxyphenyl)-propenyl]-phenoxy}-ethyl)-pyrrolidine (**23**)

C_28_H_29_ClFNO_2_. Yield: 71%. Brown oil. Purity: 100%. ^1^H-NMR (400 MHz, CDCl_3_) δ 7.31 (d, J = 8.4 Hz, 2H), 7.16 (d, J = 8.4 Hz, 2H), 7.11 (d, J = 8.6 Hz, 2H), 7.02 (d, J = 8.4 Hz, 2H), 6.89 (dt, J = 8.2, 5.5 Hz, 4H), 6.81 (d, J = 8.4 Hz, 2H), 6.76 (d, J = 8.7 Hz, 2H), 6.72–6.67 (m, 3H), 6.64 (t, J = 2.0 Hz, 1H), 6.61 (d, J = 8.6 Hz, 2H), 4.18 (t, J = 5.8 Hz, 2H), 4.05 (t, J = 5.8 Hz, 2H), 3.62 (d, J = 2.8 Hz, 6H), 2.98 (t, J = 5.8 Hz, 2H), 2.90 (t, J = 5.8 Hz, 2H), 2.71 (d, J = 21.8 Hz, 8H), 2.13 (s, 3H), 2.09 (s, 3H), 1.89–1.80 (m, 8H). ^13^C-NMR: (101 MHz, CDCl_3_) δ 157.58, 157.01, 152.21, 152.16, 146.90 (d, J = 9.9 Hz), 146.89 (d, J = 9.8 Hz), 141.91, 141.86, 140.03 (d, J = 3.9 Hz), 139.97 (d, J = 4.1 Hz), 138.06, 138.03, 135.34, 135.25, 135.06, 134.47, 132.44, 132.00, 131.73, 131.31, 131.11, 128.33, 127.75, 121.45 (d, J = 6.0 Hz), 115.57 (d, J = 18.2 Hz), 115.39, (d, J = 18.3 Hz), 115.12 (d, J = 2.0 Hz), 115.11, 114.23, 113.79, 66.63, 66.50, 56.03 (d, J = 1.8 Hz), 54.99, 54.91, 54.69, 54.65, 23.48, 23.44, 23.22, 23.03. MS (ESI): *m/z* = 466.30 [M+H]^+^ (100%), *m/z* = 468.20 [M+H+2]^+^. R_f_: 0.32 (94:6 methylene chloride: methanol).

##### *E/Z*-1-(2-{4-[1-(4-Chlorophenyl)-2-(4-fluoro-3-methoxyphenyl)-propenyl]-phenoxy}-ethyl)-piperidine (**24**)

C_29_H_31_ClFNO_2_. Yield: 42%. Orange oil. Purity: 95%. ^1^H-NMR (400 MHz, CDCl_3_)) δ 7.33 (d, J = 7.5 Hz, 2H), 7.16 (dd, J = 17.6, 7.6 Hz, 4H), 7.04 (d, J = 7.6 Hz, 2H), 6.91 (t, J = 9.5 Hz, 4H), 6.83 (d, J = 7.4 Hz, 2H), 6.77 (d, J = 7.7 Hz, 2H), 6.70 (d, J = 13.9 Hz, 3H), 6.62 (d, J = 8.6 Hz, 3H), 4.23 (s, 2H), 4.10 (s, 2H), 3.66 (s, 6H), 2.91 (dd, J = 30.7, 19.6 Hz, 4H), 2.63 (s, 6H), 2.13 (d, J = 13.6 Hz, 6H), 1.71 (s, 8H), 1.47 (d, J = 23.0 Hz, 6H). ^13^C-NMR: (101 MHz, CDCl_3_) δ 157.79, 157.21, 150.97, 150.89, 146.90 (d, J = 9.2 Hz), 146.81 (d, J = 9.0 Hz), 141.93, 141.88, 140.08 (d, J = 4.0 Hz), 139.98 (d, J = 4.0 Hz), 138.09, 138.07, 135.19, 135.11, 135.02, 134.44, 132.44, 132.01, 131.75, 131.73, 131.33, 131.12, 128.34, 127.75, 121.42 (t, J = 6.6 Hz), 115.56 (d, J = 13.4 Hz), 115.41 (d, J = 13.3 Hz), 115.09,114.33, 114.13, 113.67, 66.22, 57.98, 57.67, 56.04, 54.85 (d, J = 6.4 Hz), 53.40, 26.91, 25.63, 25.29, 23.72, 23.22, 23.04. MS (ESI): *m/z* = 480.01 [M+H]^+^ (100%), *m/z* = 482.01 [M+H+2]^+^. R_f_: 0.40 (95:5 methylene chloride: methanol).

##### *E/Z*-4-(2-{4-[1-(4-Chlorophenyl)-2-(4-fluoro-3-methoxyphenyl)-propenyl]-phenoxy}-ethyl)-morpholine (**25**)

C_28_H_29_ClFNO_3_. Yield: 61%. Orange oil. Purity: 97.07%. ^1^H-NMR (400 MHz, CDCl_3_) δ 7.31 (d, J = 8.4 Hz, 2H), 7.14 (dd, J = 15.7, 8.5 Hz, 4H), 7.02 (d, J = 8.4 Hz, 2H), 6.90 (t, J = 9.1 Hz, 4H), 6.81 (d, J = 8.4 Hz, 2H), 6.76 (d, J = 8.7 Hz, 2H), 6.73–6.65 (m, 3H), 6.64 (dd, = 5.4, 3.3 Hz, 1H), 6.61 (t, J = 6.2 Hz, 2H), 4.15 (t, J = 5.5 Hz, 2H), 4.03 (t, J = 5.5 Hz, 2H), 3.80–3.70 (m, 8H), 3.63 (s, 6H), 2.86 (t, J = 5.3 Hz, 2H), 2.77 (t, J = 5.3 Hz, 2H), 2.61 (d, J = 21.5 Hz, 8H), 2.13 (s, 3H), 2.10 (s, 3H). ^13^C-NMR: (101 MHz, CDCl_3_) δ 157.67, 157.09, 151.14 (d, J = 245.8 Hz), 151.06 (d, J = 245.2 Hz), 147.06 (d, J = 10.8 Hz), 146.99 (d, J = 10.8 Hz), 142.02, 141.98, 140.19 (d, J = 4.1 Hz), 140.06 (d, J = 4.0 Hz), 138.16, 138.14, 135.58, 135.49, 135.25, 134.70, 132.61, 132.13, 131.90, 131.44, 131.28, 128.49, 127.91, 121.57 (d, J = 5.1 Hz), 121.51 (d, J = 5.2 Hz), 115.69 (d, J = 18.2 Hz), 115.59 (d, J = 18.3 Hz), 115.27 (d, J = 1.4 Hz), 115.22 (d, J = 1.8 Hz), 115.12, 114.37, 113.93, 66.89, 65.70, 65.63, 57.76, 56.19, 54.19, 54.15, 53.56, 23.36, 23.19. MS (ESI): *m/z* = 482.30 [M+H]^+^ (100%), *m/z* = 484.20 [M+H+2]^+^. R_f_: 0.35 (94:6 methylene chloride: methanol).

##### *E/Z*-1-(2-{4-[1-(4-Chlorophenyl)-2-(4-fluoro-3-methoxyphenyl)-propenyl]-phenoxy}-ethyl)-azepane (**26**)

C_30_H_33_ClFNO_2_. Yield: 62%. Orange oil. Purity: 95%. ^1^H-NMR (400 MHz, CDCl_3_) δ 7.31 (d, J = 8.4 Hz, 2H), 7.16 (d, J = 8.4 Hz, 2H), 7.11 (d, J = 8.6 Hz, 2H), 7.02 (d, J = 8.5 Hz, 2H), 6.93–6.86 (m, 4H), 6.81 (d, J = 8.4 Hz, 2H), 6.76 (d, J = 8.7 Hz, 2H), 6.73–6.66 (m, 3H), 6.64 (s, 1H), 6.61 (t, J = 6.3 Hz, 2H), 4.13 (t, J = 6.0 Hz, 2H), 4.00 (t, J = 6.0 Hz, 2H), 3.62 (d, J = 3.2 Hz, 6H), 3.01 (t, J = 6.0 Hz, 2H), 2.93 (t, J = 6.0 Hz, 2H), 2.87–2.76 (m, 8H), 2.13 (s, 3H), 2.10 (s, 3H), 1.74–1.65 (m, 8H), 1.64–1.58 (m, 8H). ^13^C-NMR: (101 MHz, CDCl_3_) δ 157.68, 157.10, 150.98 (d, J = 245.8 Hz), 150.91 (d, J = 245.4 Hz), 146.90 (d, J = 10.6 Hz), 146.83 (d, J = 10.8 Hz), 141.93, 141.87, 140.05 (d, J = 4.0 Hz), 139.97 (d, J = 4.0 Hz), 138.08, 138.06, 135.26, 135.17, 135.04, 134.45, 132.44, 132.00, 131.73, 131.31, 131.10, 128.33, 127.75, 121.42 (d, J = 6.9 Hz), 121.31, 115.53 (d, J = 18.4 Hz), 115.43 (d, J = 18.3 Hz), 115.12, 114.24, 113.80,66.16, 66.00, 56.40, 56.30, 56.03 (d, J = 2.3 Hz), 55.83, 55.77, 27.51, 27.47, 27.04, 27.02, 23.23, 23.03. MS (ESI): *m/z* = 494.04 [M+H]^+^ (100%), *m/z* = 496.04 [M+2]^+^. R_f_: 0.50 (95:5 methylene chloride: methanol).

##### *E/Z*-(2-{4-[1-(4-Chlorophenyl)-2-(4-fluoro-3-methoxyphenyl)-propenyl]-phenoxy}-ethyl)-dimethyl-amine (**27**)

C_26_H_27_ClFNO_2_. Yield: 67%. Yellow oil. Purity: 100%. ^1^H-NMR (400 MHz, CDCl_3_) δ 7.31 (d, J = 8.4 Hz, 2H), 7.16 (d, J = 8.4 Hz, 2H), 7.11 (d, J = 8.6 Hz, 2H), 7.02 (d, J = 8.5 Hz, 2H), 6.92–6.86 (m, 4H), 6.81 (d, J = 8.5 Hz, 2H), 6.76 (d, J = 8.7 Hz, 2H), 6.73–6.65 (m, 3H), 6.62 (t, J = 7.5 Hz, 3H), 4.11 (t, J = 5.7 Hz, 2H), 3.98 (t, J = 5.7 Hz, 2H), 3.62 (d, J = 4.2 Hz, 6H), 2.78 (t, J = 5.7 Hz, 2H), 2.70 (t, J = 5.7 Hz, 2H), 2.38 (s, 6H), 2.33 (s, 6H), 2.13 (s, 3H), 2.09 (s, 3H). ^13^C-NMR: (101 MHz, CDCl_3_) δ 157.14, 156.57, 150.46 (d, J = 245.5 Hz), 150.38 (d, J = 245.3 Hz), 146.37 (d, J = 10.8 Hz), 146.31 (d, J = 10.8 Hz), 141.40, 141.34, 139.51 (d, J = 4.2 Hz), 139.45 (d, J = 4.0 Hz), 137.55, 137.52, 134.77, 134.68, 134.52, 133.93, 131.91, 131.48, 131.19, 130.79, 130.57, 127.81, 127.22, 120.90 (d, J = 6.8 Hz), 120.81 (d, J = 6.7 Hz), 115.04 (d, J = 10.6 Hz), 114.86, 114.81, 114.60, 113.69, 113.25, 65.83, 65.68, 58.25, 58.18, 56.03 (d, J = 3.1 Hz), 56.01, 45.82, 45.76, 23.23, 23.02. MS (ESI): *m/z* = 440.30 [M+H]^+^ (100%), *m/z* = 442.30 [M+H+2]^+^. R_f_: 0.33 (94:6 methylene chloride: methanol).

##### *E/Z*-(2-{4-[1-(4-Chlorophenyl)-2-(4-fluoro-3-methoxyphenyl)-propenyl]-phenoxy}-ethyl)-diethyl-amine (**28**)

C_28_H_31_ClFNO_2_. Yield: 52%. Orange oil. Purity: 97.68%. ^1^H-NMR ((400 MHz, CDCl_3_) δ 7.31 (d, J = 7.0 Hz, 2H), 7.14 (dd, J = 16.5, 7.5 Hz, 4H), 7.02 (d, J = 7.2 Hz, 2H), 6.89 (t, J = 8.9 Hz, 4H), 6.81 (d, J = 7.5 Hz, 2H), 6.76 (d, J = 7.4 Hz, 2H), 6.73–6.65 (m, 3H), 6.65–6.57 (m, 3H), 4.17 (s, 2H), 4.04 (s, 2H), 3.63 (s, 6H), 2.97 (d, J = 30.6 Hz, 4H), 2.76 (d, J = 12.1 Hz, 8H), 2.13 (s, 3H), 2.10 (s, 3H), 1.19–1.08 (m, 12H). ^13^C-NMR: (101 MHz, CDCl_3_) δ 158.81, 158.37, 151.95, 151.76, 145.82 (d, J = 9.7 Hz), 145.80 (d, J = 9.6 Hz), 142.03, 141.98, 140.95, 140.69, 138.17, 135.25, 134.70, 134.56, 133.47, 132.62, 132.14, 131.92, 131.46, 131.29, 128.49, 127.91, 121.36 (d, J = 6.6 Hz), 117.43 (d, J = 13.6 Hz), 115.26 (d, J = 13.6 Hz), 115.23, 114.34, 113.88, 56.20, 53.56, 53.38, 51.73, 51.59, 47.87, 47.83, 23.38, 23.20, 11.39, 11.31. MS (ESI): *m/z* = 468.30 [M+H]^+^ (100%), *m/z* = 470.30 [M+H+2]^+^. R_f_: 0.50 (93:7 methylene chloride: methanol).

### 3.2. Biology

#### 3.2.1. Yeast Estrogen Receptor Assay (YES)

The yeast estrogen receptor assay was supplied by Dr. J.P. Sumpter (Brunel University, Uxbridge, UK) and was used to determine the relative transactivation activity of the human ERα as formerly described [15]. Briefly, *Saccharomyces cerevisiae* stably transfected with a human ERα and an estrogen-responsive element fused to the reporter gene *lacZ* encoding for β-galactosidase were treated with the test substances for about 48 h. The β-galactosidase enzymatic activity was measured in a colorimetric assay using a microplate photometer by hydrolysis of the substrate chlorophenol red β-D-galactopyranoside (Roche Diagnostics, Mannheim, Germany), which leads to the formation of chlorophenol red. This can be measured as an increased absorption at 540 nm. All compounds were diluted in DMSO. 17β-estradiol (E2) (Sigma, Deissenhofen, Germany) 10 nM was used as a positive control, and DMSO was used as vehicle control. All compounds, also TAM (TAM) (Biotrend, Cologne, Germany), and 4-hydroxy-TAM (4-OH-TAM), were screened for agonistic and anti-estrogenic activity in a concentration of 1 μM; anti-estrogenic assays were performed in combination with 0.5 nM/1 nM E2 depending on the EC_50_ value in each experimental series. All compounds were tested in technical quadruplicates and biological triplicates. Statistical analysis was performed by analysis of variance (ANOVA) and Tukey’s post-hoc test with the significance level of *p* < 0.05. The relative β-galactosidase activity of all compounds is shown in Table 2, Table 3 and Table 4.

#### 3.2.2. NCI Anti-Cancer Screening

All compounds were subjected to the NCI in vitro disease-oriented human cells screening panel assay. The human tumor cell lines of the cancer-screening panel are grown in RPMI 1640 medium containing 5% fetal bovine serum and 2 mM L-glutamine. For a typical screening experiment, cells are inoculated into 96-well microtiter plates in 100 μL at plating densities ranging from 5000 to 40,000 cells/well depending on the doubling time of individual cell lines. After cell inoculation, the microtiter plates are incubated at 37 °C, 5% CO_2_, 95% air, and 100% relative humidity for 24 h prior to the addition of experimental drugs. After 24 h, two plates of each cell line are fixed in situ with TCA to represent a measurement of the cell population for each cell line at the time of drug addition. Experimental drugs are solubilized in dimethyl sulfoxide at 400-fold the desired final maximum test concentration and stored frozen prior to use. At the time of drug addition, an aliquot of frozen concentrate is thawed and diluted to twice the desired final maximum test concentration with complete medium containing 50 μg/mL gentamicin. Additional four, 10-fold, or ½ log serial dilutions are made to provide a total of five drug concentrations plus control. Aliquots of 100 μL of these different drug dilutions are added to the appropriate microtiter wells already containing 100 μL of medium, resulting in the required final drug concentrations. Following drug addition, the plates are incubated for an additional 48 h at 37 °C, 5% CO_2_, 95% air, and 100% relative humidity. For adherent cells, the assay is terminated by the addition of cold TCA. Cells are fixed in situ by the gentle addition of 50 μL of cold 50% (*w/v*) TCA (final concentration, 10% TCA) and incubated for 60 min at 4 °C. The supernatant is discarded, and the plates are washed five times with tap water and air-dried. Sulforhodamine B (SRB) solution (100 μL) at 0.4% (*w/v*) in 1% acetic acid is added to each well, and plates are incubated for 10 min at room temperature. After staining, unbound dye is removed by washing five times with 1% acetic acid, and the plates are air-dried. Bound stain is subsequently solubilized with a 10 mM trizma base, and the absorbance is read on an automated plate reader at a wavelength of 515 nm. For suspension cells, the methodology is the same except that the assay is terminated by fixing settled cells at the bottom of the wells by gently adding 50 μL of 80% TCA (final concentration, 16% TCA). Compounds are screened at a dose of 10 µM, hits showing mean growth inhibition over 60 cell lines >50% are escalated for 5-dose screening assay. To construct a dose-response curve, about 60 cell lines of nine tumor subpanels were incubated with five concentrations (0.01–100 μM) for each compound. Three response parameters (GI_50_, TGI, and LC_50_) were calculated for each cell line. The GI_50_ value corresponds to the compound’s concentration causing a 50% decrease in net cell growth, the TGI value is the compound’s concentration resulting in total growth inhibition, and the LC_50_ value is the compound’s concentration causing a net 50% loss of initial cells at the end of the incubation period (48 h) [41].

#### 3.2.3. Alkaline Phosphatase Activity in Ishikawa Cells

Estrogens stimulate the activity of alkaline phosphatase (AlkP) in Ishikawa cells (human endometrial adenocarcinoma cells; kindly provided by Prof. Masato Nishida, National Hospital Organization, Kasumigaura Medical Center, Japan). This enzyme activity is estimated by using the chromogen substrate (4-nitrophenylphosphate). These cells are very sensitive to estrogens; estradiol already induces the AlkP activity at a concentration of 10*^−^*^12^ M [20]. The procedure was modified by Littlefield et al., 1990 [42].

Briefly, cells were cultured in DMEM/F12 medium without phenol red containing 5% dextran-coated charcoal-treated FCS (DCC, BioWest, (Nuaille, France) and insulin–transferrin–selenium A (Invitrogen, Karlsruhe, Germany). Cells were kept in plastic culture flasks at 5% CO_2_ and 37 °C and harvested by brief exposure to trypsin (0.05%) EDTA at 37 °C. For experiments, the cells were seeded in 96-well plates at the required density of 11,000 cells per well. Compounds, diluted in DMSO (Carl Roth GmbH, Germany), were tested in a concentration of 1 µM. DMSO was used as a negative control, 10 nM 17β-estradiol as a positive control, respectively. After 72 h incubation, cells were harvested, washed twice with PBS, and incubated at −80 °C for about 30 min to lyse the cells. After thawing, the lysates were resuspended in reaction buffer (274 mM mannitol, 100 mM CAPS, 4 mM MgCl_2_, pH 10.4) containing 4 mM p-nitrophenylphosphate (NPP). After incubation for 1 h in the dark, AlkP activity was assayed by using the hydrolysis of p-nitrophenylphosphate to p-nitrophenol at pH 10.4 and the spectrometric determination of the kinetic of the product formation at 405 nm. All compounds were tested in technical triplicates and biological triplicates. Statistical analysis was performed by analysis of variance (ANOVA) and Tukey’s post-hoc test with the significance level of *p* < 0.05.

#### 3.2.4. Uterotrophic Assay

The most common short-term in vivo assay for (anti)-estrogenicity is the uterine growth test, suitable for screening ERα agonists and antagonists. The primary endpoint is the uterine wet weight (UWW). An increase in UWW indicates an estrogenic activity of the test compound [43]. Sprague Dawley female rats (170–200 g) were obtained from the animal colony of the National Institute of Research (Cairo, Egypt). The rats were housed in a temperature-controlled room (23–24 °C) with a 12 h light:dark cycle and with free access to food and water. They were allowed to acclimatize to the animal house of the German University in Cairo for at least 1 week before initiating the experiments. All efforts were made to minimize animal discomfort and suffering. Animals were ovariectomized. After 14 days of endogenous hormonal decline, the animals were subcutaneously treated for three days with respective compounds. The animals were randomly allocated to treatment and vehicle groups (n = 6). 17β-estradiol were administrated s.c. at a dose of 10 µg/kg/d BW, all test compounds at a dose of 10 mg/kg/d BW daily for a period of three days. Animals were sacrificed by CO_2_ inhalation after light anesthesia by inhaling an O_2_/CO_2_ mixture around 24 h after the third administration. The uterus wet weight was determined.

### 3.3. In Silico Study

A docking experiment was implemented to dock compounds **3** into the active site of estrogen receptor α (ERα) with the program MOE version 2009.10. The Protein Data Bank (PDB) crystal structure of ERα co-crystallized with DES (3ERD) was imported into MOE [40]. All possible hydrogen atoms were added. Atomic charges were assigned using the MMFF94 force field parameters in MOE. The binding pocket was selected and extended 4.5 Å around the pocket. Compound **3E**, **3Z,** and DES were built using MOE builder; we run a conformational search to build a database (.mdb) of the most stable conformers of the three compounds. The.mdb file was then docked into the pocket, the poses from the ligand conformation were generated using alpha triangle, the scoring function used was London dG with no refinement. To ensure more accurate docking procedures, DES were redocked to the binding pocket using the same MOE settings as compound **3**. MOE was also used to represent the 2D interactions within ERα LBD.

## 4. Conclusions

Structural modifications on rings **A**, **B,** and **C** of TAM led to compounds with moderate to high estrogenic activity and potential growth inhibition activity on ER-positive and -negative breast cancer cell lines. Compounds **12** and **19** were tested in vivo in an ovariectomized rat model and are promising candidates for the development of novel SERMs with potent anti-neoplastic activity. This work opens the horizon for further development of triphenylethylenes where the para position of ring **C** bears different substituents; such structural modification can alter both their estrogenic/anti-estrogenic properties and have a prominent effect on their metabolic fate.

## Figures and Tables

**Table 1:**
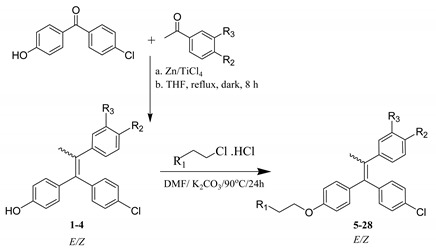
Synthetic scheme: Preparation of compounds **1**–**28**.

Code	R1	R2	R3
**1**	---	H	H
**2**	---	OCH_3_	H
**3**	---	OCH_3_	F
**4**	---	F	OCH_3_
**5**	-CH_2_-N-(CH_3_)_2_	H	H
**6**	-N-CH_2_-CH_2_-CH_2_-CH_2_-	H	H
**7**	-N-CH_2_-CH_2_-CH_2_-CH_2_-CH_2_-	H	H
**8**	-N-CH_2_-CH_2_-O-CH_2_-CH_2_-	H	H
**9**	-N-CH_2_-CH_2_-CH_2_-CH_2_-CH_2_-CH_2_-	H	H
**10**	-CH_2_-N-(CH_3_)_2_	OCH_3_	H
**11**	-N-CH_2_-CH_2_-CH_2_-CH_2_-	OCH_3_	H
**12**	-N-CH_2_-CH_2_-CH_2_-CH_2_-CH_2_-	OCH_3_	H
**13**	-N-CH_2_-CH_2_-O-CH_2_-CH_2_-	OCH_3_	H
**14**	-N-CH_2_-CH_2_-CH_2_-CH_2_-CH_2_-CH_2_-	OCH_3_	H
**15**	-CH_2_-N-(CH_3_)_2_	OCH_3_	F
**16**	-N-CH_2_-CH_2_-CH_2_-CH_2_-	OCH_3_	F
**17**	-N-CH_2_-CH_2_-CH_2_-CH_2_-CH_2_-	OCH_3_	F
**18**	-N-CH_2_-CH_2_-O-CH_2_-CH_2_-	OCH_3_	F
**19**	-N-CH_2_-CH_2_-CH_2_-CH_2_-CH_2_-CH_2_-	OCH_3_	F
**20**	-N-(CH_3_)_2_	OCH_3_	F
**21**	-N-(C_2_H_5_)_2_	OCH_3_	F
**22**	-CH_2_-N-(CH_3_)_2_	F	OCH_3_
**23**	-N-CH_2_-CH_2_-CH_2_-CH_2_-	F	OCH_3_
**24**	-N-CH_2-_CH_2_-CH_2_-CH_2_-CH_2_-	F	OCH_3_
**25**	-N-CH_2_-CH_2_-O-CH_2_-CH_2_-	F	OCH_3_
**26**	-N-CH_2-_CH_2_-CH_2_-CH_2_-CH_2_-CH_2_-	F	OCH_3_
**27**	-N-(CH_3_)_2_	F	OCH_3_
**28**	-N-(C2H_5_)_2_	F	OCH_3_

**Table 2:** Relative β-galactosidase activity using YES assay (antagonistic activity).

Code	Anti-Estrogenic Activity *	Code	Anti-Estrogenic Activity *
**5**	1.34 ± 0.15	**18**	n.d.
**6**	1.20 ± 0.19	**19**	n.d.
**7**	1.35 ± 0.16	**20**	n.d.
**8**	1.11 ± 0.26	**21**	n.d.
**9**	1.99 ± 0.02	**22**	0.97 ± 0.03
**10**	3.92 ± 0.58	**23**	1.06 ± 0.05
**11**	1.55 ± 0.12	**24**	1.19 ± 0.10
**12**	2.55 ± 0.41	**25**	1.18 ± 0.03
**13**	n.d. **	**26**	1.05 ± 0.12
**14**	1.53 ± 0.09	**27**	0.86 ± 0.04
**15**	n.d.	**28**	0.86 ± 0.07
**16**	n.d.	**TAM**	0.30 ± 0.08
**17**	n.d.	**4-OH-TAM**	0.21 ± 0.004

* Relative anti-estrogenic activity is compared to 0.5 nM/1 nM E2 (set as 1), compounds screened at a dose of 1 µM in presence of 0.5 nM/1 nM E2, respectively; compounds were screened in triplicates; ** n.d. = not determined. Compounds were not selected for anti-estrogenic assays due to their high estrogenic activity.

**Table 3:** Relative β-galactosidase activity using YES assay (agonistic activity).

Code	Estrogenic Activity *	Code	Estrogenic Activity *
**3**	12.83 ± 2.72	**18**	1.76 ± 0.56
**4**	6.62 ± 1.74	**19**	3.22 ± 1.22
**5**	2.25 ± 0.92	**20**	2.40 ± 0.64
**6**	2.88 ± 0.89	**21**	2.40 ± 0.77
**7**	2.87 ± 0.90	**22**	1.87 ± 0.74
**8**	1.85 ± 0.11	**23**	1.41 ± 1.01
**9**	6.74 ± 1.67	**24**	1.19 ± 0.20
**10**	11.61 ± 0.99	**25**	1.31 ± 0.30
**11**	1.98 ± 0.12	**26**	1.78 ± 0.64
**12**	3.65 ± 0.70	**27**	1.15 ± 0.17
**13**	12.41 ± 0.26	**28**	1.37 ± 0.39
**14**	1.80 ± 0.09	**TAM**	1.16 ± 0.13
**15**	7.77 ± 1.9	**4-OH-TAM**	1.46 ± 0.21
**16**	2.19 ± 0.71	**E2 (10 nM)**	13 ± 2.90
**17**	7.28 ± 3.10		

* Estrogenic activity is compared to DMSO (set as 1), compounds screened at a dose of 1 µM; compounds were screened in triplicates.

**Table 4:** EC_50_ values (agonistic activity) of selected compounds.

Code	EC_50_ (nM)
**3**	40.1 ± 0.5
**4**	258 ± 80
**15**	440 ± 10
**16**	n.c. *
**17**	252 ± 8
**18**	n.c.
**19**	407 ± 86
**20**	n.c.
**21**	735 ± 13
**E2**	0.528 ± 0.051

* n.c. = not calculable because no upper plateau is detectable; compounds were screened in triplicates.

**Table 5:** Percent mean growth inhibition on 60 NCI tumor cell lines and on MCF-7 cells.

Code	Mean Growth Inhibition (%)	Growth Inhibition on MCF-7 (%) *	Code	Mean Growth Inhibition (%)	Growth Inhibition on MCF-7 (%) *
**3**	2.34	No inhibition	**17**	69.12	85.01
**4**	15.41	No inhibition	**18**	4.23	No inhibition
**5**	15.45	54.99	**19**	60.79	75.88
**6**	37.82	66.24	**20**	46.97	68.25
**7**	29	56.39	**21**	31.21	29.69
**8**	9.4	30.94	**22**	18.62	33.18
**9**	25.41	24	**23**	46.89	73.52
**10**	33.44	63.07	**24**	29.19	49.32
**11**	67.76	86.96	**25**	12.77	2.75
**12**	55.21	79.65	**26**	28.33	36.51
**13**	7.86	1.21	**27**	47.17	68.34
**14**	47.33	63.49	**28**	92.33	>100
**15**	47.91	86.98	**TAM**	>100	>100
**16**	77.24	90.04			

* Data obtained from NCI in vitro disease-oriented human tumor cell screen (for details, see the work of [39]), compounds tested at a concentration of 10 µM in triplicates.

**Table 6:** Relative alkaline phosphatase activity after an incubation of 72 h in Ishikawa cells.

Code	1 nM	10 nM	100 nM	1 µM	10 µM
**E2**	n.d. **	6.86 ± 1.60 *	n.d.	n.d.	n.d.
**Tam**	n.d.	n.d.	n.d.	1.40 ± 0.45	n.d.
**OH-Tam**	n.d.	n.d.	n.d.	1.47 ± 0.22	n.d.
**5**	0.93 ± 0.64	0.95 ± 0.37	1.05 ± 0.30	1.08 ± 0.33	0.21 ± 0.16 *
**11**	1.25 ± 0.61	1.13 ± 0.31	1.75 ± 0.50	2.56 ± 0.83 *	1.36 ± 0.38
**12**	0.95 ± 0.02	0.96 ± 0.01	1.14 ± 0.01	1.43 ± 0.07 *	0.42 ± 0.13 *
**19**	1.02 ± 0.13	1.06 ± 0.11	1.75 ± 0.08 *	1.71 ± 0.08 *	0.04 ± 0.04 *

Solvent control (DMSO) was set to 1 * *p* < 0.05 (Tukey test) ** n.d. = not determined.

**Table 7:** Relative uterus wet weight of ovariectomized rats**.**

Code	Mean ± SD g/kg BW
**Vehicle**	0.61 ± 0.07
**E2**	3.85 ± 0.71
**TAM**	1.42 ± 0.30
**12**	1.23 ± 0.18
**19**	1.15 ± 0.18

## Data Availability

Not applicable.

## Supplementary Materials

The following are available online at https://www.mdpi.com/article/10.3390/ijms222212575/s1.
