# Supplementary material for: Manipulating Estrogenic/Anti-Estrogenic Activity of Triphenylethylenes towards Development of Novel Anti-Neoplastic SERMs
Source: Int J Mol Sci. 2021 Nov 22;22(22):12575. doi: 10.3390/ijms222212575 (PMC8621172; doi:10.3390/ijms222212575)

Manipulating Estrogenic/Anti-Estrogenic Activity Of Triphenylethylenes Towards Development Of Novel Anti-Neoplastic SERMs

Heba E. Elnakib[1], Marian M. Ramsis[1], Nouran O. Albably[1], Merna A. Vector[1], Jan J. Weigand[2], Kai Schwedtmann[2], Jannette Wober[3], Oliver Zierau[3], Günter Vollmer[3], Ashraf H. Abadi[1], Nermin S. Ahmed[1]

The first three authors contributed equally to this work.

[1]Faculty of Pharmacy and Biotechnology, Department of Pharmaceutical Chemistry, German University in Cairo, Cairo, Egypt.

[2]Faculty of Chemistry and Food Chemistry, Institute of Inorganic Molecular Chemistry, Technische Universität Dresden, 01062 Dresden, Germany.

[3]Faculty of Biology, Institute of Zoology, Technische Universität Dresden, 01062 Dresden, Germany.

*Correspondence: Assoc. Prof. Nermin S. Ahmed, Faculty of Pharmacy and Biotechnology, Department of Pharmaceutical Chemistry, German University in Cairo, 11835 Cairo, Egypt. Email: nermin.salah@guc.edu.eg

Contents

1. Compounds characterization 3

2. Dose response curves for to determine EC_{50} values (agonistic activity) of selected compounds in YES assay, results of AlkP and uterotrophic assays 48

Figure S1: Relative ß-galactosidase activity of selected compounds after two days treatment in YES assay. DMSO was used as solvent control and set to 1, mean ± SD were calculated by three independent experiments. 48

Table S1: Relative Alkaline phosphatase activity (AlkP) after an incubation of 72 h in Ishikawa cells, compounds are tested at a concentration of 1 µM.. 49

Figure S2: Relative Alkaline phosphatase activity (AlkP) for compounds **5, 11, 12, 19** after three days treatment in human endometrial adenocarcinoma Ishikawa cells. DMSO was used as solvent control and set to 1, mean ± SD were calculated by three independent experiments. Tam and 4-OH-Tam (1 µM) were tested for comparison. * $p < 0.05$ versus DMSO control (Tukey test) 50

Figure S3: Relative uterus wet weight following a three days treatment of ovariectomized rats (vehicle = control) with E2 (10 µg/kg BW/ day), TAM (10 mg/kg BW/ day), compound **12** (10 mg/kg BW/ day), and compound **19** (10 mg/kg BW/ day). * $p < 0.05$ versus control (Tukey test)51

Figure S4: DTP log (GI_{50}, TGI, LC_{50}) µM TAM (NSC 180973) 52

Figure S5: Dose response curves compound 11 on different subpanels (µM)............................... 53

Figure S6: Dose response curves compound 12 on different subpanels (µM)............................... 54

Figure S7: Dose response curves compound 16 on different subpanels (µM)............................... 55

Figure S8: Dose response curves compound 17 on different subpanels (µM)............................... 56

Figure S9: Dose response curves compound 19 on different subpanels (µM)............................... 57

Figure S10: Dose response curves compound 19 on different subpanels (µM)............................. 58

1. Compounds characterization

LC-MS / ^{1}H-NMR and ^{13}C-NMR

***E/Z*-4-[1-(4-Chloro-phenyl)-2-phenylpropenyl]-phenol (1)**

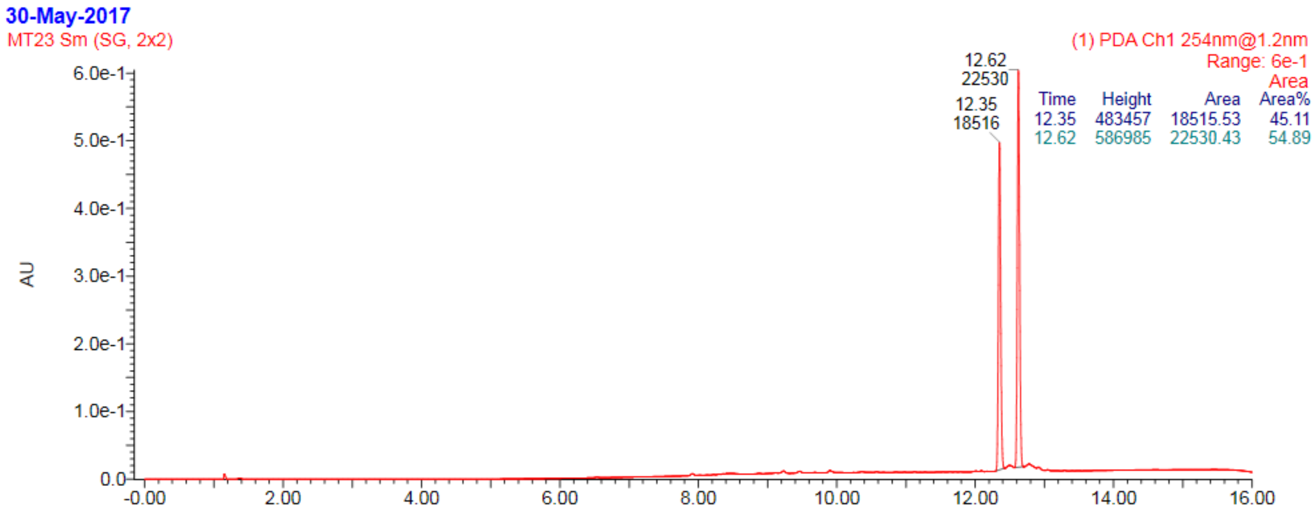

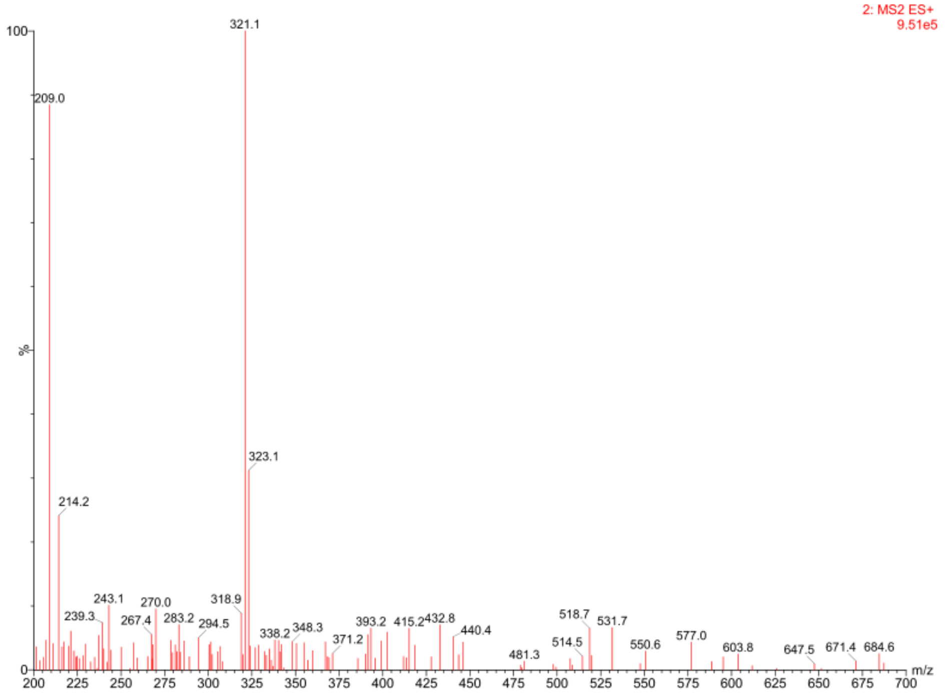

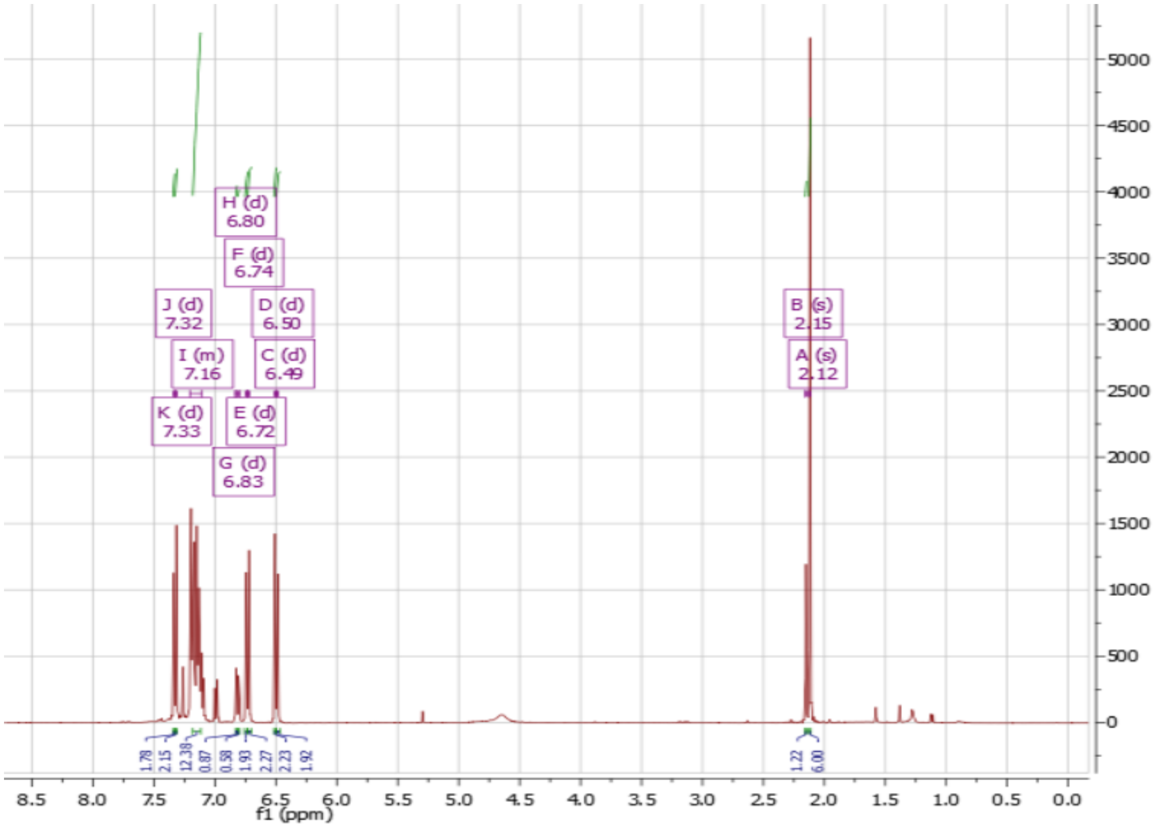
H (d) 6.80
F (d) 6.74
J (d) 7.32
D (d) 6.50
I (m) 7.16
C (d) 6.49
K (d) 7.33
E (d) 6.72
G (d) 6.83
B (s) 2.15
A (s) 2.12
1.78
2.15
12.38
0.87
0.58
1.93
2.27
2.23
1.92
1.22
6.00
f1 (ppm)

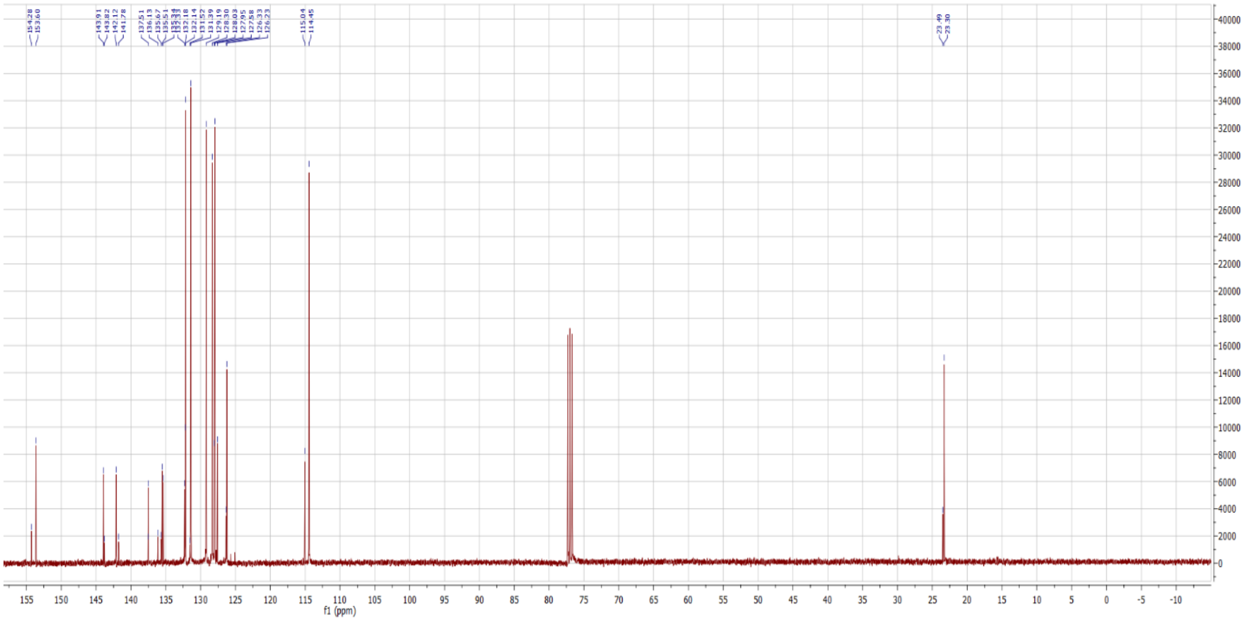
f1 (ppm)

***E*/*Z*-4-[1-(4-Chloro-phenyl)-2-(4-methoxy-phenyl)-propenyl]-phenol (2)**

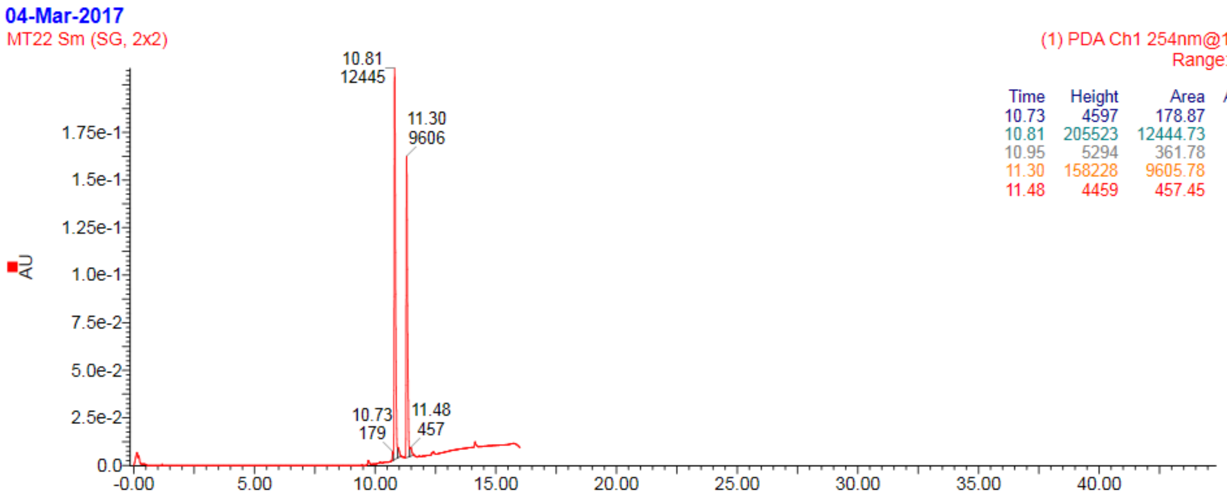

Time	Height	Area	Area%
10.73	4597	178.87	0.78
10.81	205523	12444.73	53.99
10.95	5294	361.78	1.57
11.30	158228	9605.78	41.68
11.48	4459	457.45	1.98

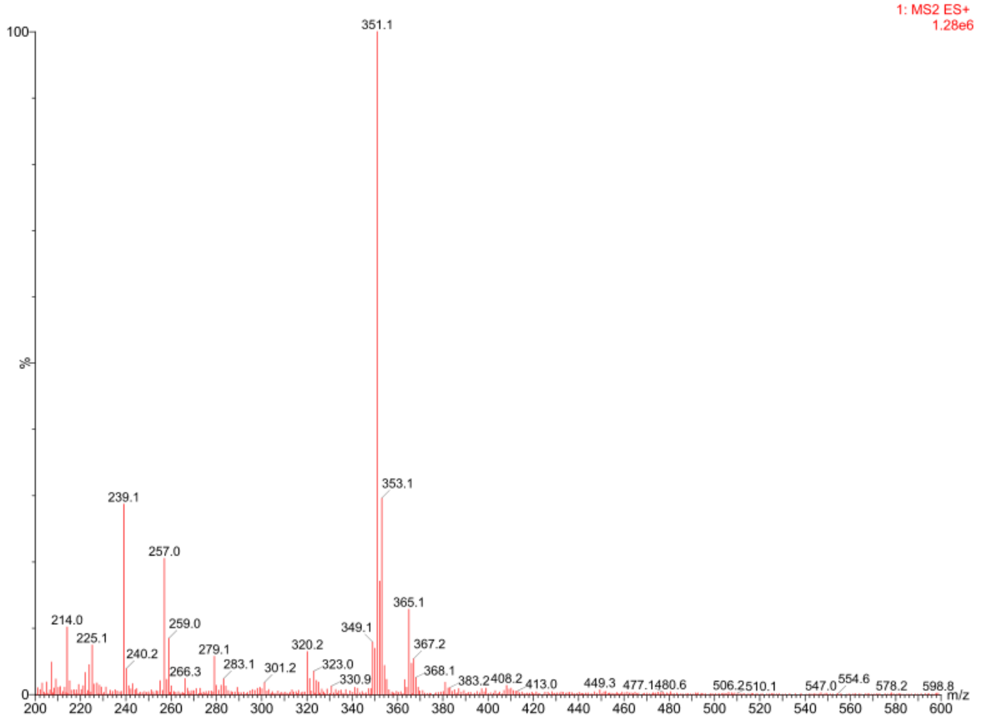

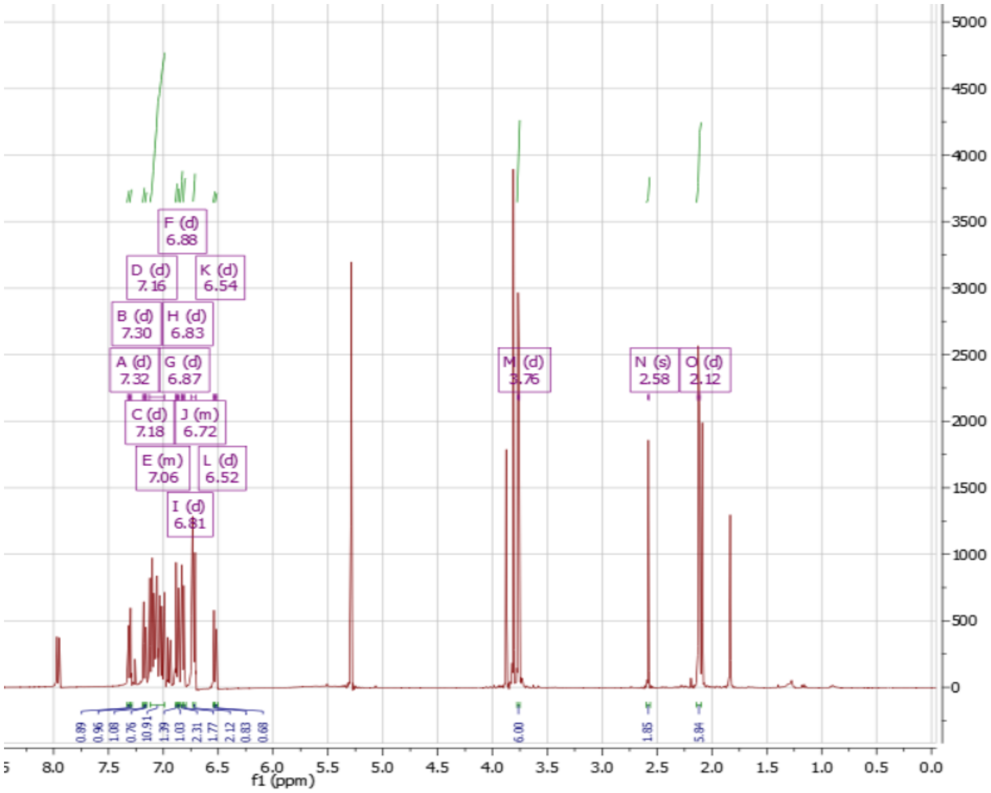

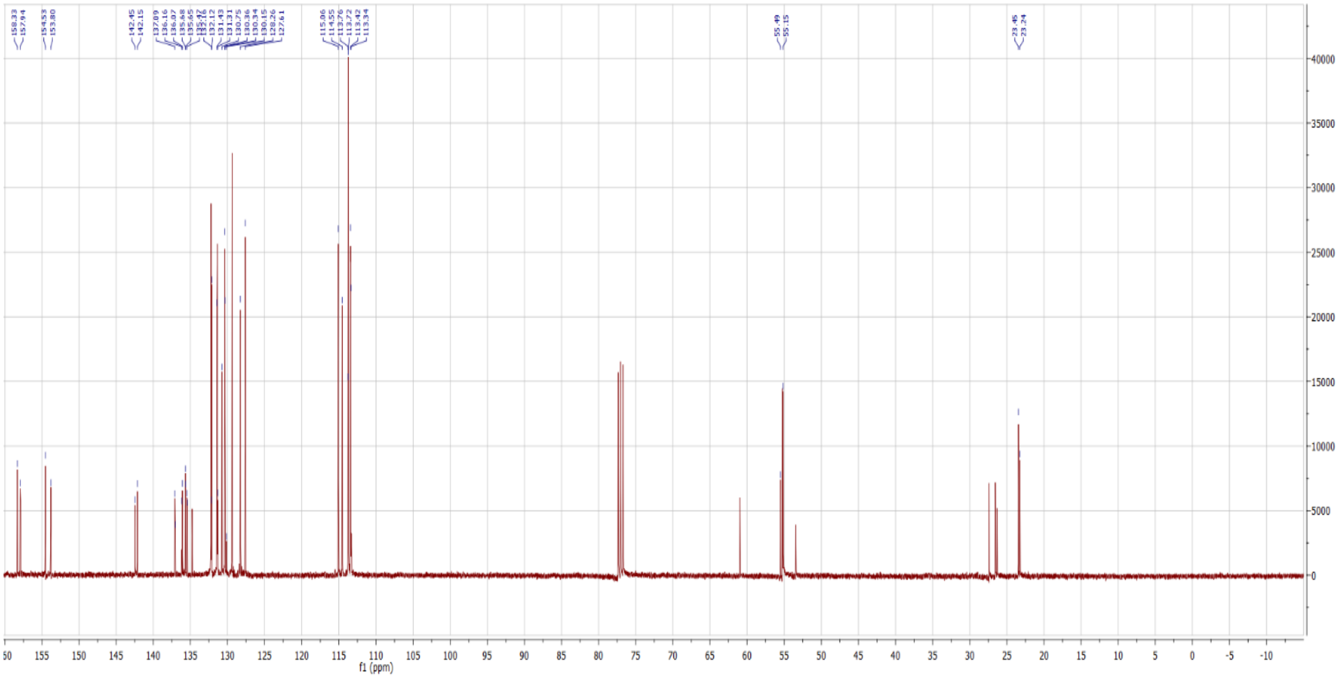

***E/Z*-4-[1-(4-Chlorophenyl)-2-(4-fluoro-3-methoxyphenyl) propenyl]-phenol (4)**

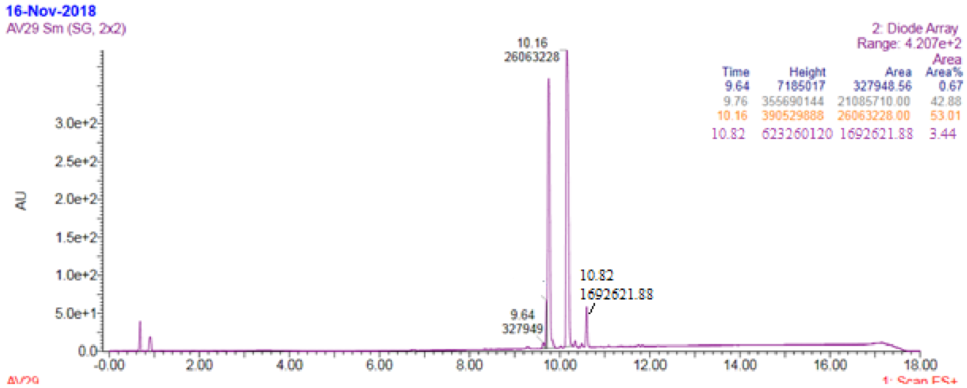

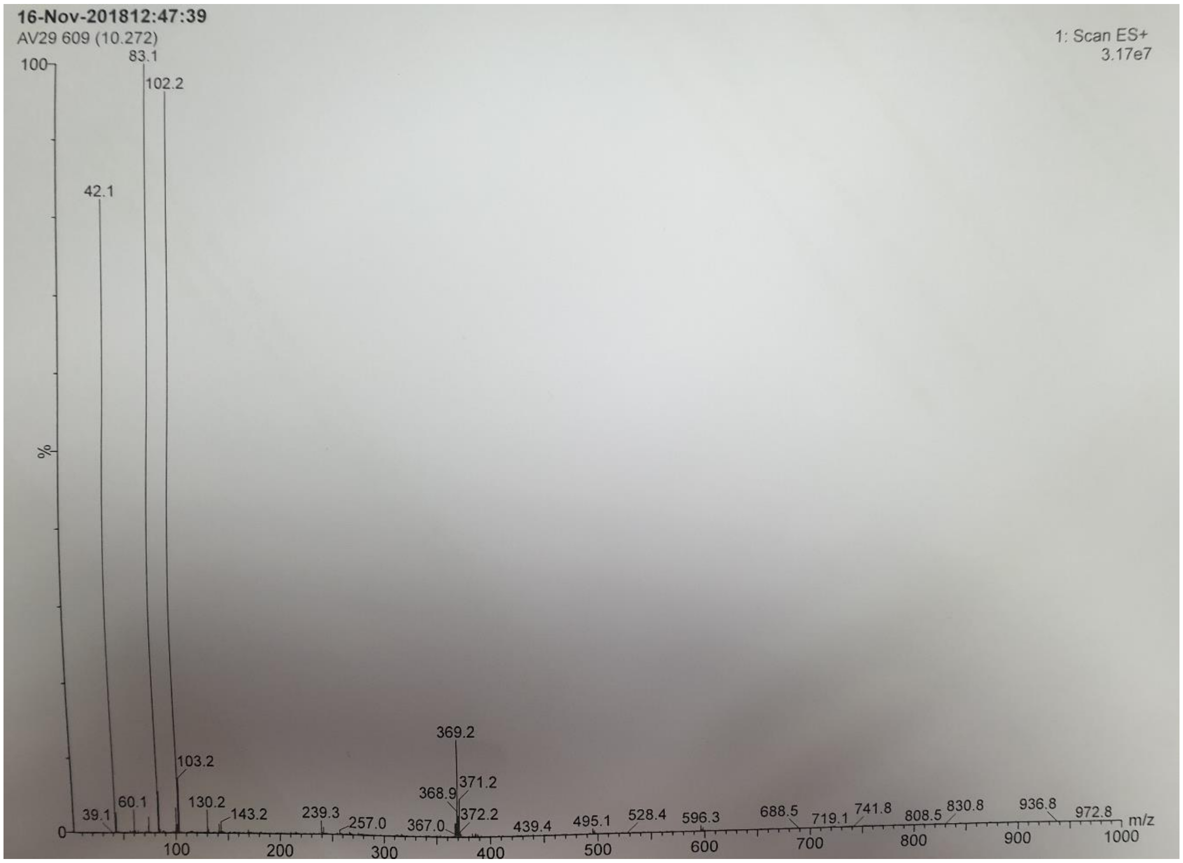

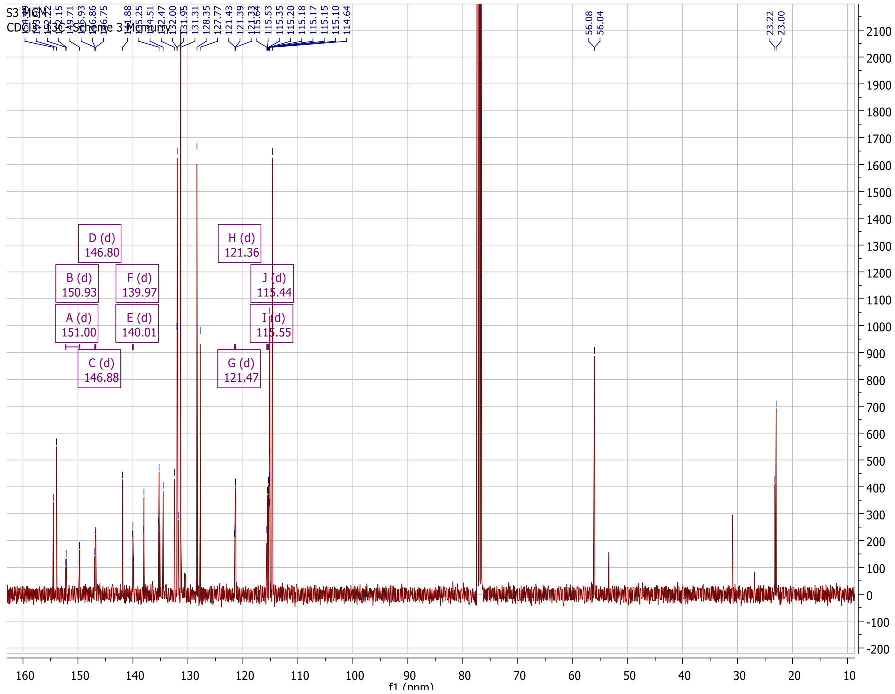

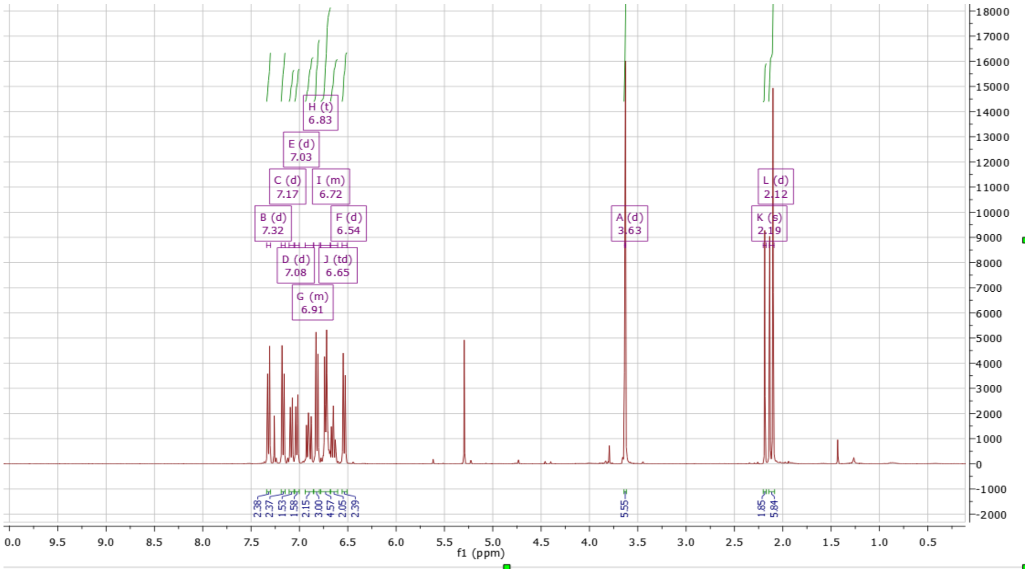

_E_/_Z_-1-(2-{4-[1-(4-Chloro-phenyl)-2-phenyl-propenyl]-phenoxy}-ethyl)-pyrrolidine (6).

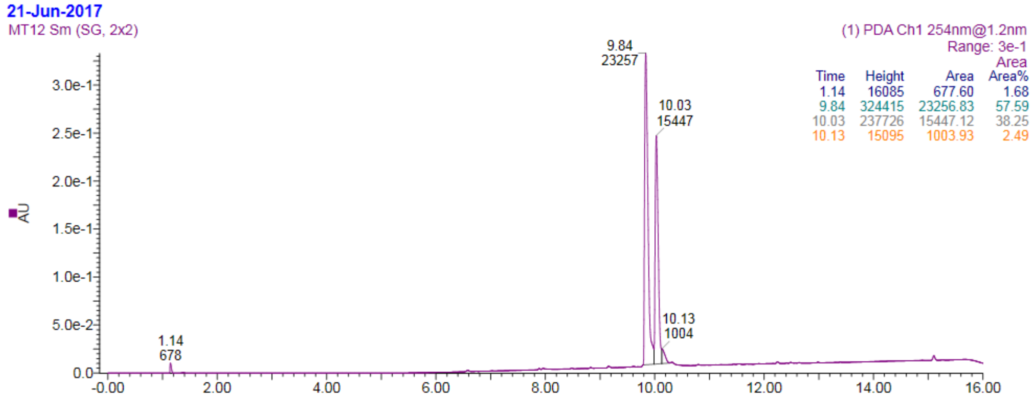
21-Jun-2017
MT12 Sm (SG, 2x2)
(1) PDA Ch1 254nm@1.2nm
Range: 3e-1
Time Height Area Area%
1.14 16085 677.60 1.68
9.84 324415 23256.83 57.59
10.03 237726 15447.12 38.25
10.13 15095 1003.93 2.49
9.84
23257
10.03
15447
10.13
1004
1.14
678
AU

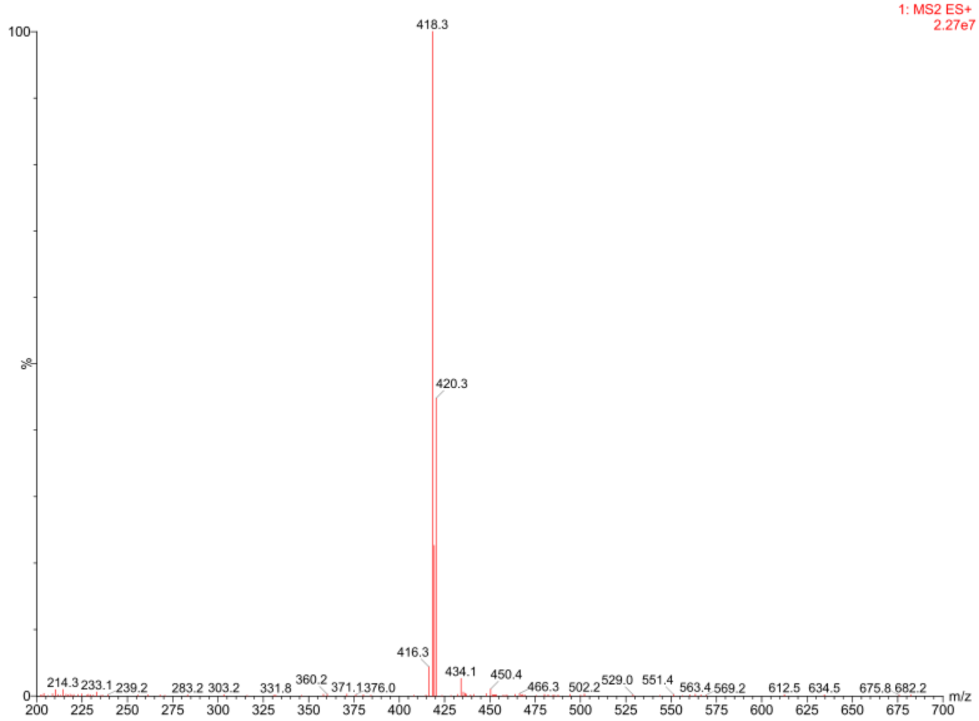
1: MS2 ES+
2.27e7
418.3
420.3
416.3
434.1
450.4
214.3 233.1 239.2 283.2 303.2 331.8 360.2 371.1376.0 466.3 502.2 529.0 551.4 563.4 569.2 612.5 634.5 675.8 682.2
%
m/z

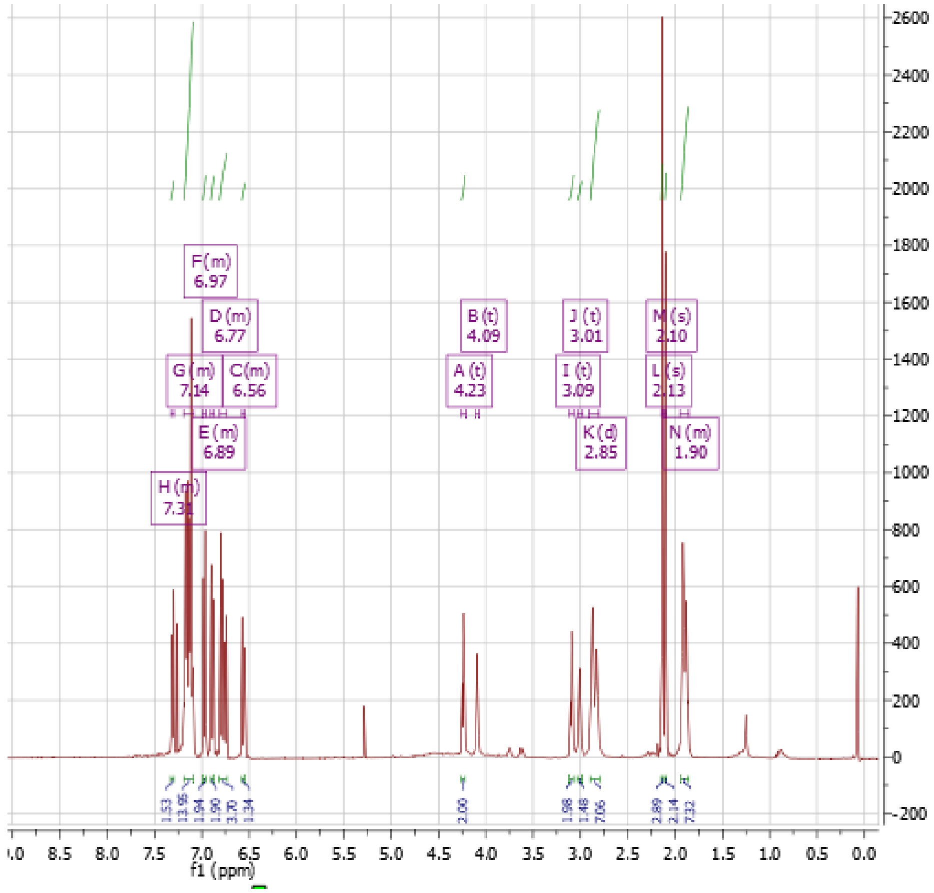

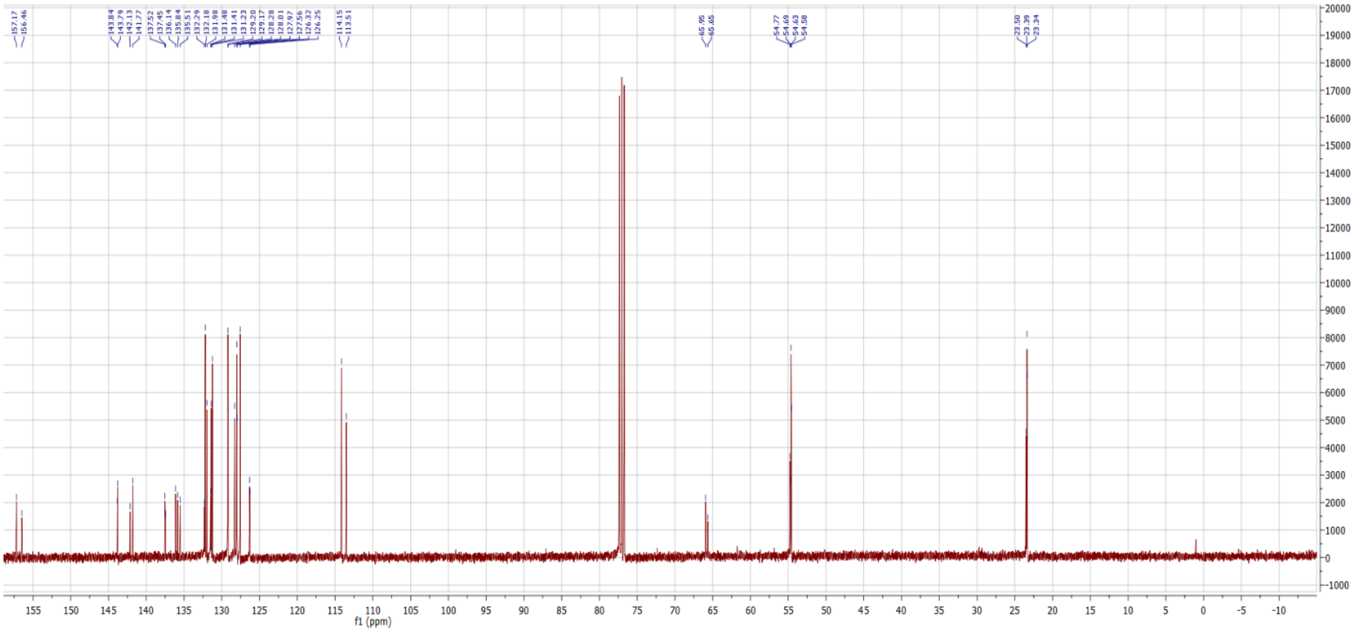

***E/Z*-1-(2-{4-[1-(4-Chloro-phenyl)-2-phenyl-propenyl]-phenoxy}-ethyl)-piperidine (7)**

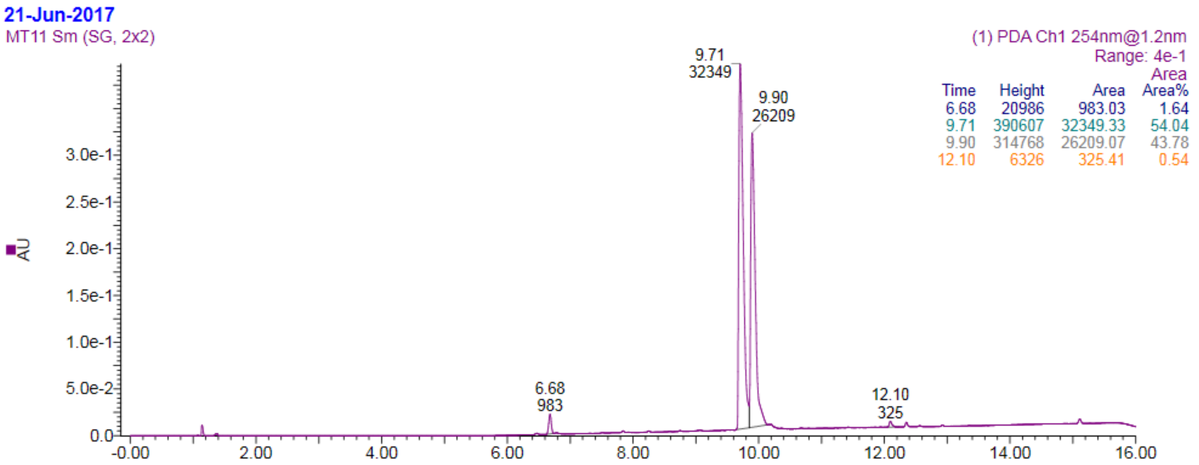

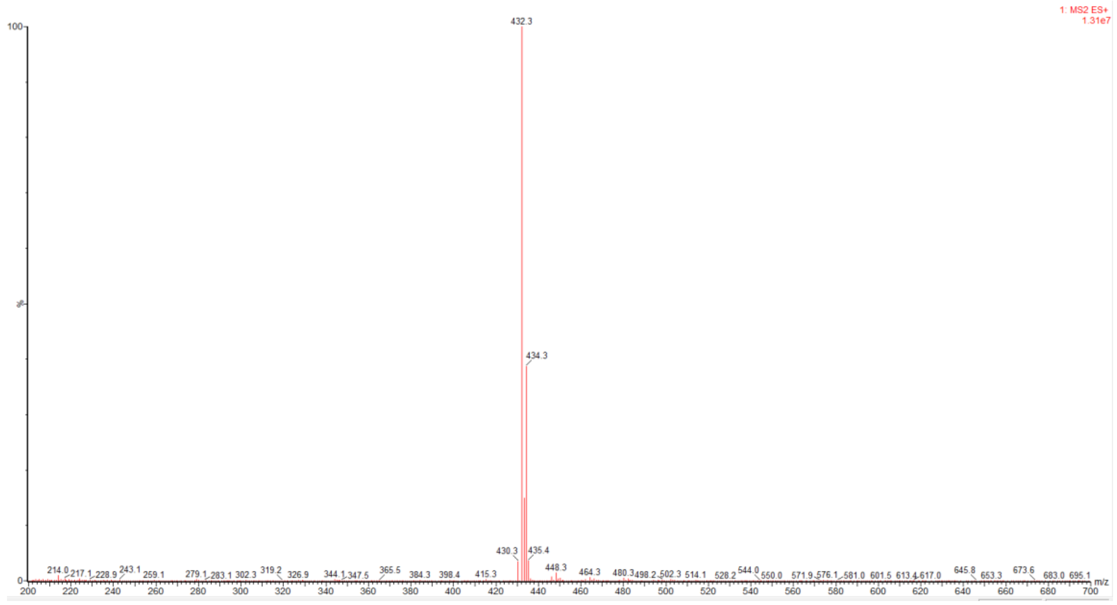

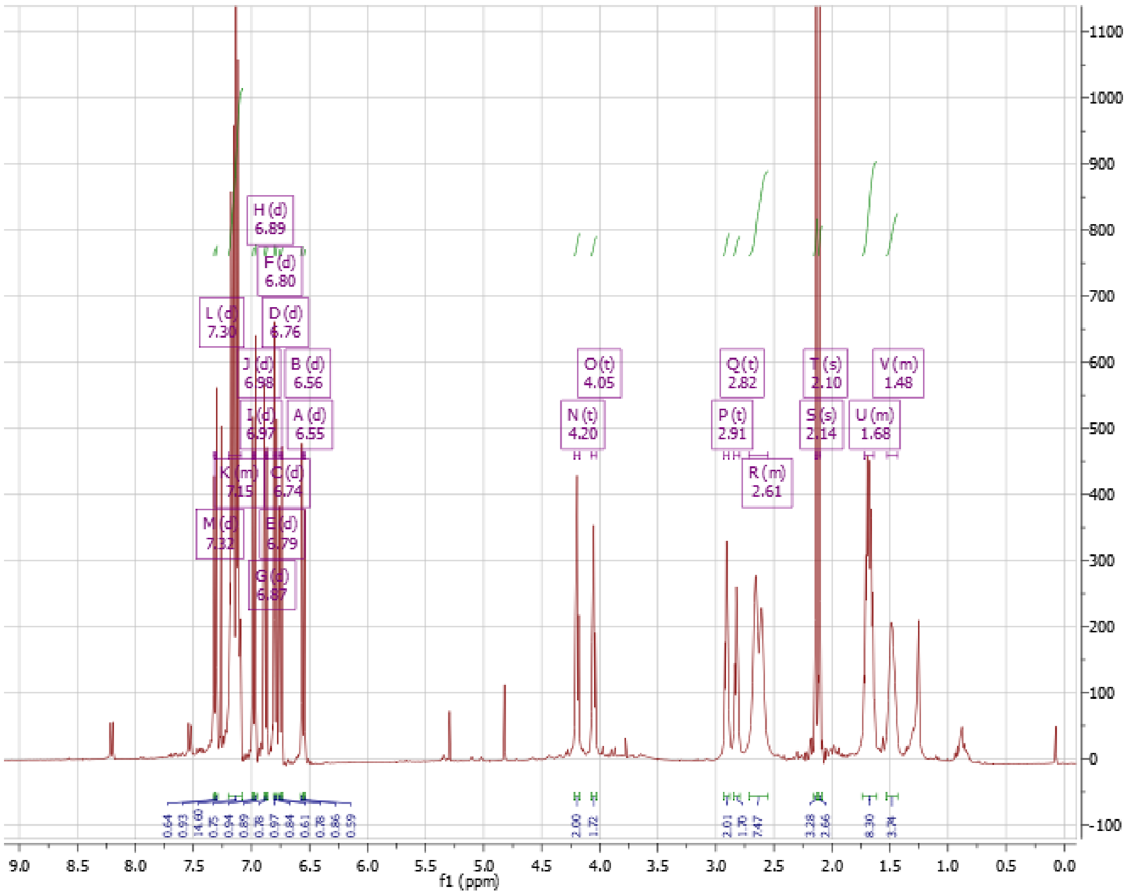
H (d) 6.89
F (d) 6.80
L (d) 7.30
D (d) 6.76
J (d) 6.98
B (d) 6.56
I (d) 6.97
A (d) 6.55
K (m) 7.15
C (d) 6.74
M (d) 7.32
E (d) 6.79
G (d) 6.87
O (t) 4.05
N (t) 4.20
Q (t) 2.82
P (t) 2.91
R (m) 2.61
T (s) 2.10
S (s) 2.14
V (m) 1.48
U (m) 1.68
f1 (ppm)

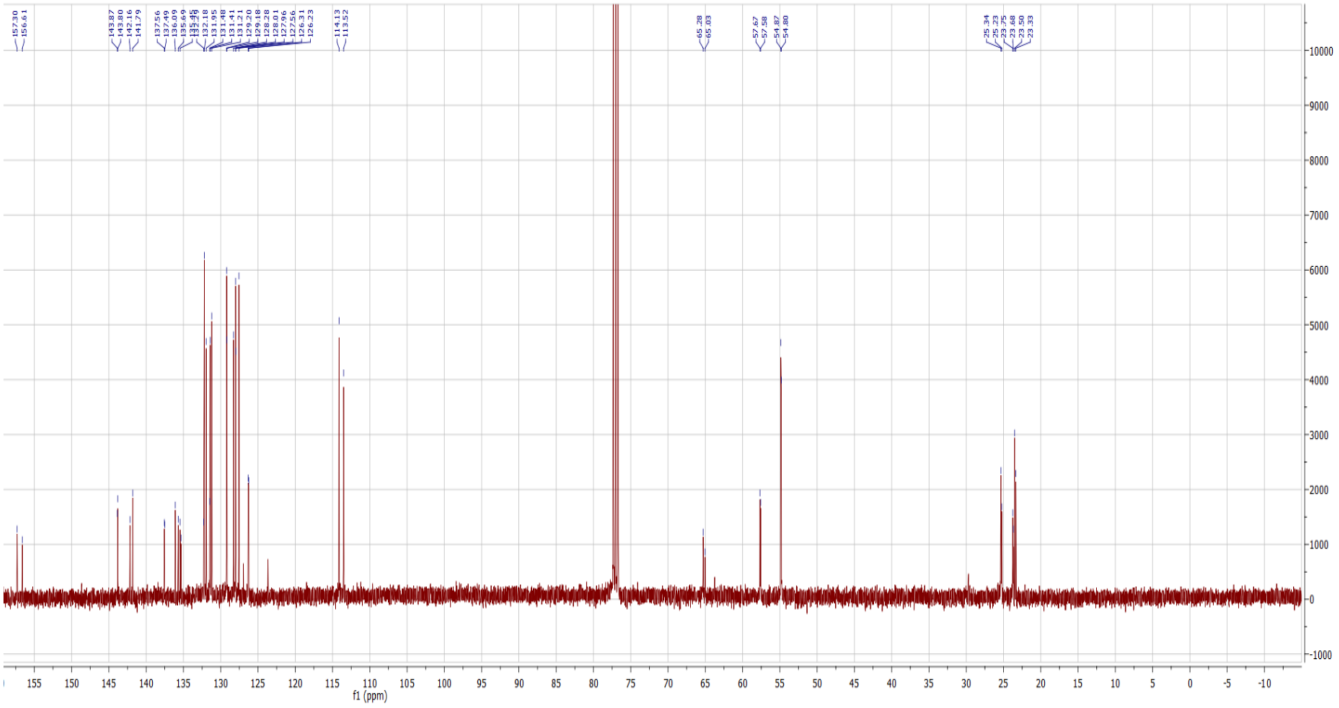
f1 (ppm)

***E/Z*-4-(2-{4-[1-(4-Chloro-phenyl)-2-phenyl-propenyl]-phenoxy}-ethyl)-morpholine (8).**

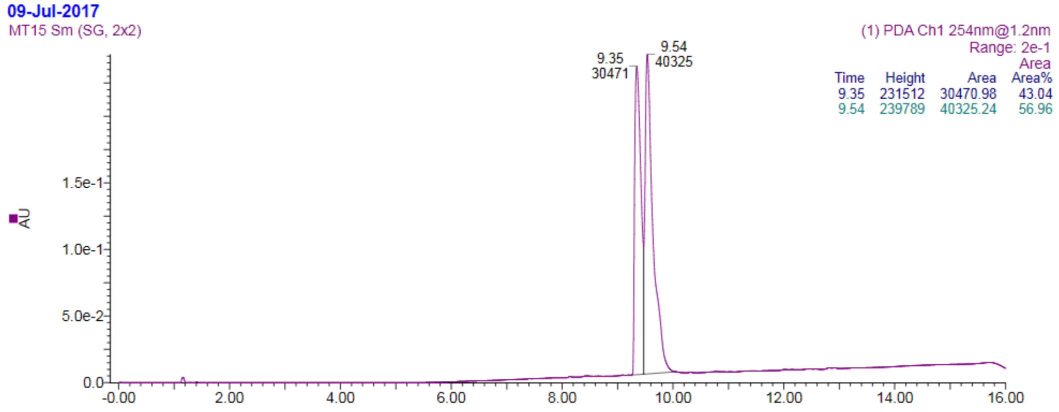

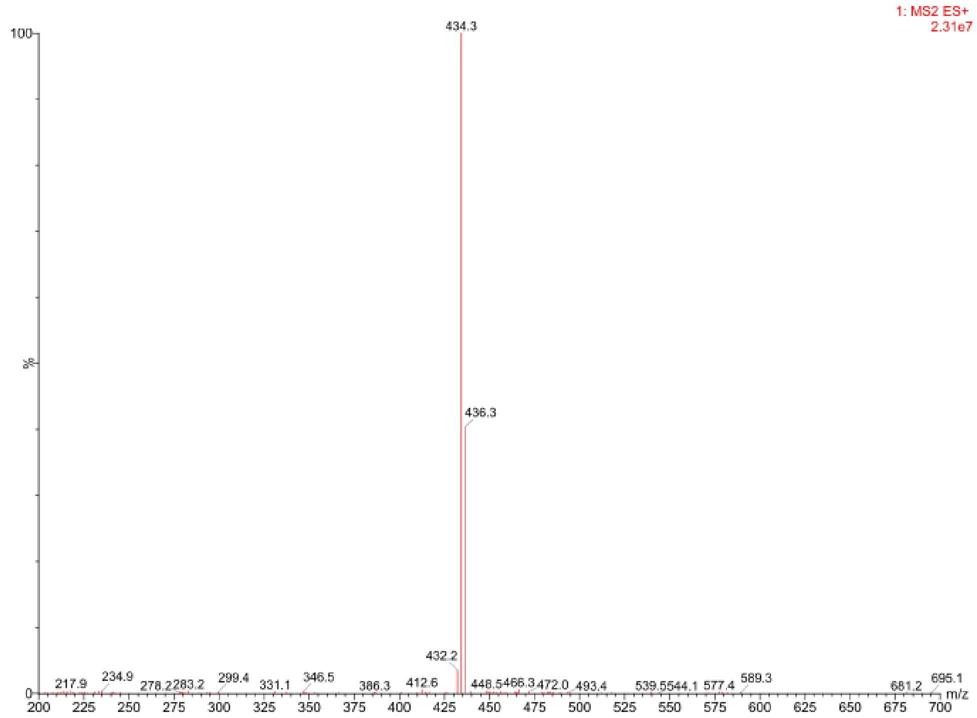

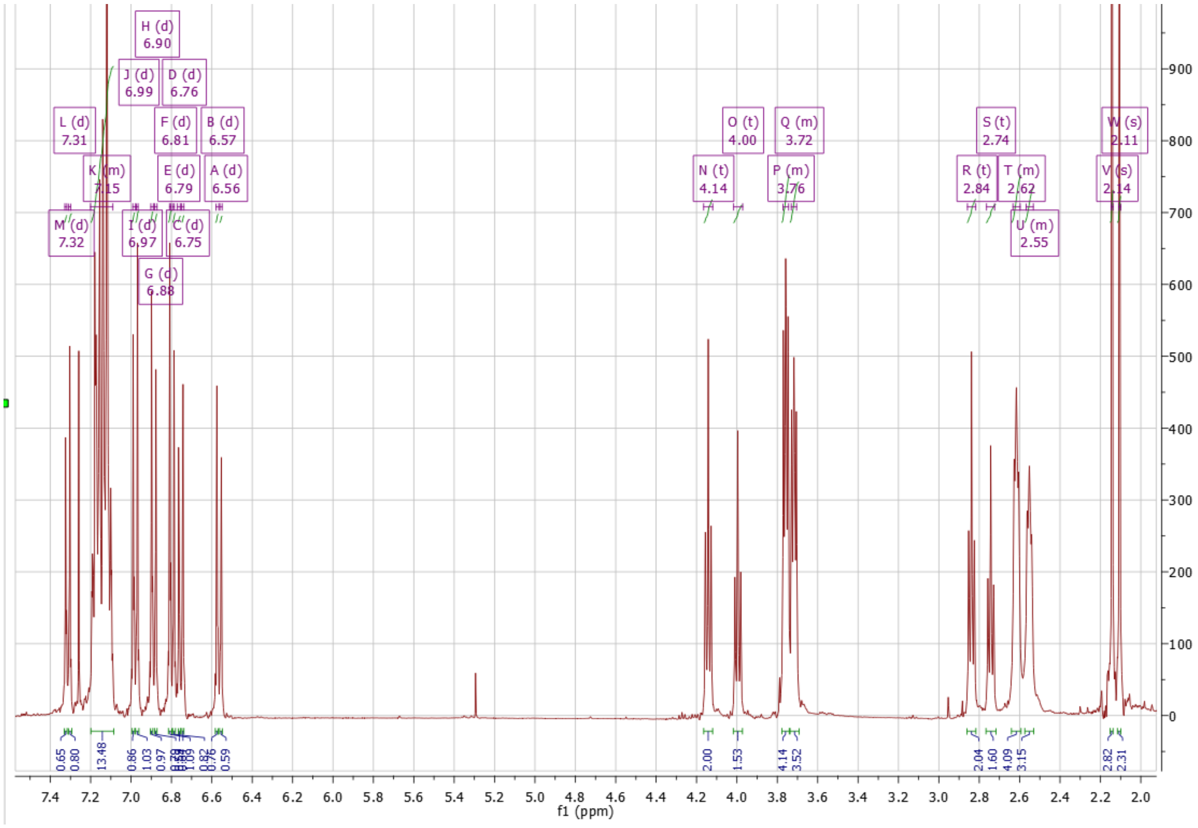
H (d) 6.90
J (d) 6.99
D (d) 6.76
L (d) 7.31
F (d) 6.81
B (d) 6.57
K (m) 7.15
E (d) 6.79
A (d) 6.56
M (d) 7.32
I (d) 6.97
C (d) 6.75
G (d) 6.88
O (t) 4.00
Q (m) 3.72
N (t) 4.14
P (m) 3.76
S (t) 2.74
R (t) 2.84
T (m) 2.62
U (m) 2.55
W (s) 2.11
V (s) 2.14
f1 (ppm)

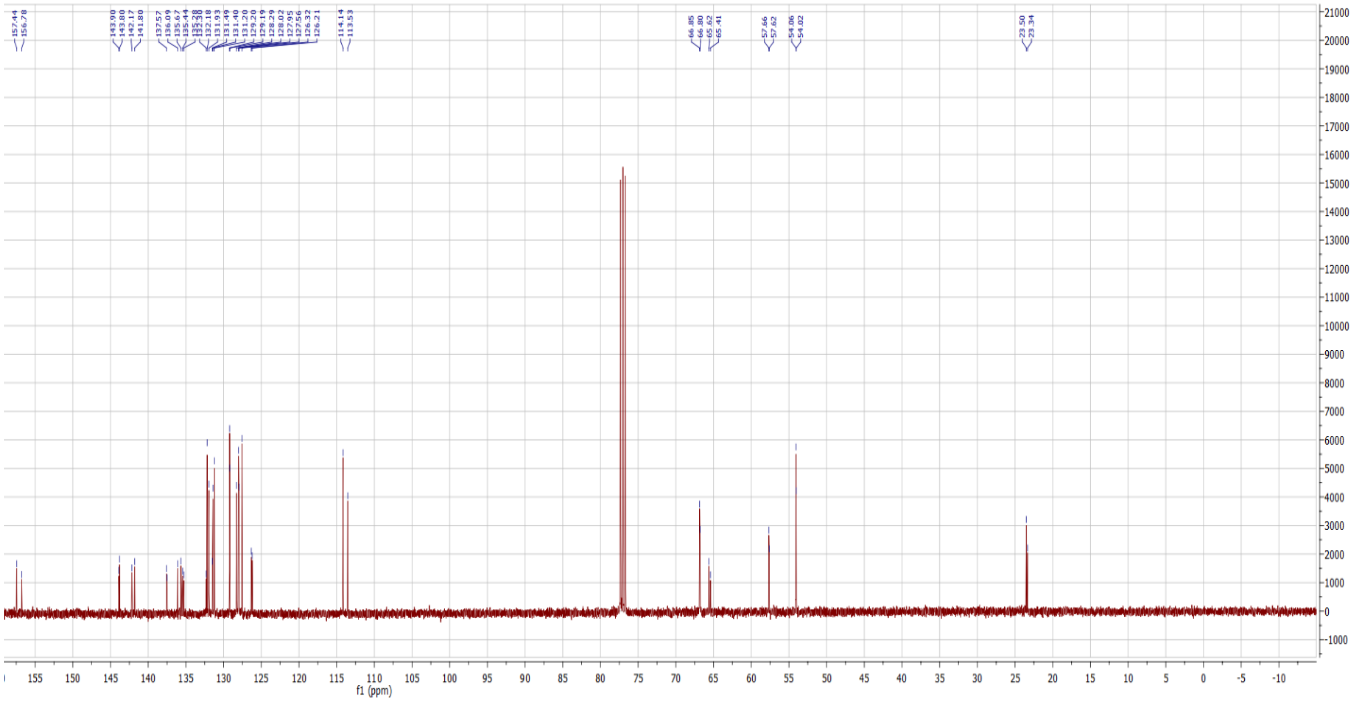
f1 (ppm)

***E*/*Z*-1-(2-{4-[1-(4-Chloro-phenyl)-2-phenyl-propenyl]-phenoxy}-ethyl)-azepane (9).**

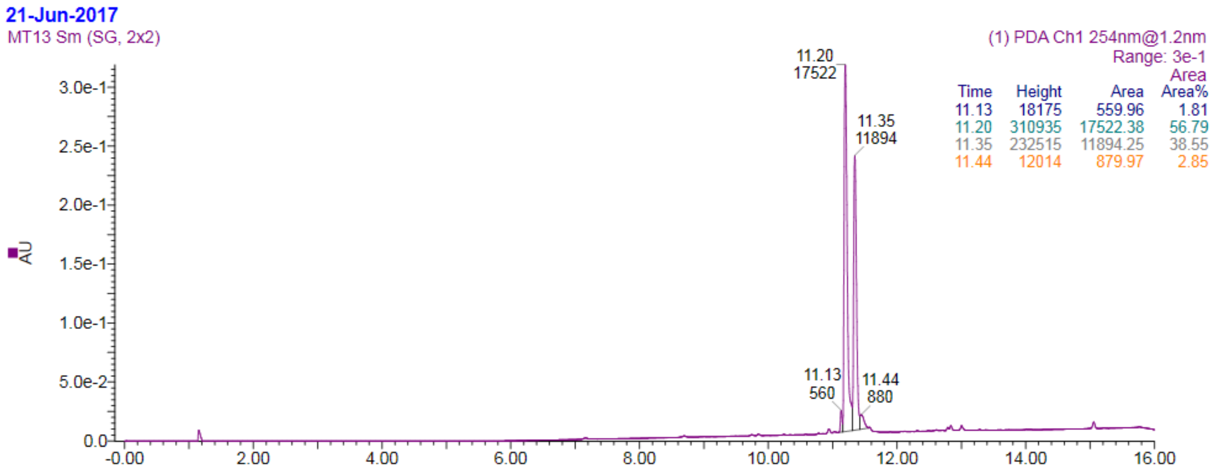

Time	Height	Area	Area%
11.13	18175	559.96	1.81
11.20	310935	17522.38	56.79
11.35	232515	11894.25	38.55
11.44	12014	879.97	2.85

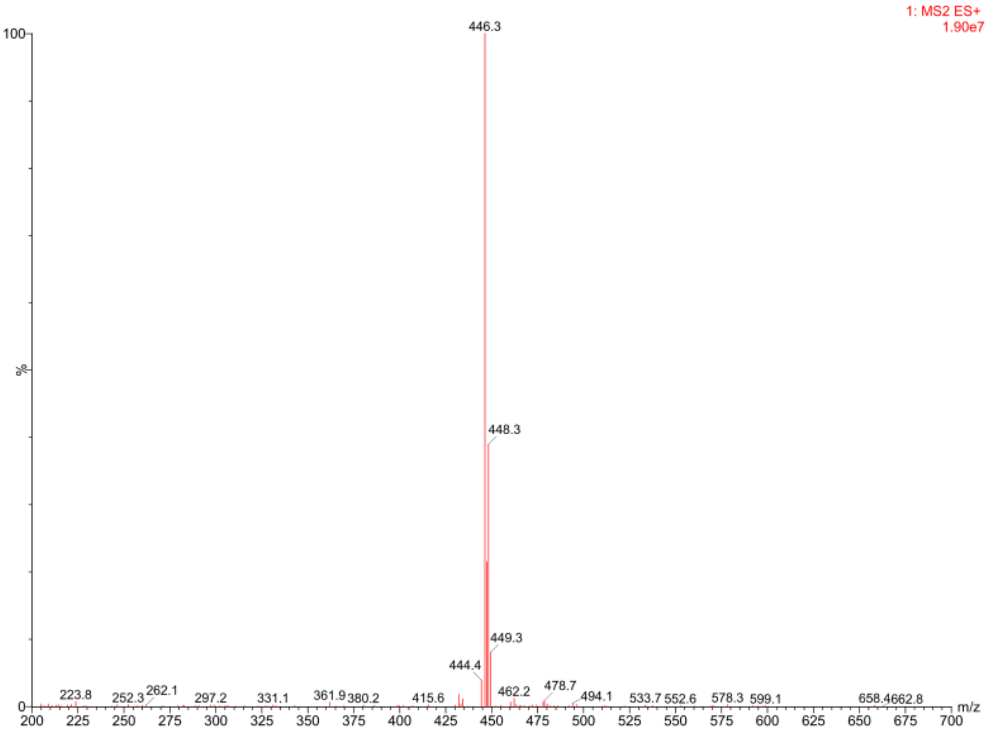

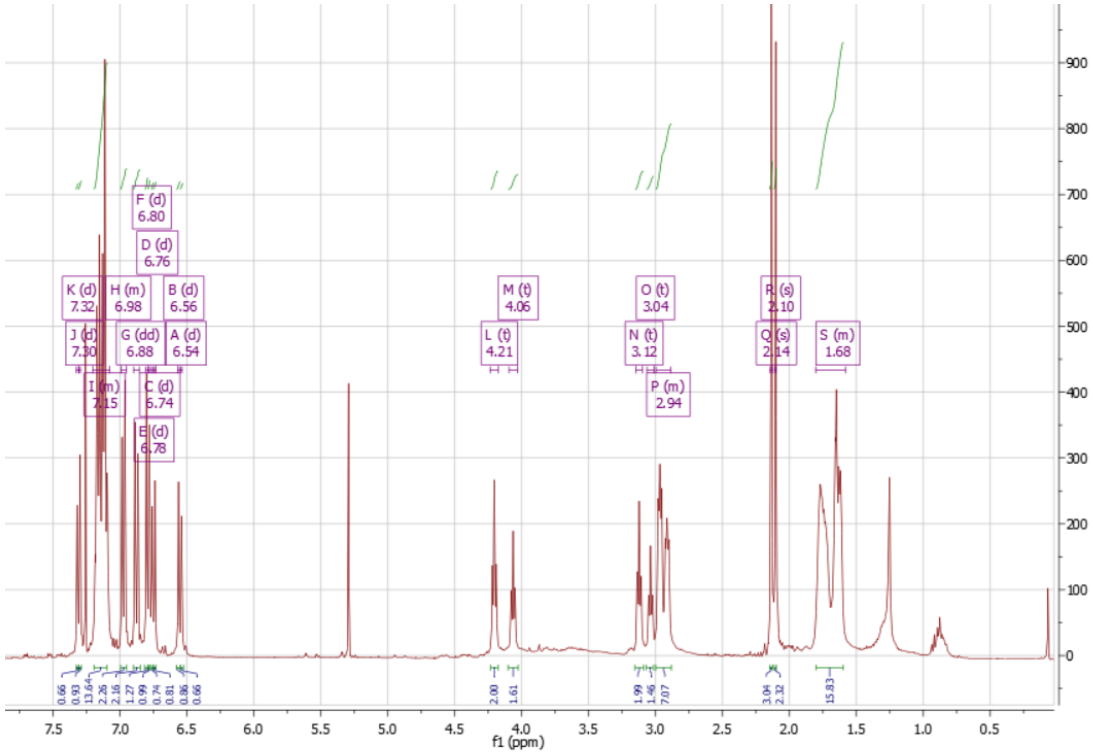

F (d) 6.80
D (d) 6.76
K (d) 7.32
H (m) 6.98
B (d) 6.56
J (d) 7.30
G (dd) 6.88
A (d) 6.54
I (m) 7.15
C (d) 6.74
E (d) 6.78
M (t) 4.06
L (t) 4.21
O (t) 3.04
N (t) 3.12
P (m) 2.94
R (s) 2.10
Q (s) 2.14
S (m) 1.68
f1 (ppm)

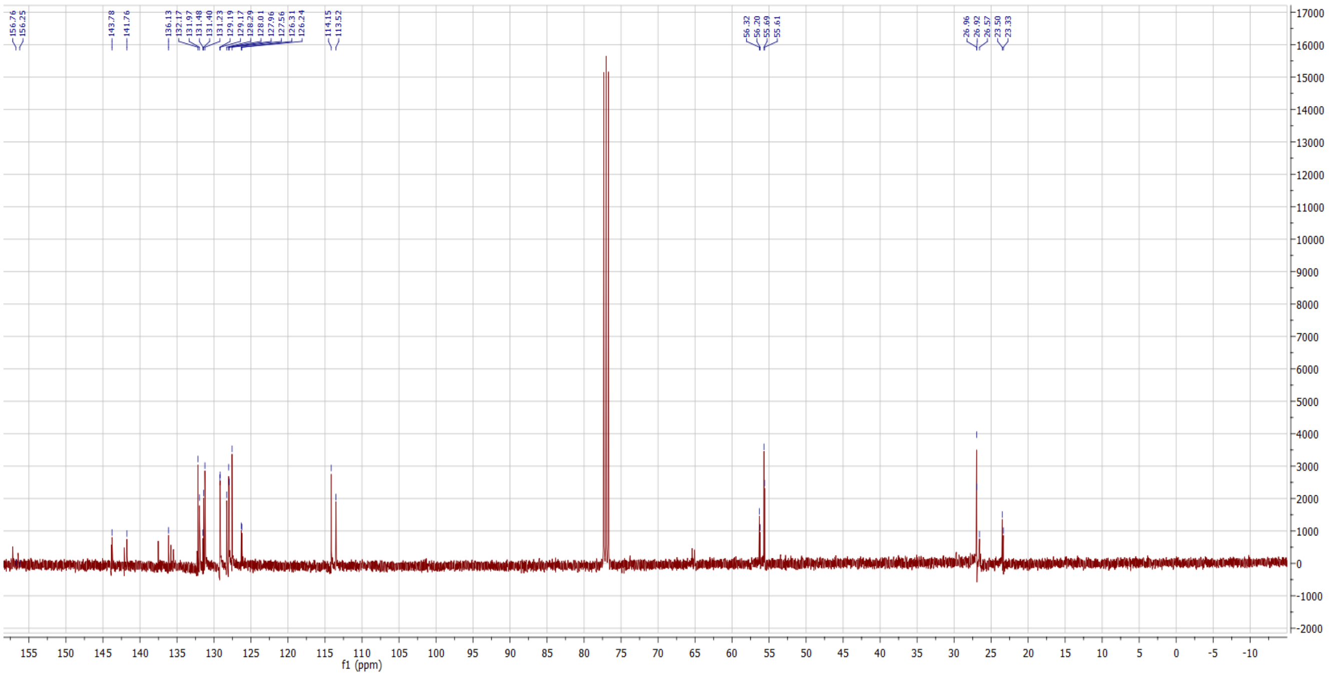

156.76
156.25
143.78
141.76
114.15
113.52
56.32
56.20
55.69
55.61
26.96
26.92
26.57
23.50
23.33
f1 (ppm)

***E/Z*-(3-{4-[1-(4-Chloro-phenyl)-2-(4-methoxy-phenyl)-propenyl]-phenoxy}-propyl)-dimethyl-amine (10).**

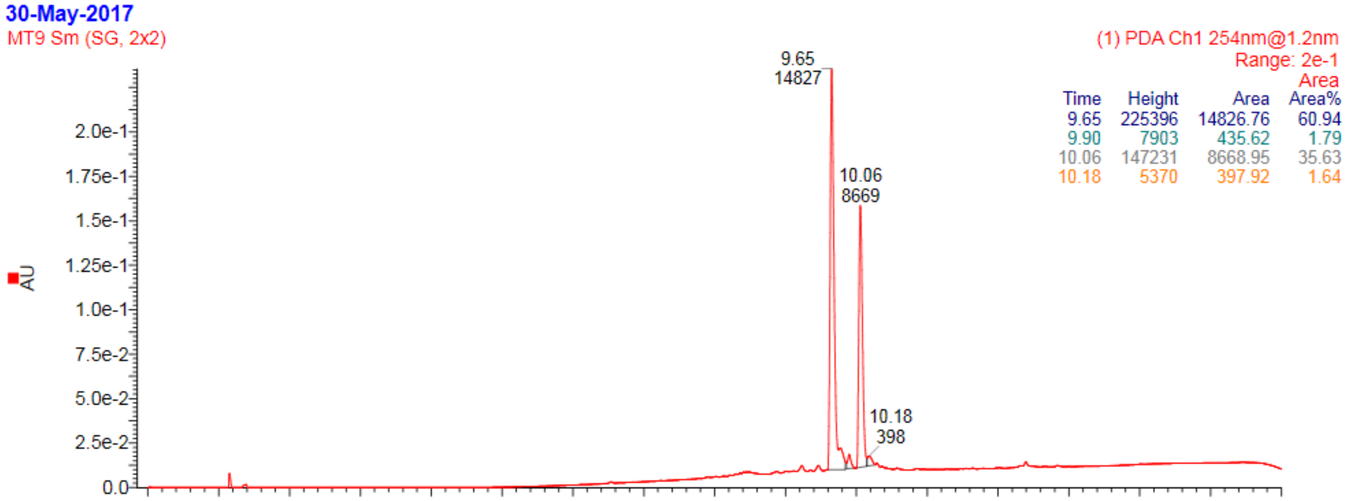

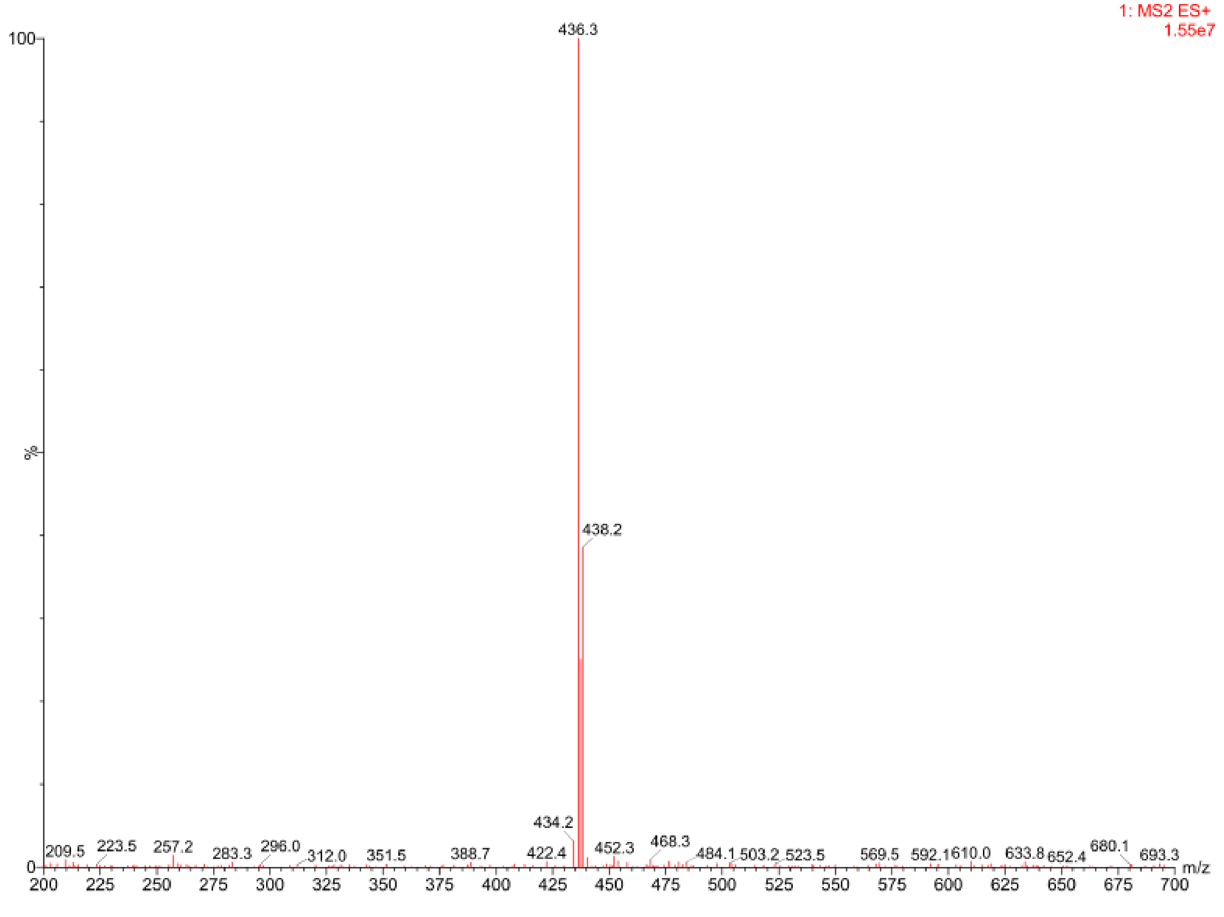

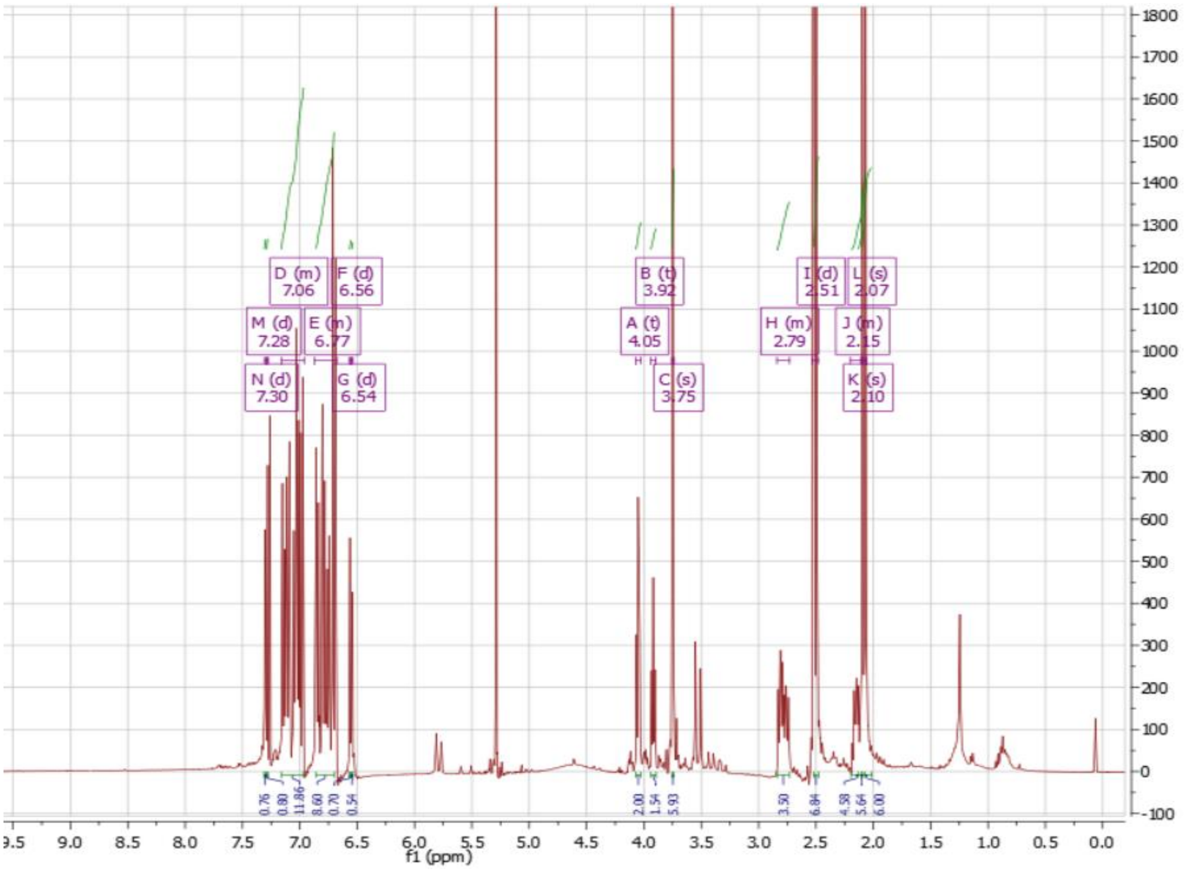

D (m) 7.06
F (d) 6.56
M (d) 7.28
E (m) 6.77
N (d) 7.30
G (d) 6.54
B (t) 3.92
A (t) 4.05
C (s) 3.75
I (d) 2.51
L (s) 2.07
H (m) 2.79
J (m) 2.15
K (s) 2.10
f1 (ppm)

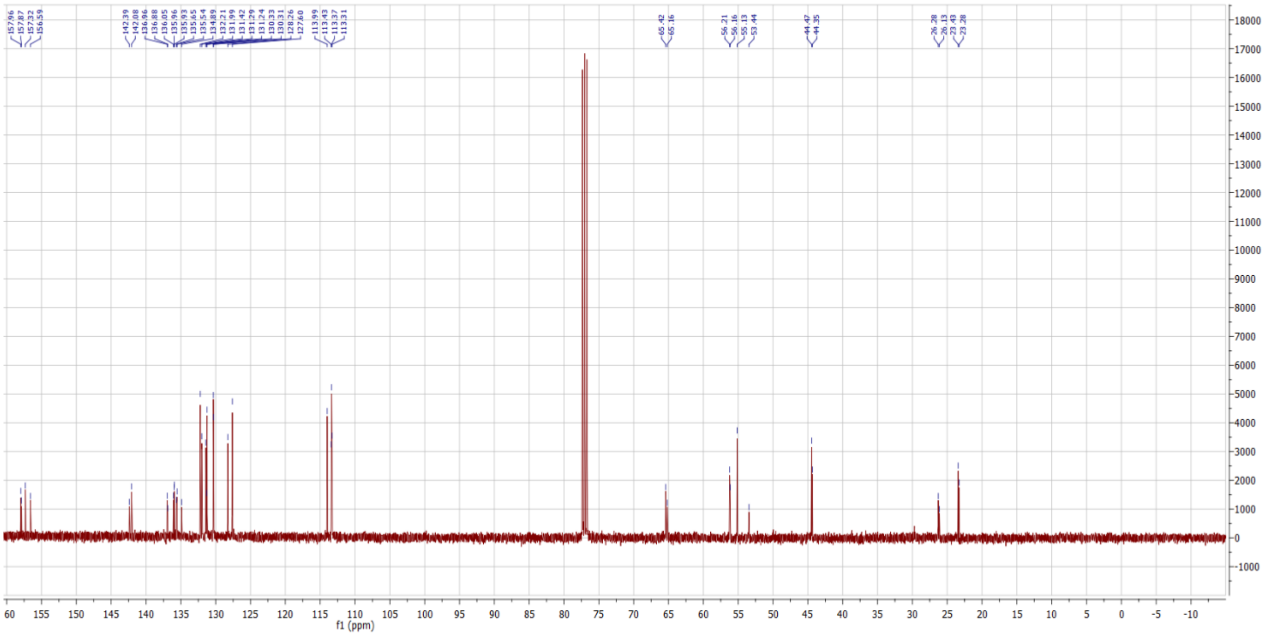

f1 (ppm)

***E/Z*-1-(2-{4-[1-(4-Chloro-phenyl)-2-(4-methoxy-phenyl)-propenyl]-phenoxy}-ethyl)-pyrrolidine (11).**

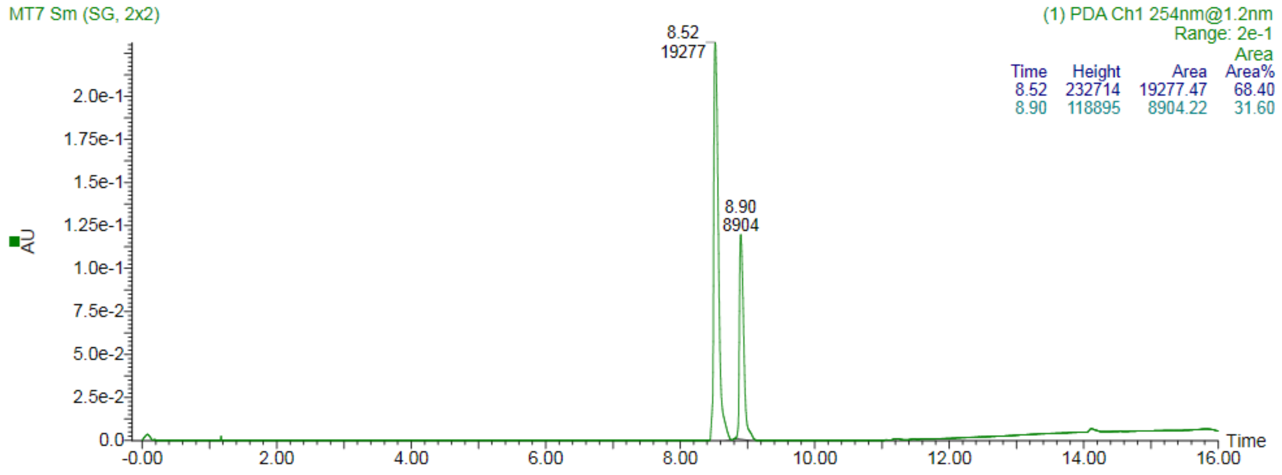

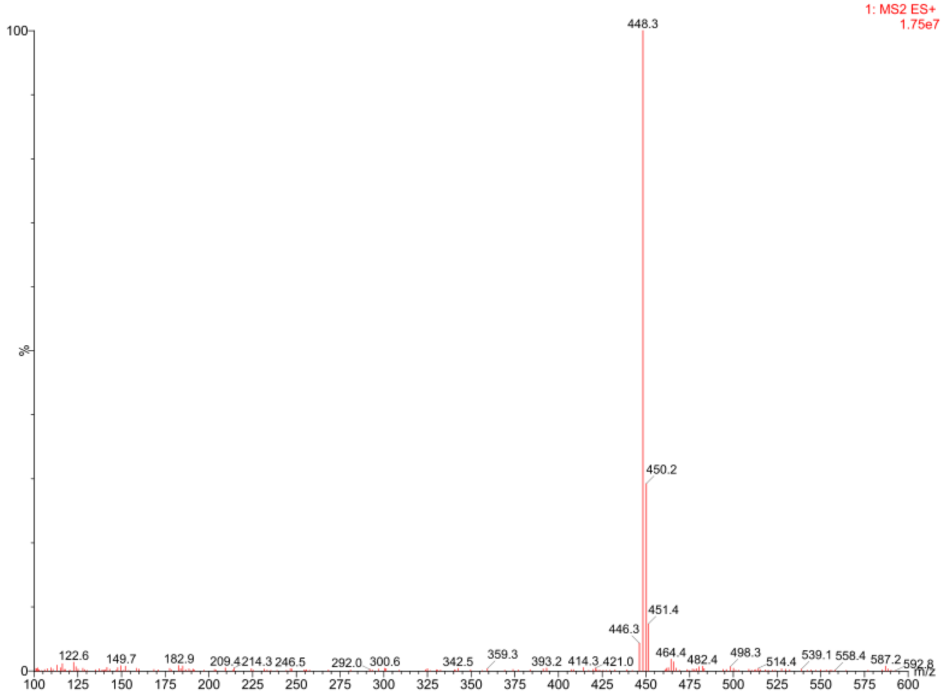

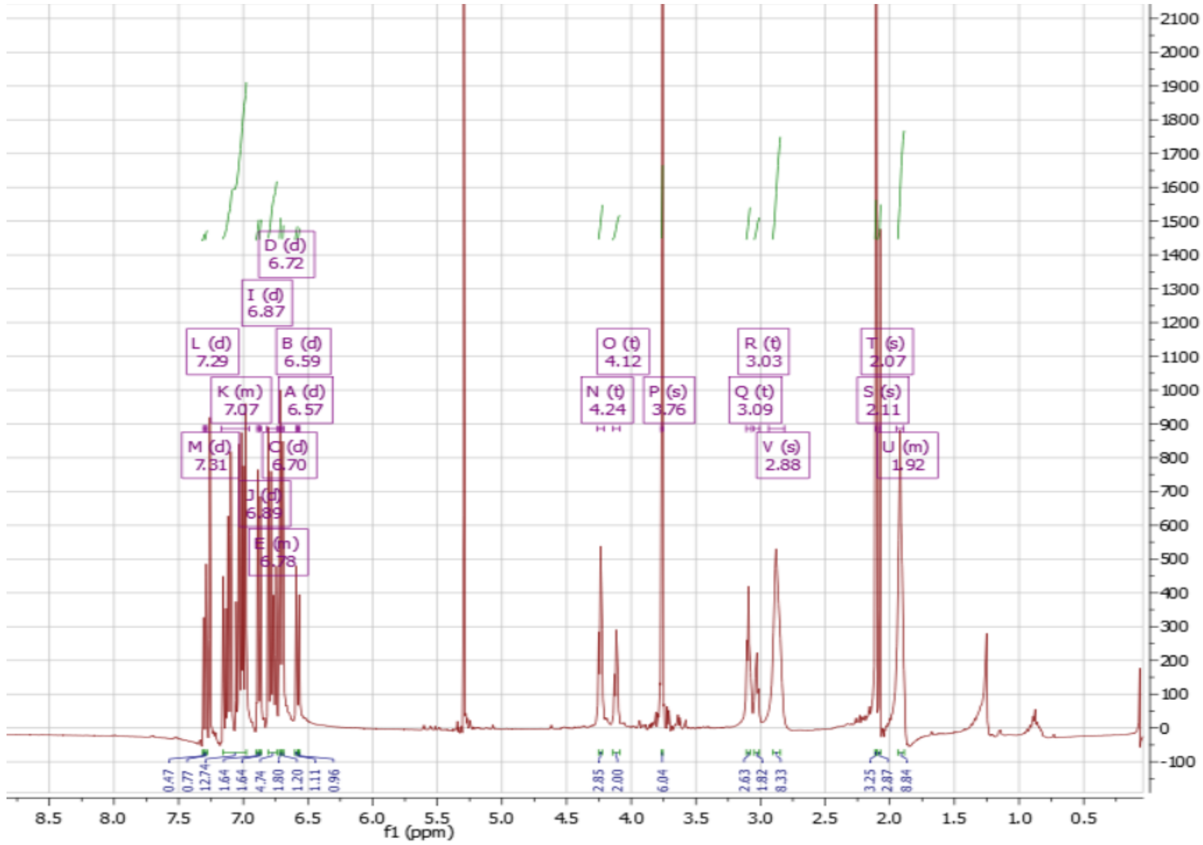

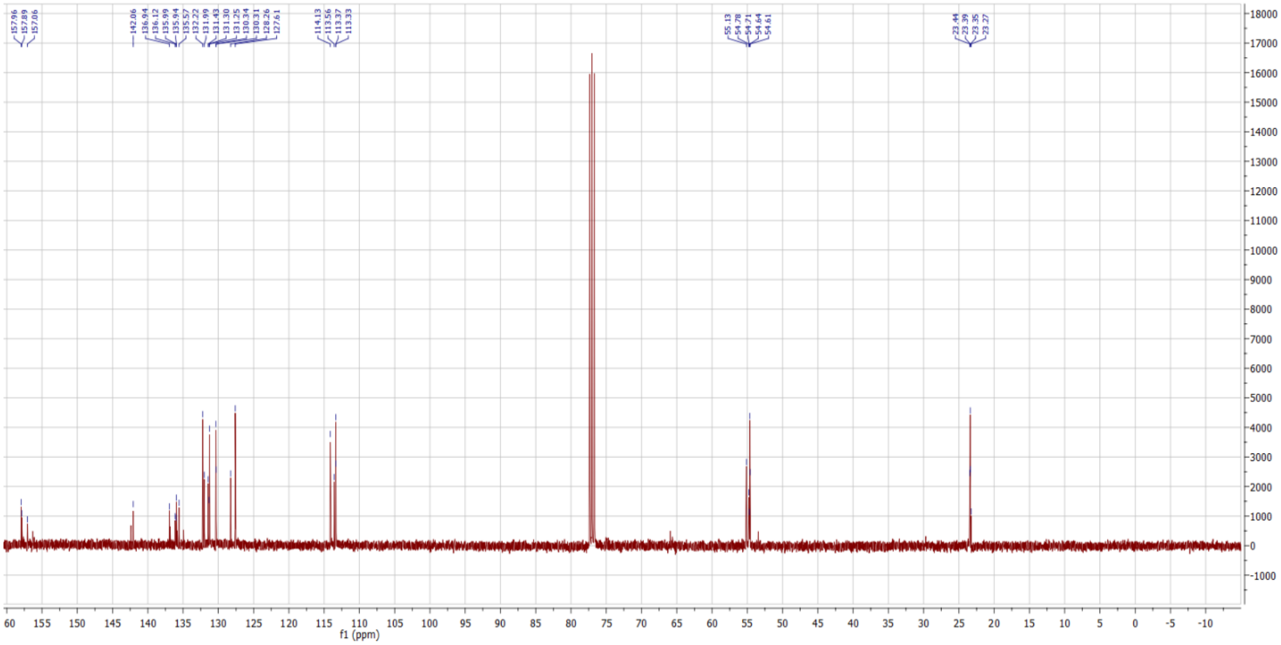

***E*/*Z*-1-(2-{4-[1-(4-Chloro-phenyl)-2-(4-methoxy-phenyl)-propenyl]-phenoxy}-ethyl)-piperidine (12).**

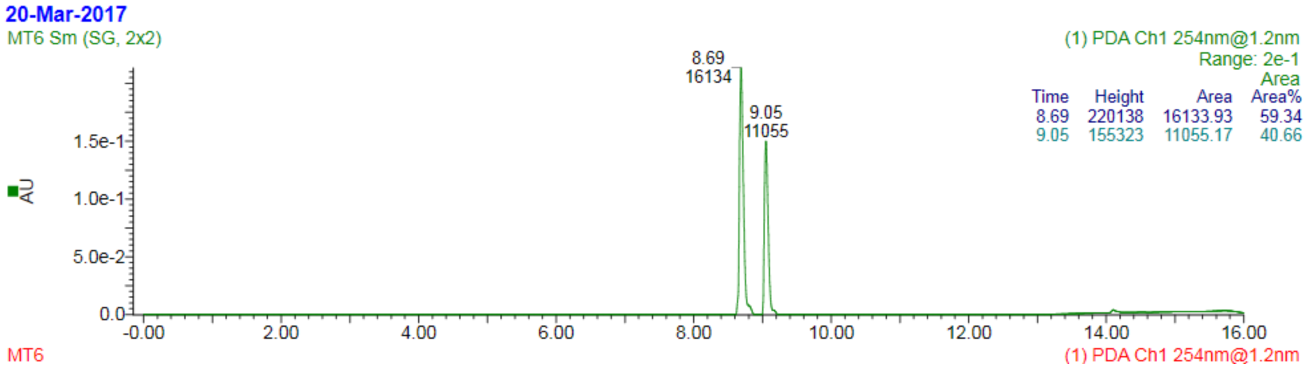
20-Mar-2017
MT6 Sm (SG, 2x2)
(1) PDA Ch1 254nm@1.2nm
Range: 2e-1
8.69
16134
9.05
11055
Time Height Area Area%
8.69 220138 16133.93 59.34
9.05 155323 11055.17 40.66
AU
1.5e-1
1.0e-1
5.0e-2
0.0
-0.00 2.00 4.00 6.00 8.00 10.00 12.00 14.00 16.00
MT6
(1) PDA Ch1 254nm@1.2nm

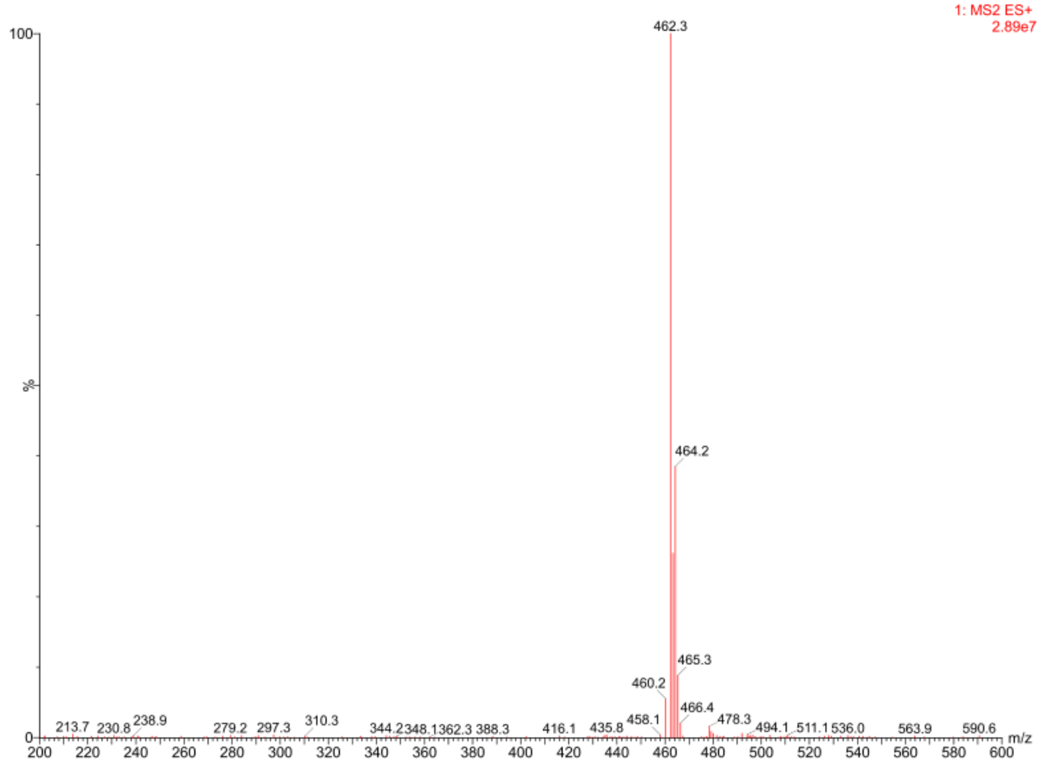
1: MS2 ES+
2.89e7
100
%
0
462.3
464.2
465.3
460.2
466.4
458.1
478.3
213.7 230.8 238.9 279.2 297.3 310.3 344.2 348.1 362.3 388.3 416.1 435.8 494.1 511.1 536.0 563.9 590.6
200 220 240 260 280 300 320 340 360 380 400 420 440 460 480 500 520 540 560 580 600
m/z

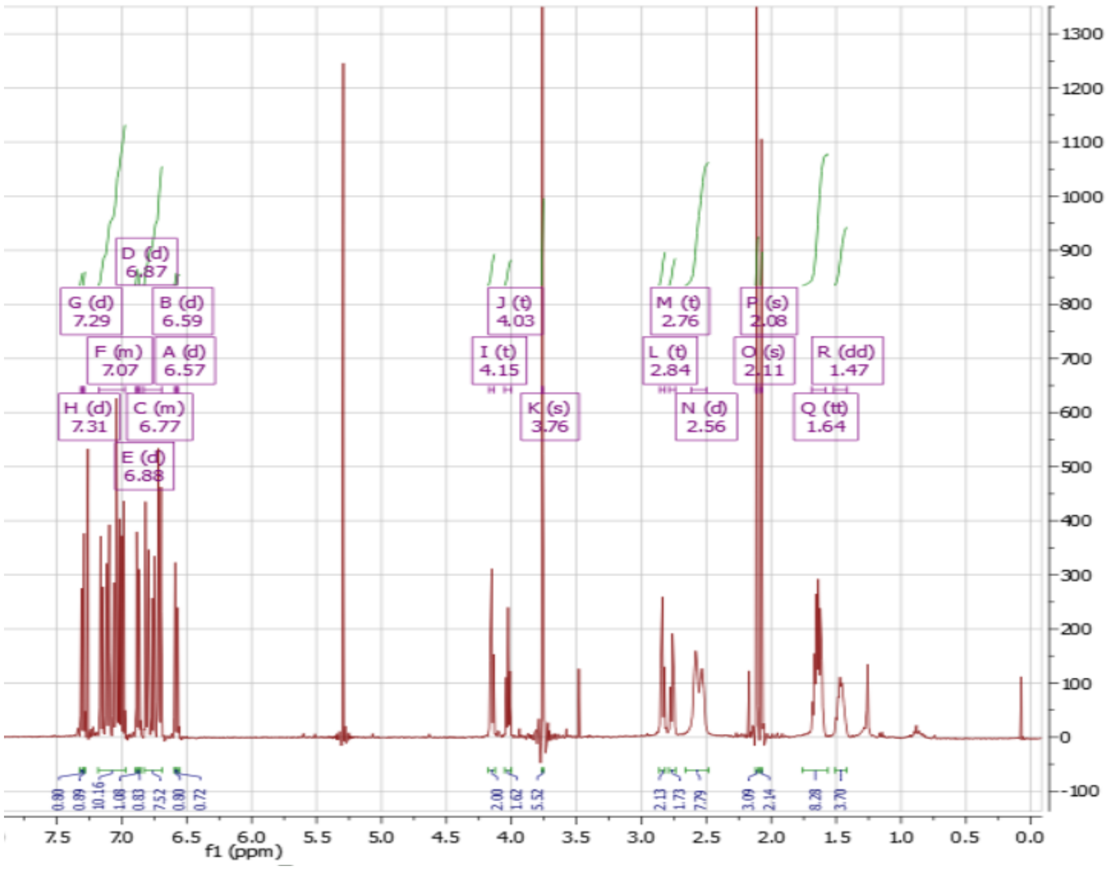

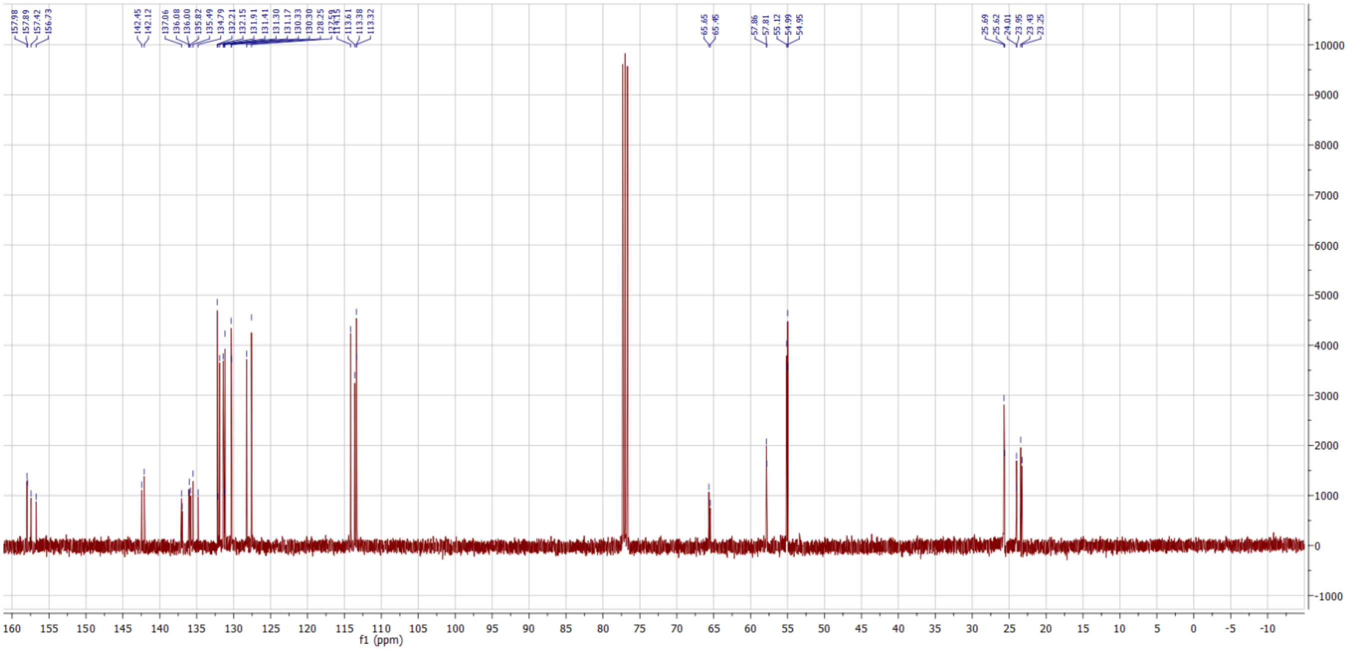

***E*/*Z*-4-(2-{4-[1-(4-Chloro-phenyl)-2-(4-methoxy-phenyl)-propenyl]-phenoxy}-ethyl)-morpholine (13).**

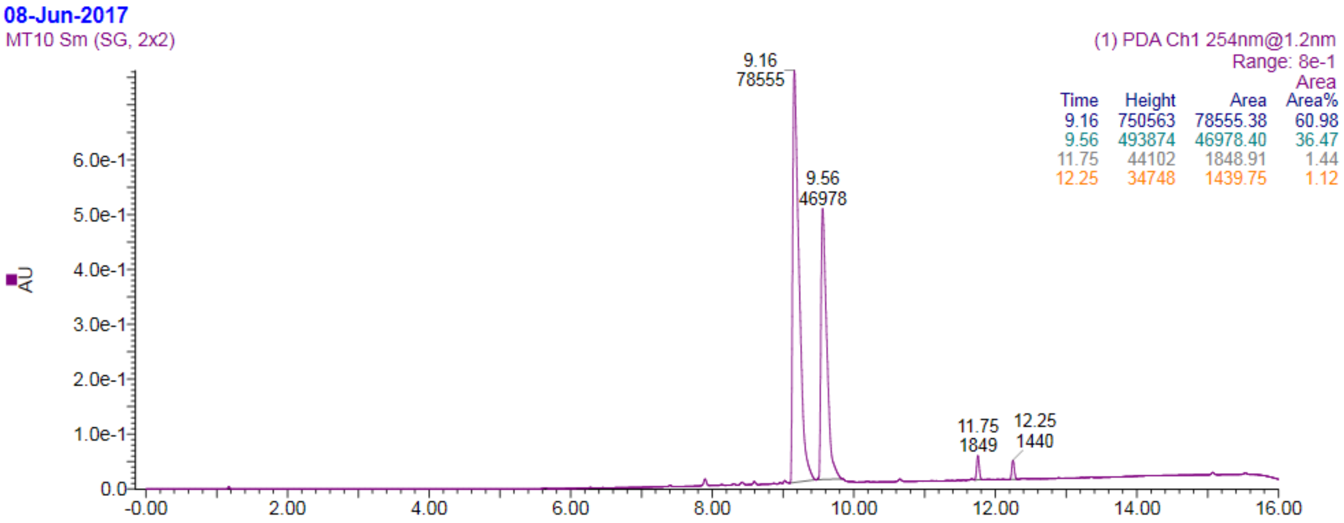

08-Jun-2017
MT10 Sm (SG, 2x2)
(1) PDA Ch1 254nm@1.2nm
Range: 8e-1
Area
Time Height Area Area%
9.16 750563 78555.38 60.98
9.56 493874 46978.40 36.47
11.75 44102 1848.91 1.44
12.25 34748 1439.75 1.12
9.16
78555
9.56
46978
11.75
1849
12.25
1440
AU
6.0e-1
5.0e-1
4.0e-1
3.0e-1
2.0e-1
1.0e-1
0.0
-0.00
2.00
4.00
6.00
8.00
10.00
12.00
14.00
16.00

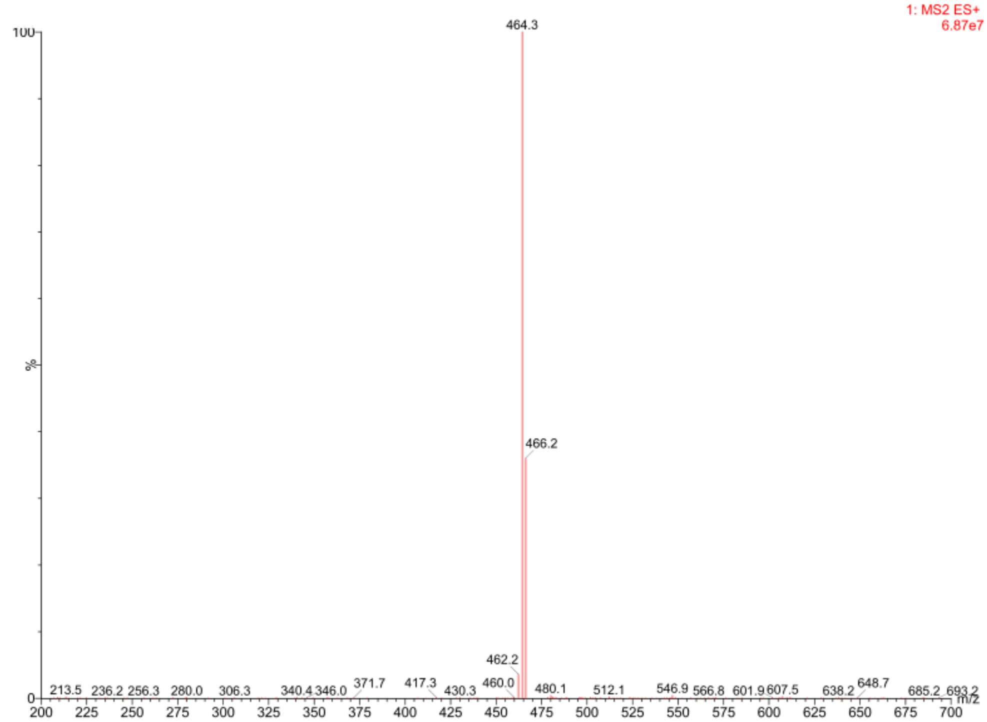

1: MS2 ES+
6.87e7
464.3
466.2
462.2
460.0
213.5 236.2 256.3 280.0 306.3 340.4 346.0 371.7 417.3 430.3 480.1 512.1 546.9 566.8 601.9 607.5 638.2 648.7 685.2 693.2
100
%
0
200 225 250 275 300 325 350 375 400 425 450 475 500 525 550 575 600 625 650 675 700
m/z

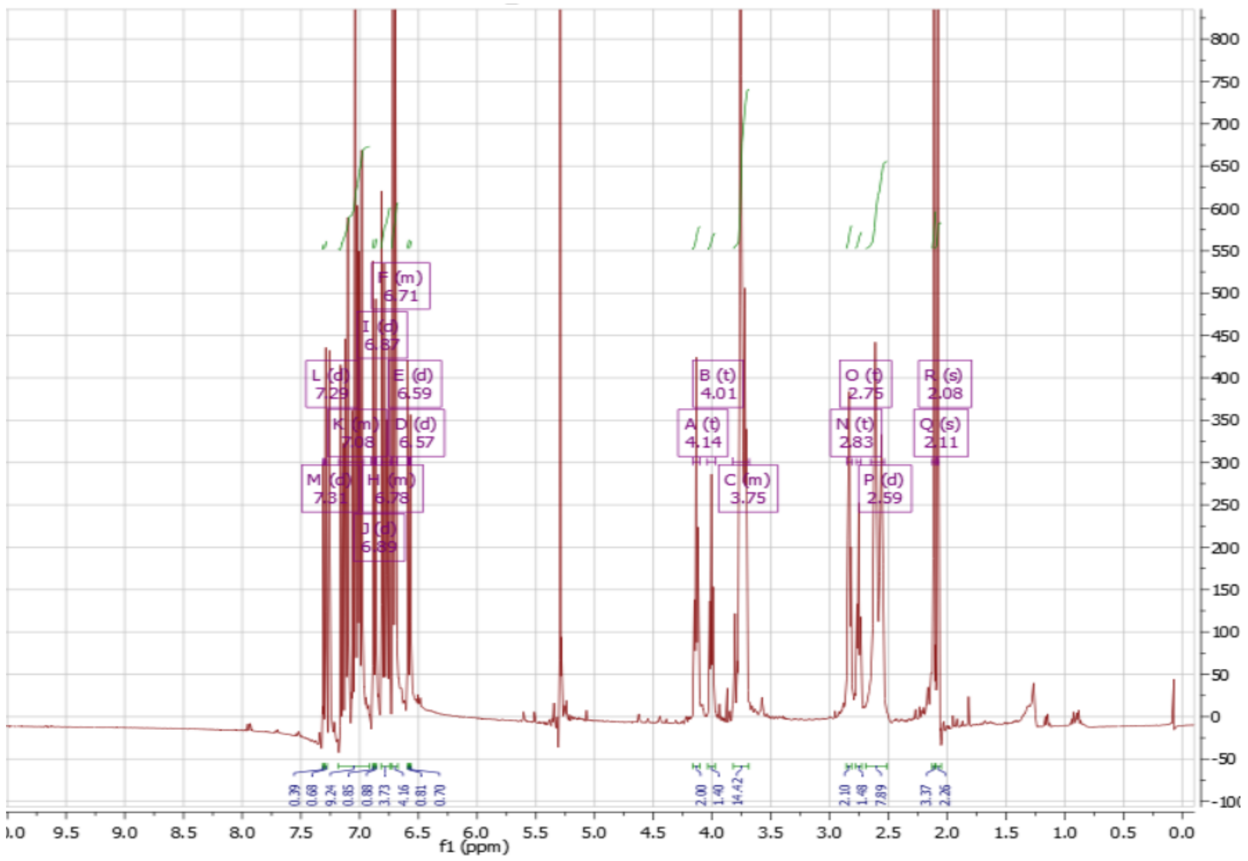

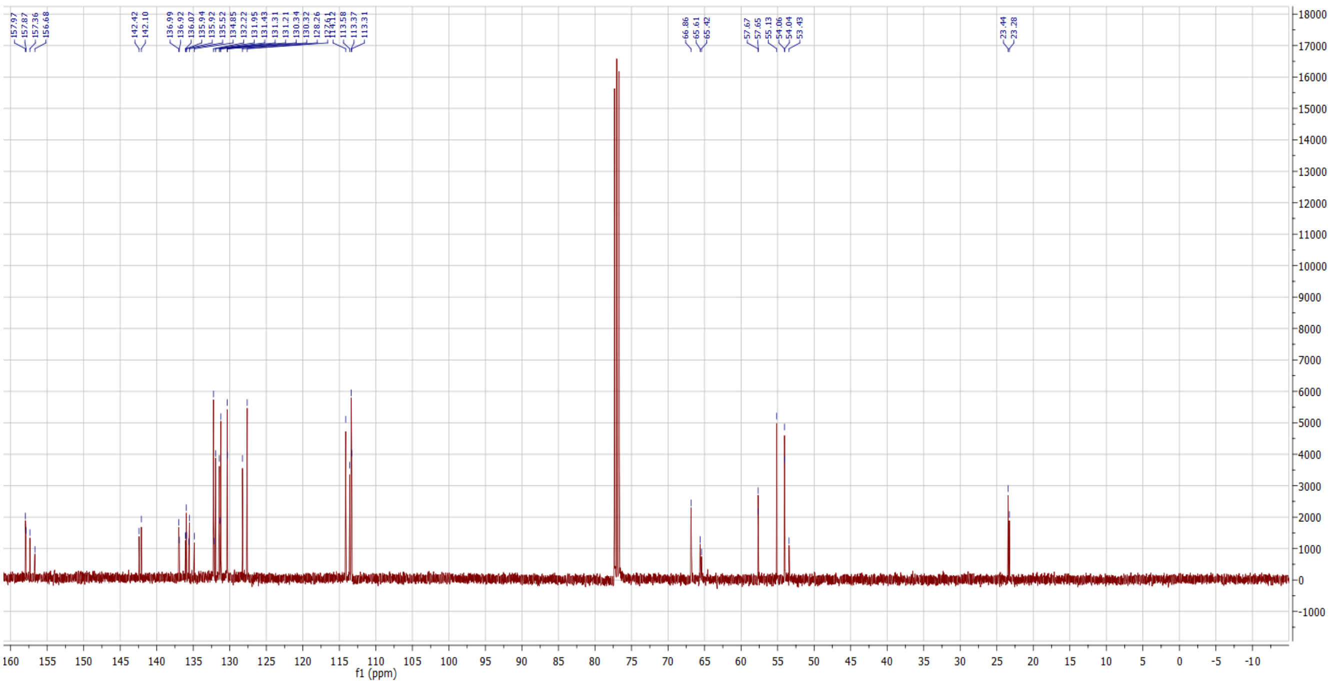

***E*/*Z*-(3-{4-[1-(4-Chlorophenyl)-2-(3-fluoro-4-methoxyphenyl)-propenyl]-phenoxy}-propyl)-dimethyl-amine (15)**

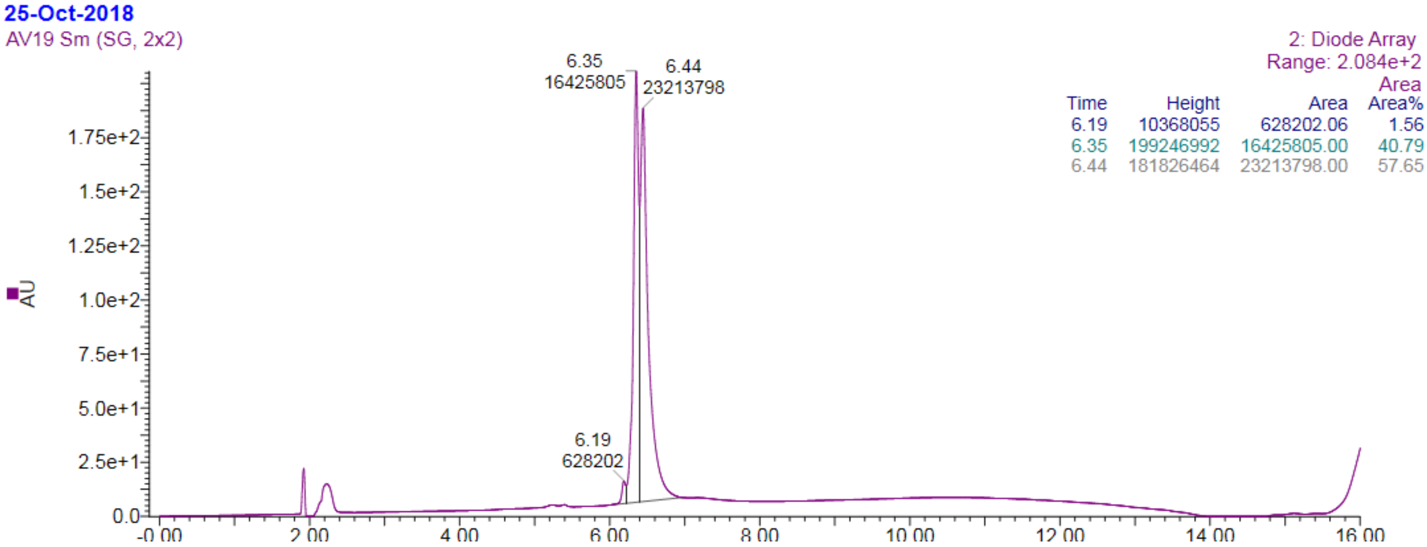

Time	Height	Area	Area%
6.19	10368055	628202.06	1.56
6.35	199246992	16425805.00	40.79
6.44	181826464	23213798.00	57.65

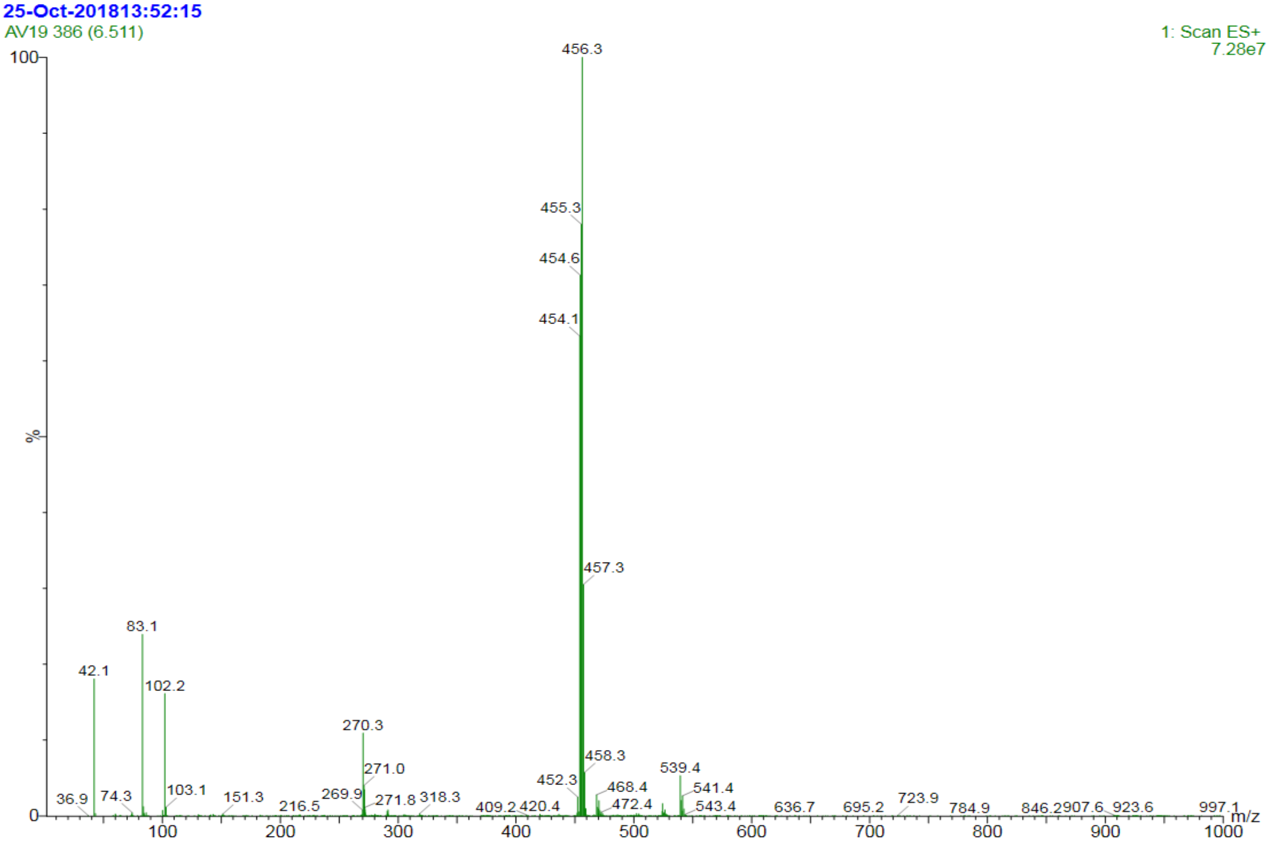

25-Oct-201813:52:15
AV19 386 (6.511)
1: Scan ES+
7.28e7
456.3
455.3
454.6
454.1
457.3
83.1
42.1
102.2
270.3
458.3
271.0
539.4
452.3
468.4
541.4
472.4
543.4
36.9
74.3
103.1
151.3
216.5
269.9
271.8
318.3
409.2
420.4
636.7
695.2
723.9
784.9
846.2
907.6
923.6
997.1
m/z
%

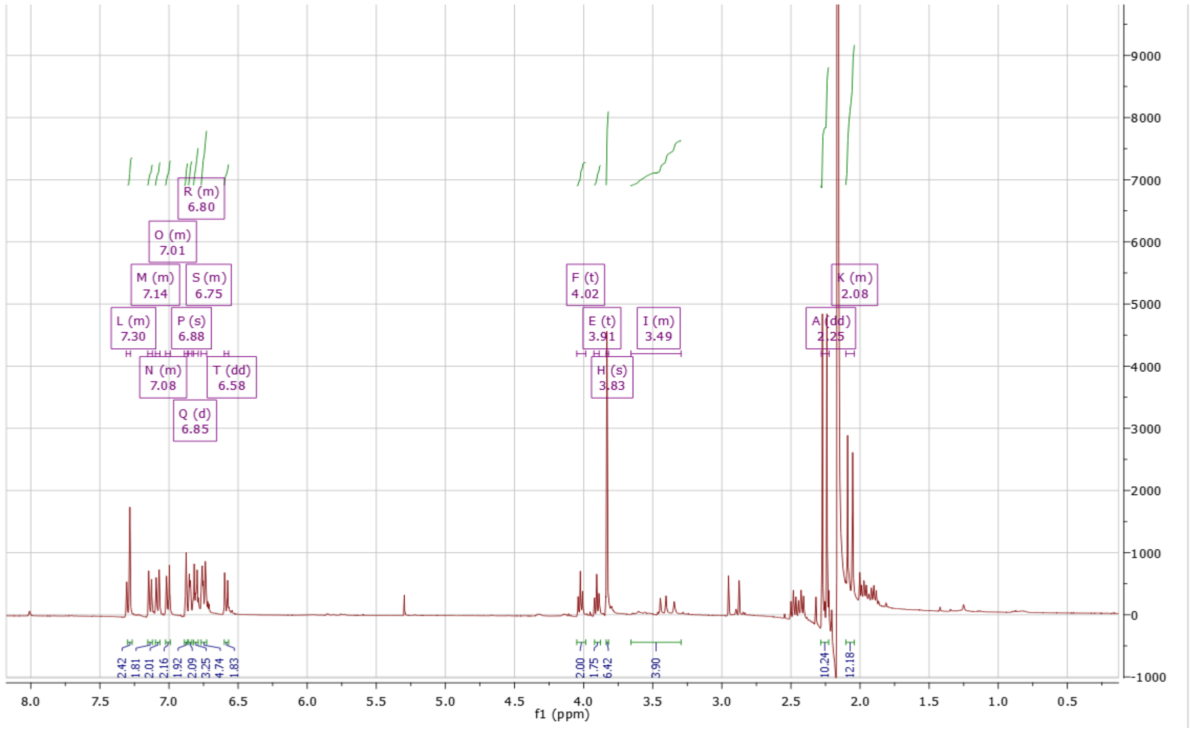

R (m) 6.80
O (m) 7.01
M (m) 7.14
S (m) 6.75
L (m) 7.30
P (s) 6.88
N (m) 7.08
T (dd) 6.58
Q (d) 6.85
F (t) 4.02
E (t) 3.91
I (m) 3.49
H (s) 3.83
K (m) 2.08
A (dd) 2.25
2.42
1.81
2.01
2.16
1.92
2.09
3.25
4.74
1.83
2.00
1.75
6.42
3.90
10.24
12.18
f1 (ppm)

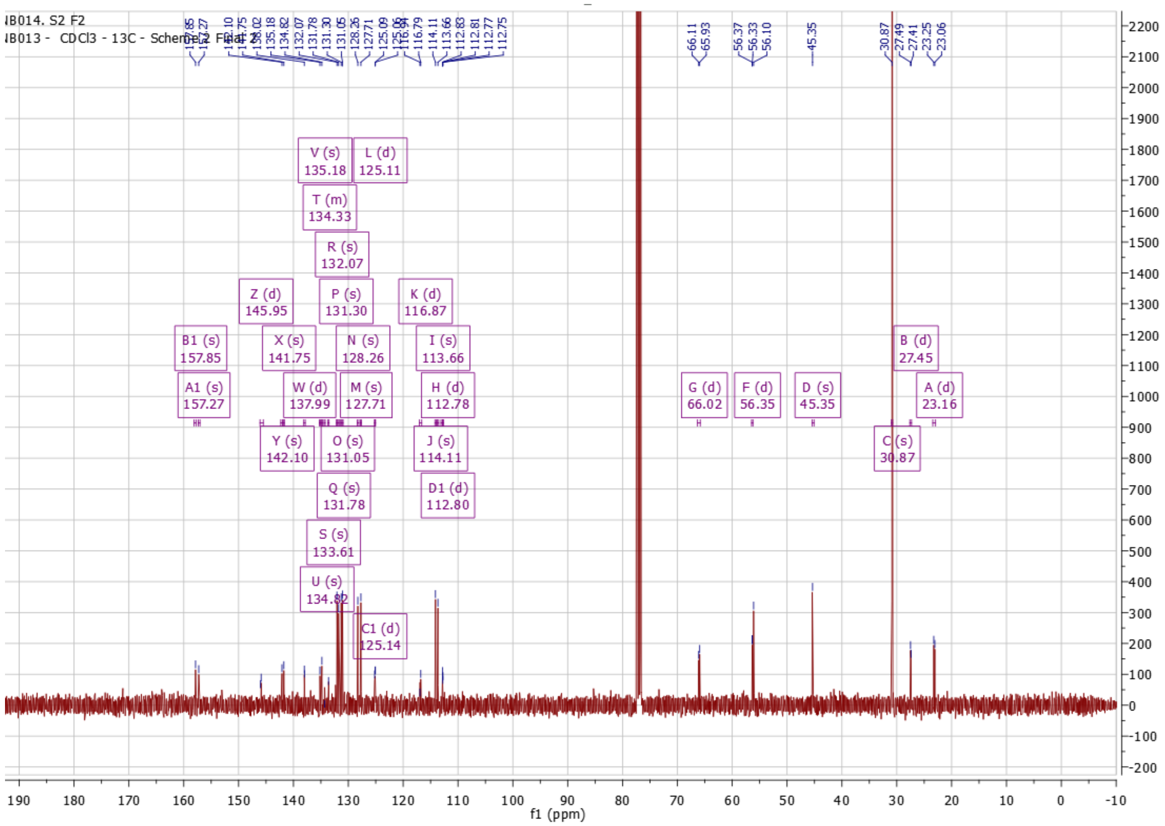

NB014. S2 F2
NB013 - CDCl3 - 13C - Scheme 2 Final 2
f1 (ppm)

***E/Z*-1-(2-{4-[1-(4-Chlorophenyl)-2-(3-fluoro-4-methoxyphenyl)-propenyl]-phenoxy}ethyl)-pyrrolidine (16)**

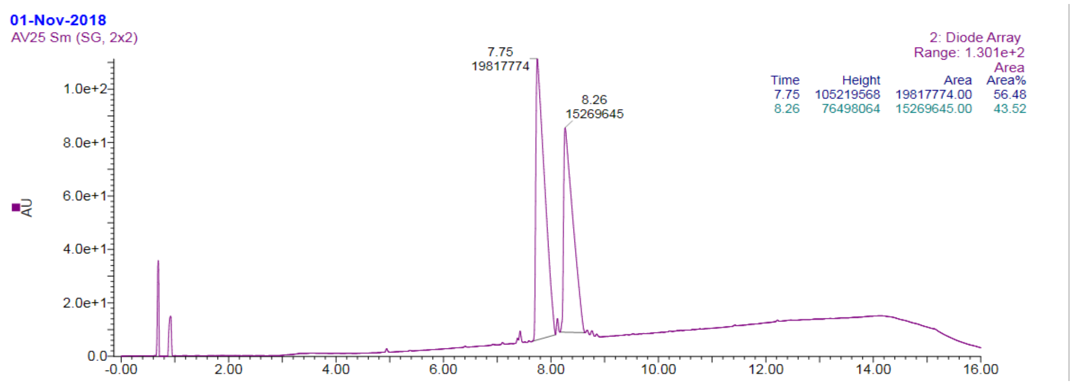

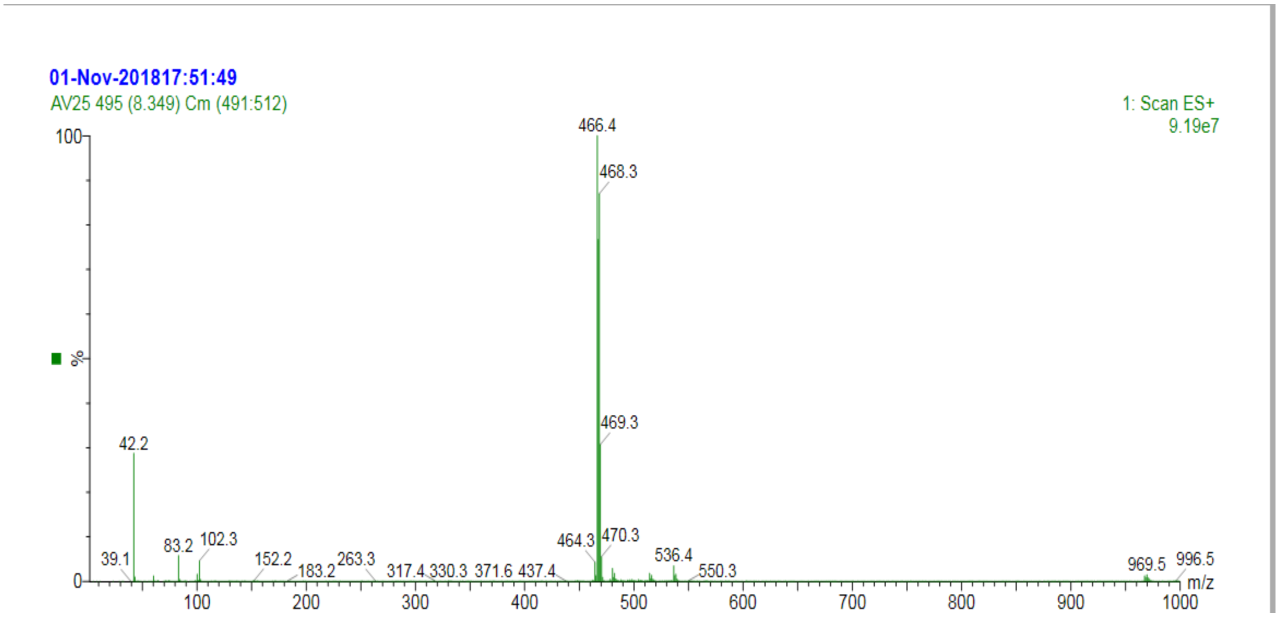

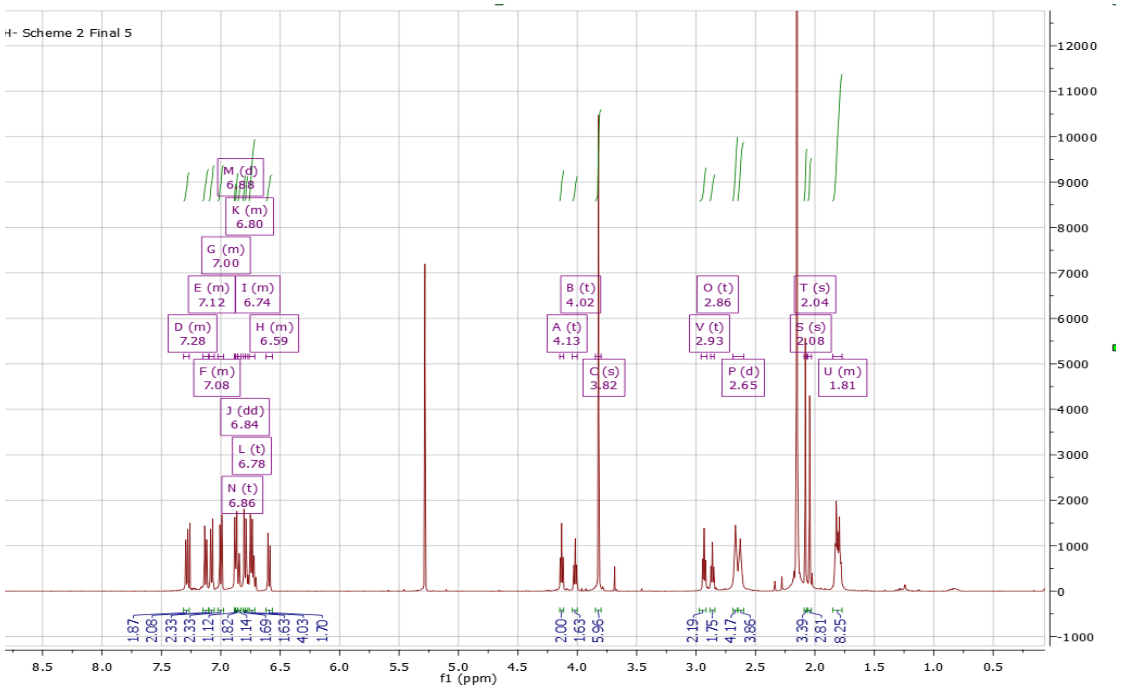
H- Scheme 2 Final 5
f1 (ppm)

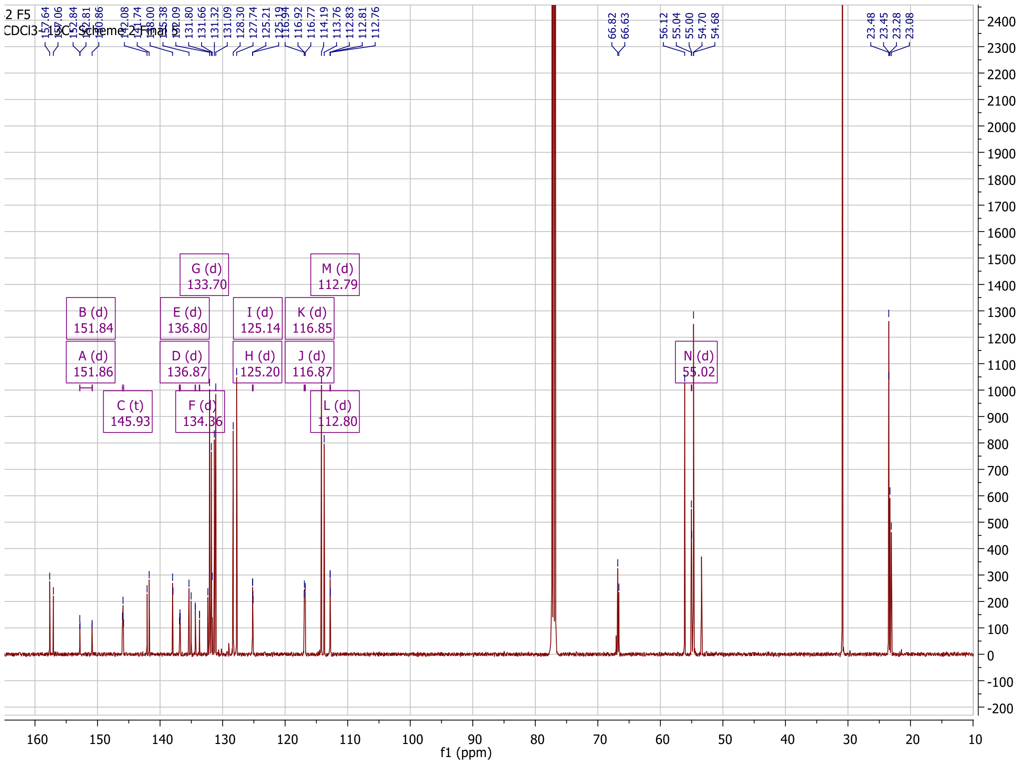
2 F5
CDCl3-13C-Scheme 2 Final 5
f1 (ppm)

E/Z-1-(2-{4-[1-(4-Chlorophenyl)-2-(3-fluoro-4-methoxyphenyl)-propenyl]-phenoxy}ethyl)-piperidine (17)

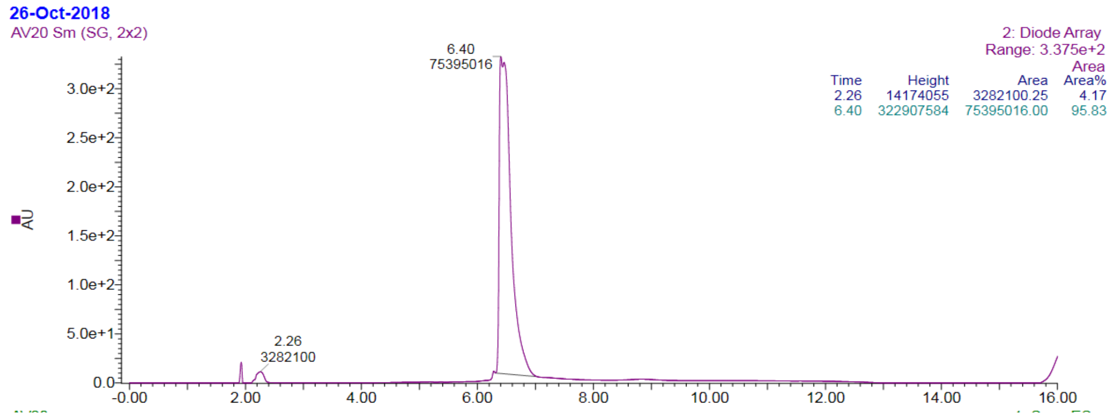

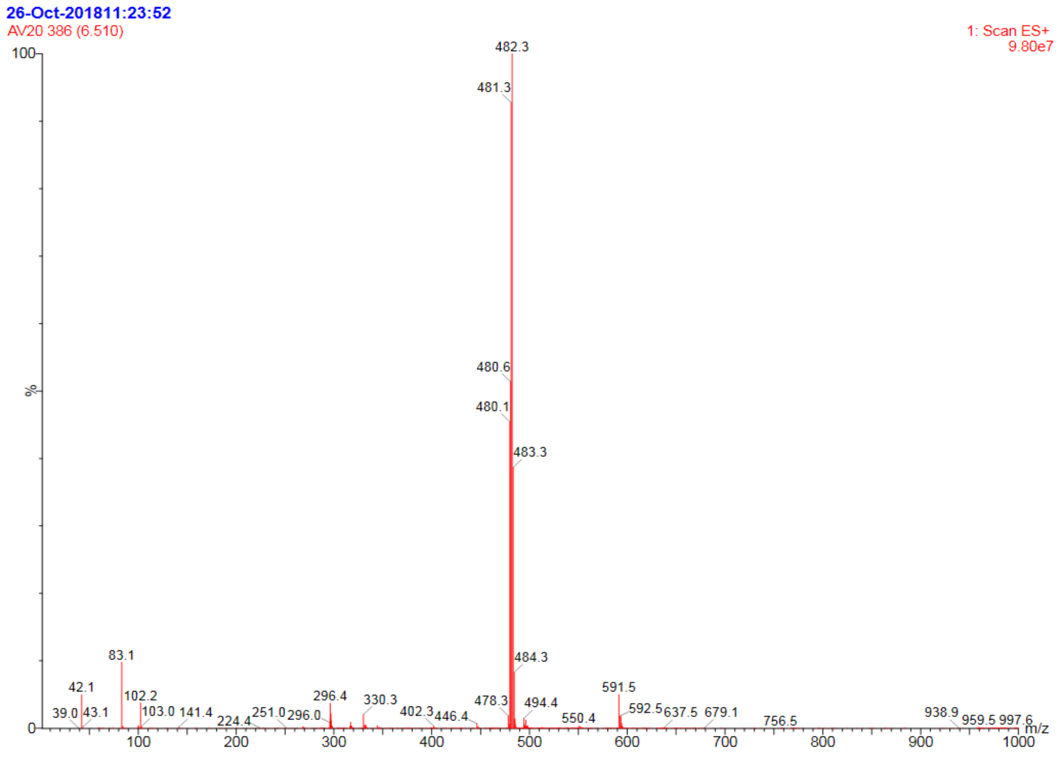

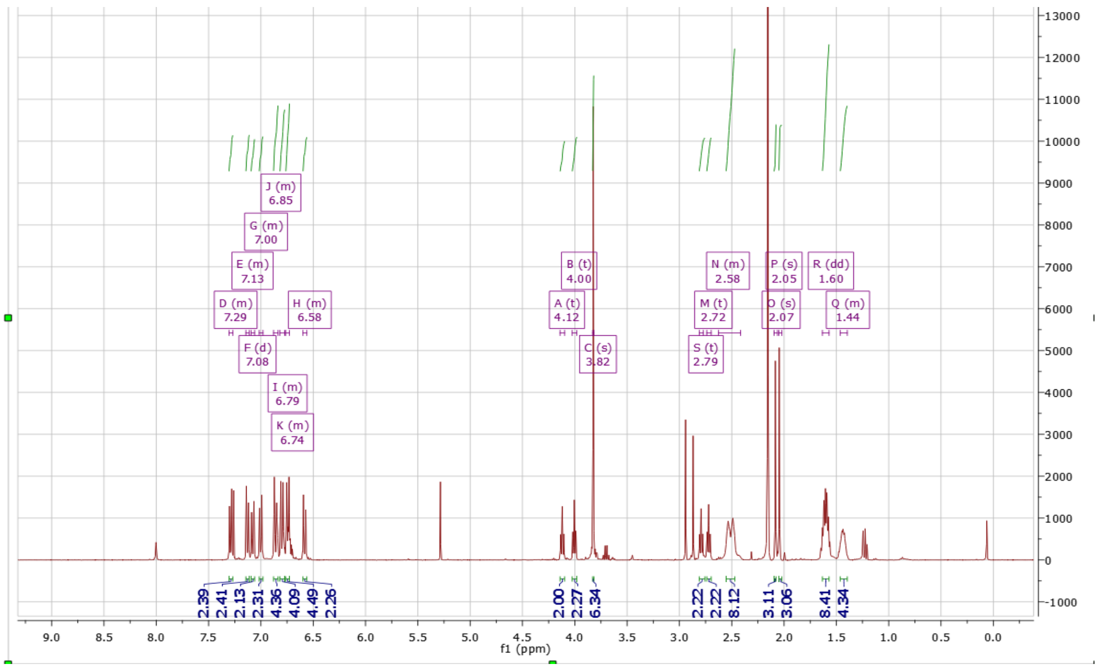

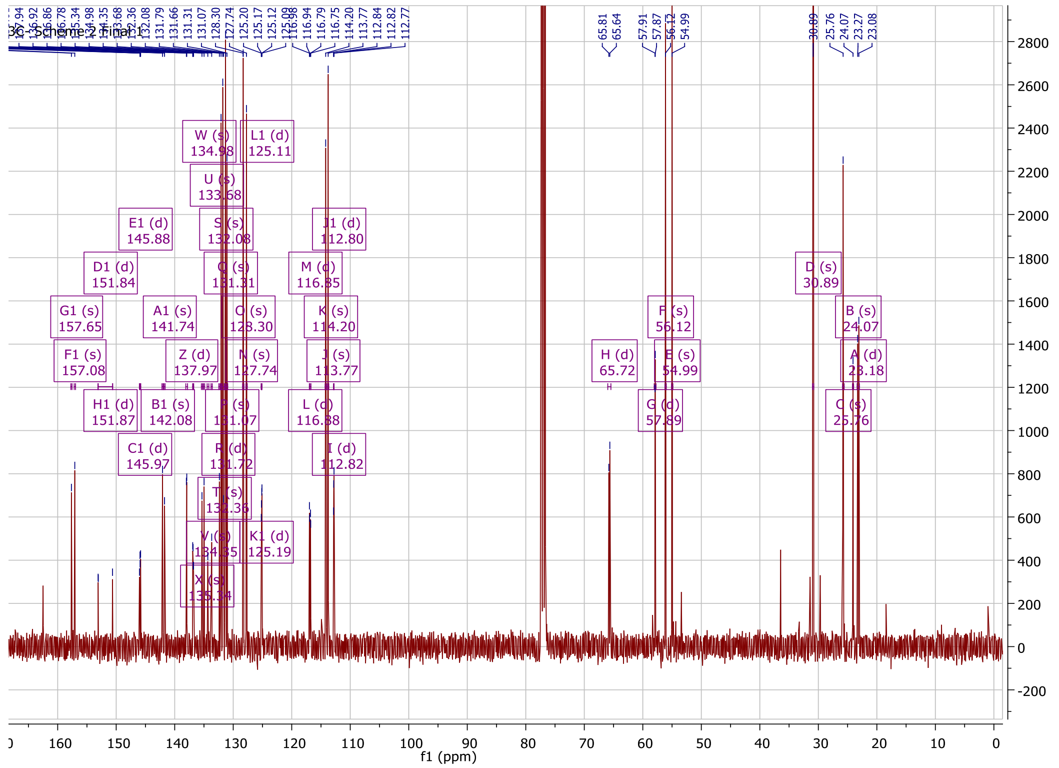

***E/Z*-4-(2-{4-[1-(4-Chlorophenyl)-2-(3-fluoro-4-methoxyphenyl)-propenyl]-phenoxy}ethyl)-morpholine (18)**

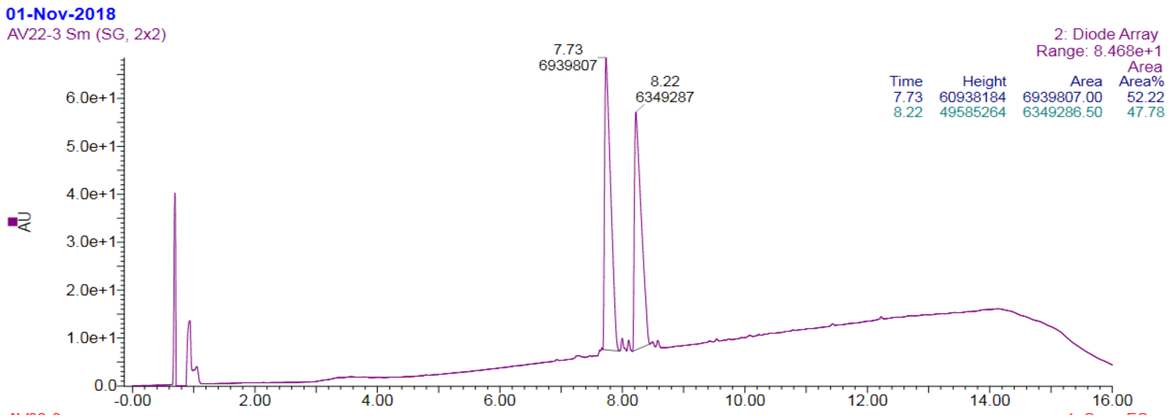

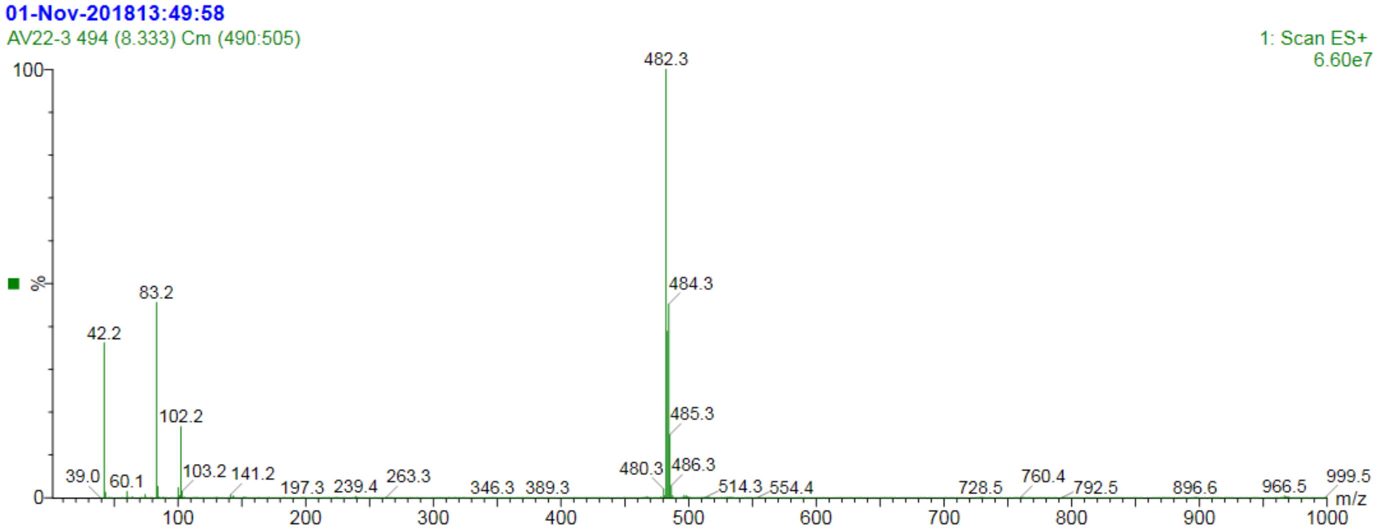

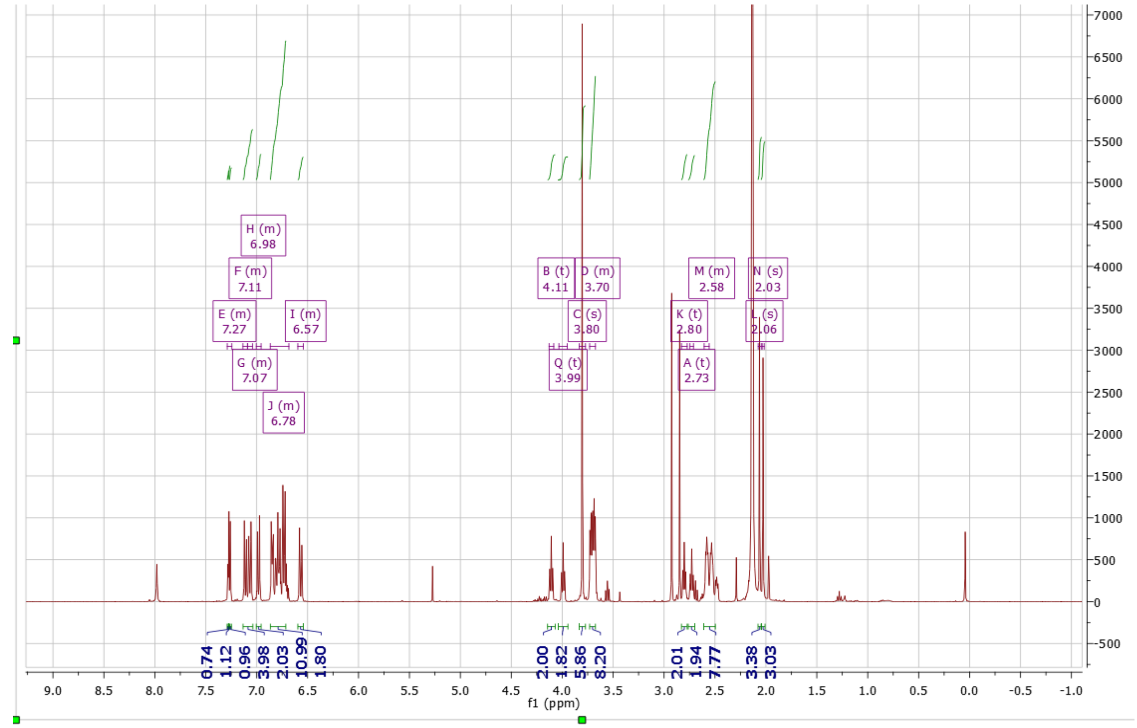
H (m)
6.98
F (m)
7.11
E (m)
7.27
I (m)
6.57
G (m)
7.07
J (m)
6.78
B (t)
4.11
D (m)
3.70
C (s)
3.80
Q (t)
3.99
M (m)
2.58
N (s)
2.03
K (t)
2.80
L (s)
2.06
A (t)
2.73
f1 (ppm)

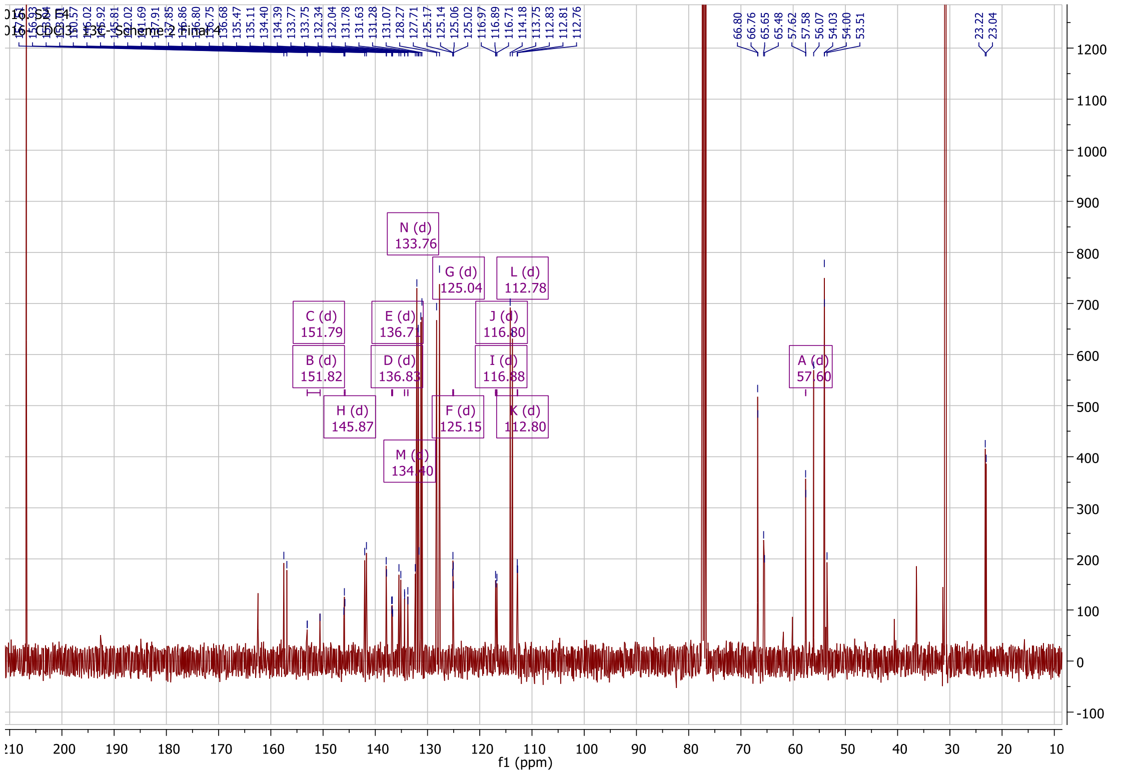
N (d)
133.76
G (d)
125.04
L (d)
112.78
C (d)
151.79
E (d)
136.71
J (d)
116.80
B (d)
151.82
D (d)
136.83
I (d)
116.88
A (d)
57.60
H (d)
145.87
F (d)
125.15
K (d)
112.80
M (d)
134.40
f1 (ppm)

***E/Z*-(2-{4-[1-(4-Chlorophenyl)-2-(3-fluoro-4-methoxyphenyl)-propenyl]-phenoxy}-ethyl)-dimethyl-**

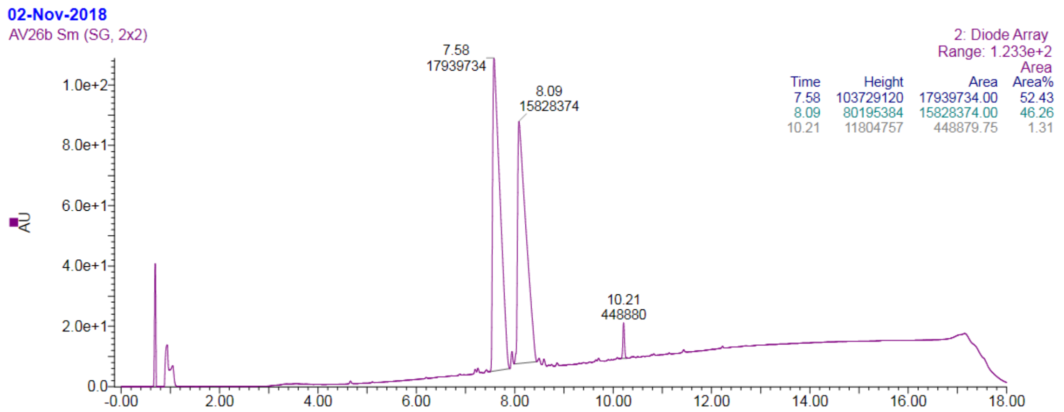

amine (20)

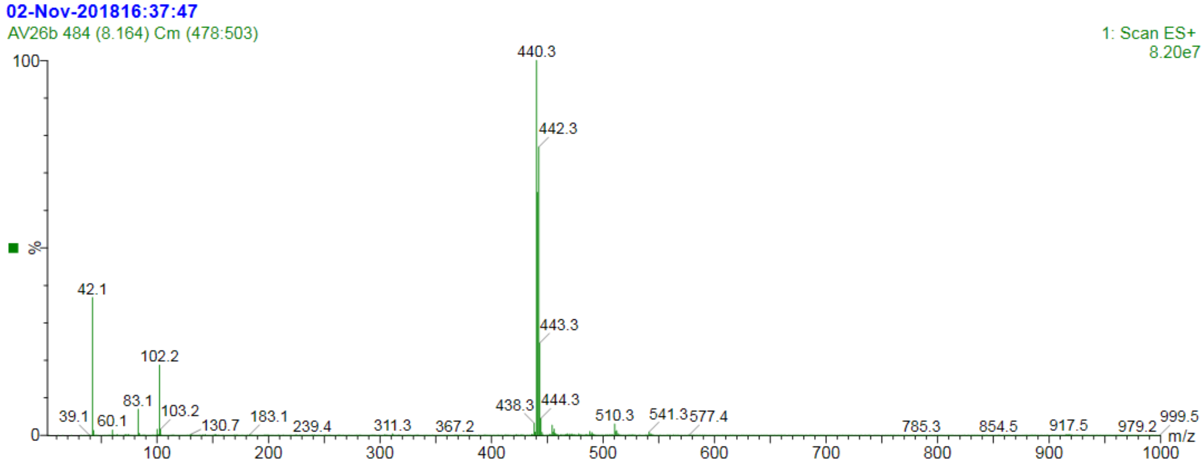

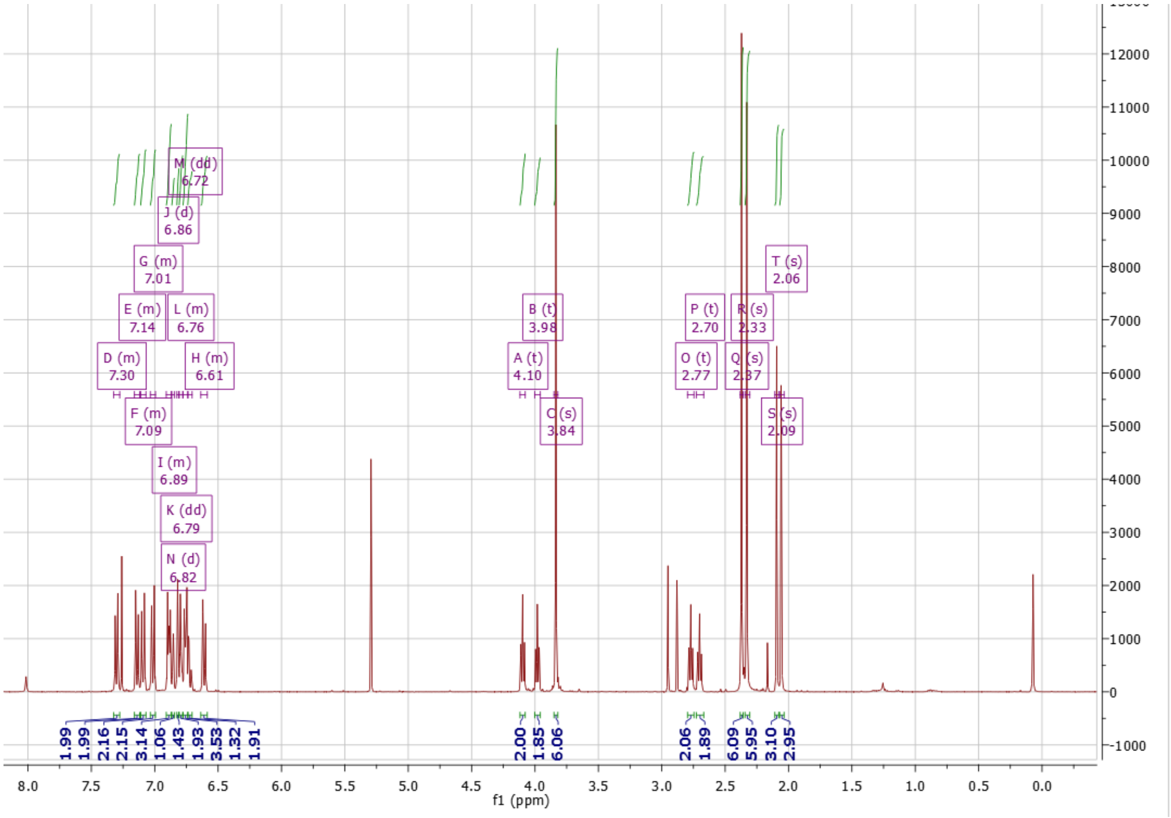
M (dd) 6.72
J (d) 6.86
G (m) 7.01
E (m) 7.14
L (m) 6.76
D (m) 7.30
H (m) 6.61
F (m) 7.09
I (m) 6.89
K (dd) 6.79
N (d) 6.82
B (t) 3.98
A (t) 4.10
C (s) 3.84
P (t) 2.70
R (s) 2.33
O (t) 2.77
Q (s) 2.37
T (s) 2.06
S (s) 2.09
1.99 1.99 2.16 2.15 3.14 1.06 1.43 1.93 3.53 1.32 1.91
2.00 1.85 6.06
2.06 1.89 6.09 5.95 3.10 2.95
f1 (ppm)

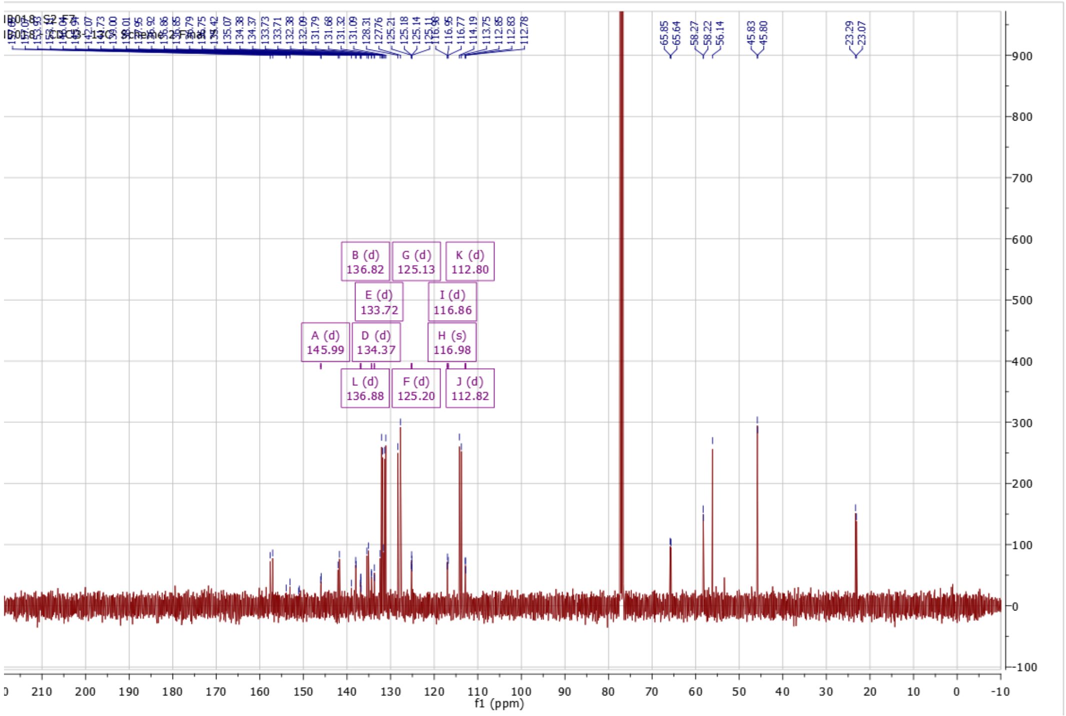
B (d) 136.82
G (d) 125.13
K (d) 112.80
E (d) 133.72
I (d) 116.86
A (d) 145.99
D (d) 134.37
H (s) 116.98
L (d) 136.88
F (d) 125.20
J (d) 112.82
65.85 65.64 58.27 58.22 56.14 45.83 45.80 23.29 23.07
f1 (ppm)

***E*/*Z*-(2-{4-[1-(4-Chlorophenyl)-2-(3-fluoro-4-methoxyphenyl)-propenyl]-phenoxy}ethyl)-diethyl-amine (21)**

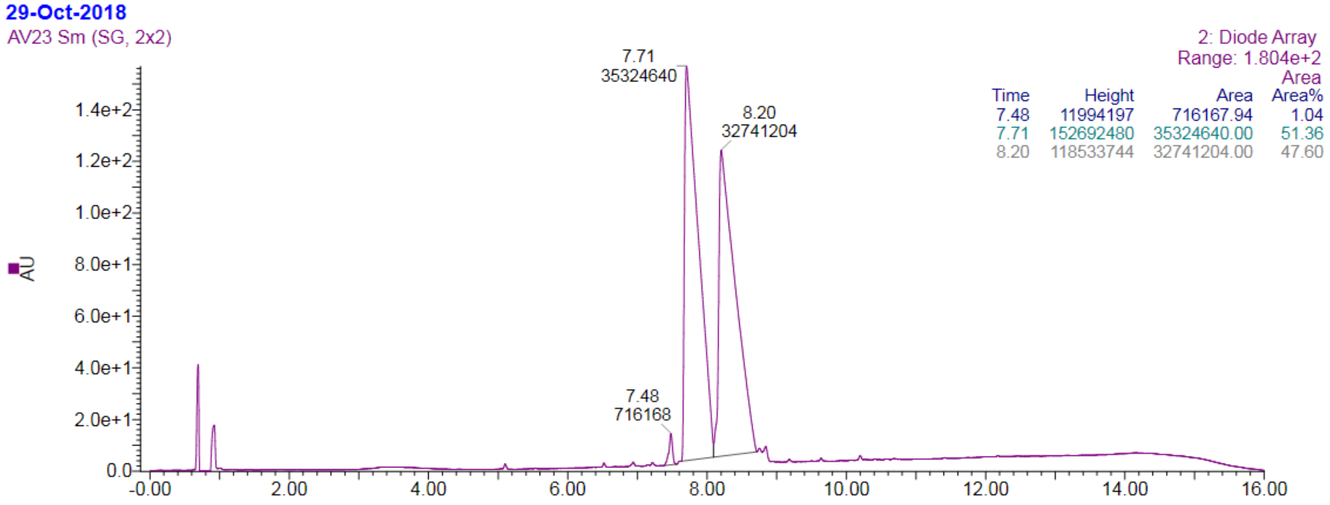

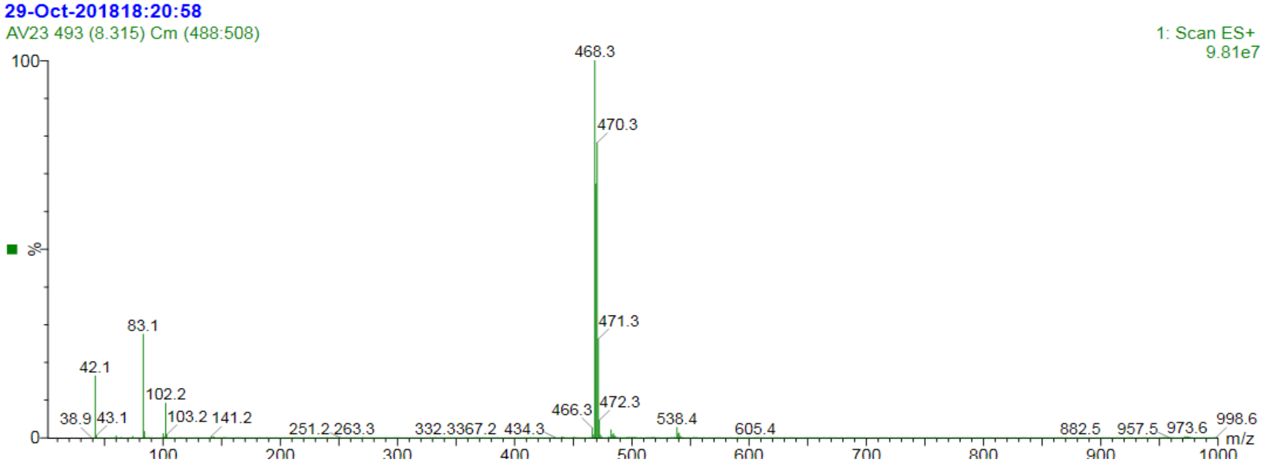

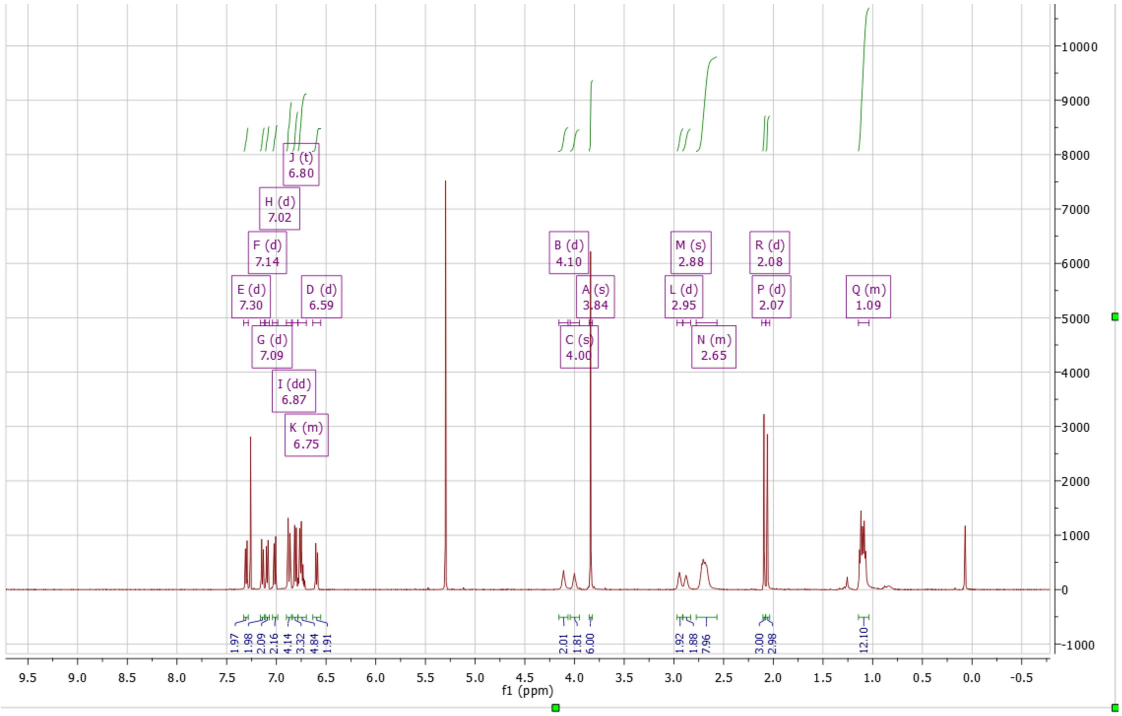

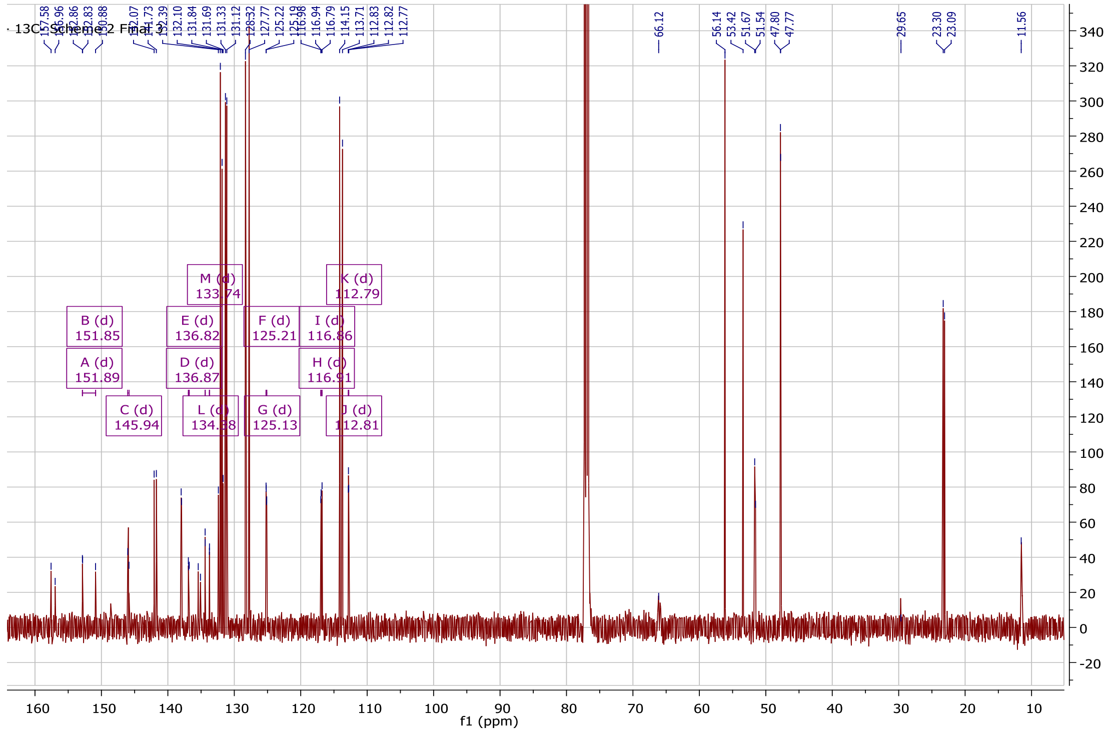

***E/Z*-(3-{4-[1-(4-Chlorophenyl)-2-(4-fluoro-3-methoxyphenyl)-propenyl]-phenoxy}propyl)-dimethyl-amine (22)**

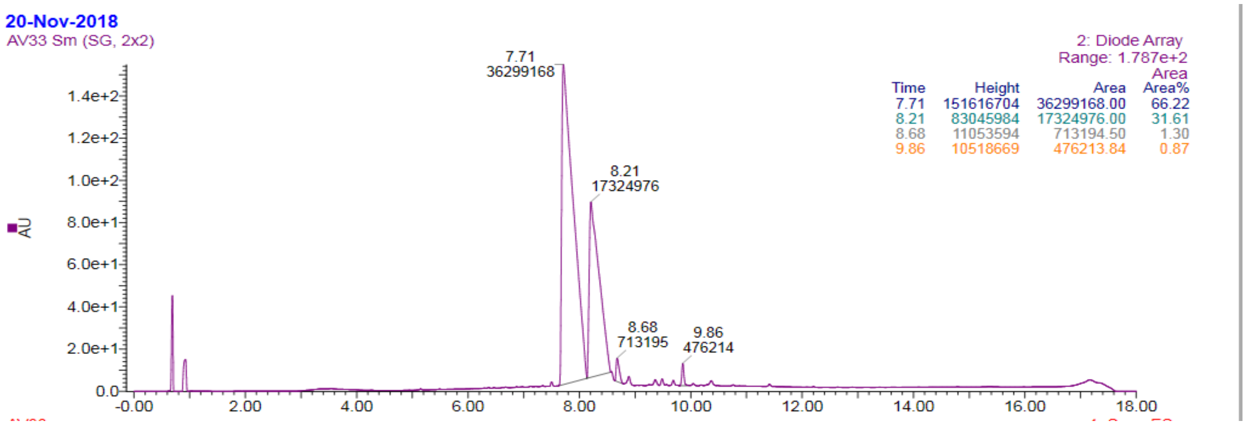
20-Nov-2018
AV33 Sm (SG, 2x2)
2: Diode Array
Range: 1.787e+2
Time Height Area Area%
7.71 151616704 36299168.00 66.22
8.21 83045984 17324976.00 31.61
8.68 11053594 713194.50 1.30
9.86 10518669 476213.84 0.87
7.71
36299168
8.21
17324976
8.68
713195
9.86
476214
AU

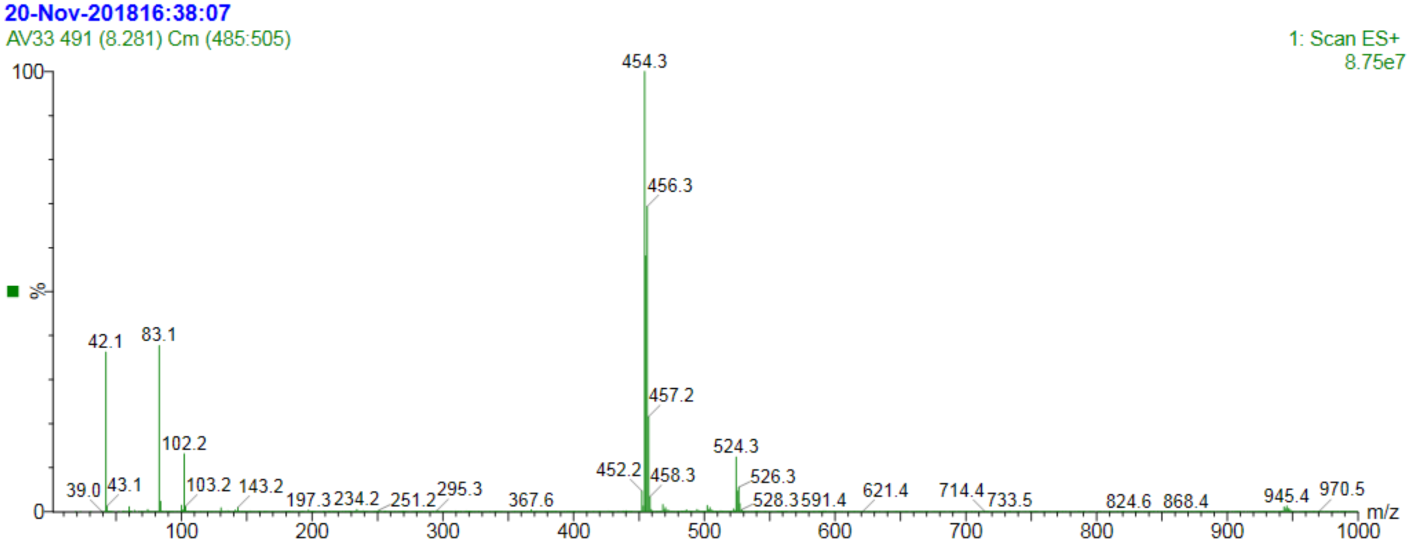
20-Nov-201816:38:07
AV33 491 (8.281) Cm (485:505)
1: Scan ES+
8.75e7
454.3
456.3
457.2
524.3
526.3
452.2
458.3
42.1
83.1
102.2
39.0
43.1
103.2
143.2
197.3
234.2
251.2
295.3
367.6
528.3
591.4
621.4
714.4
733.5
824.6
868.4
945.4
970.5
%
m/z

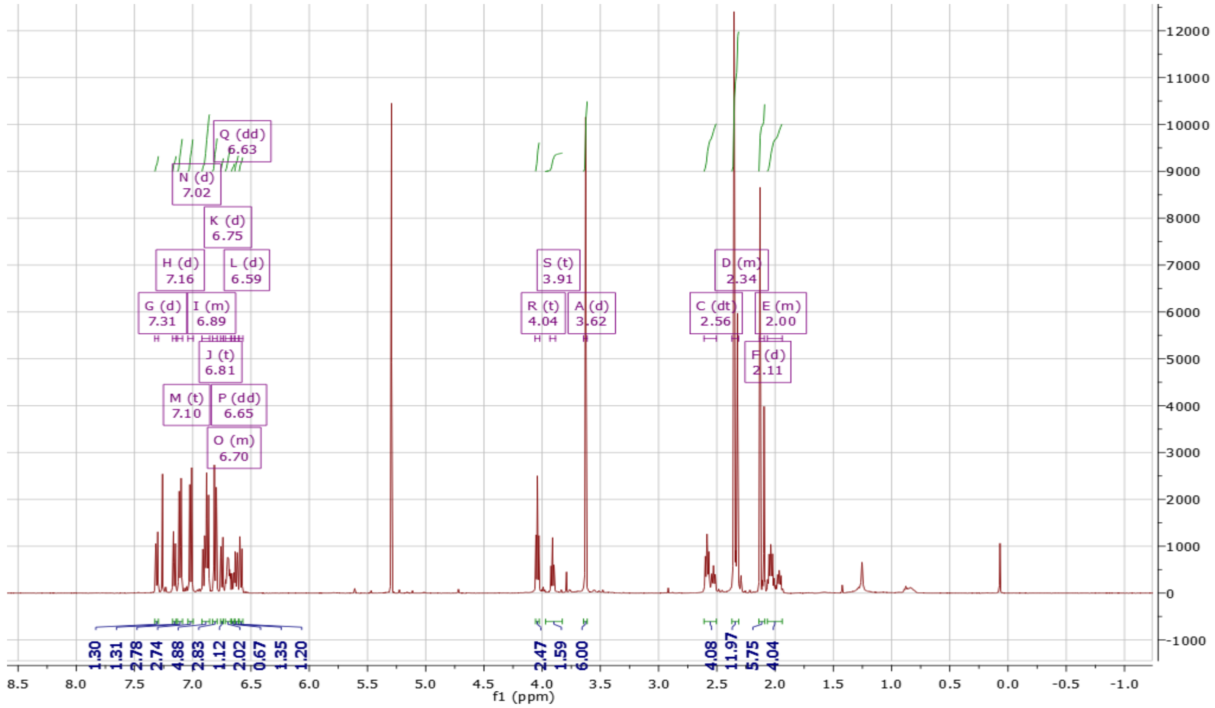

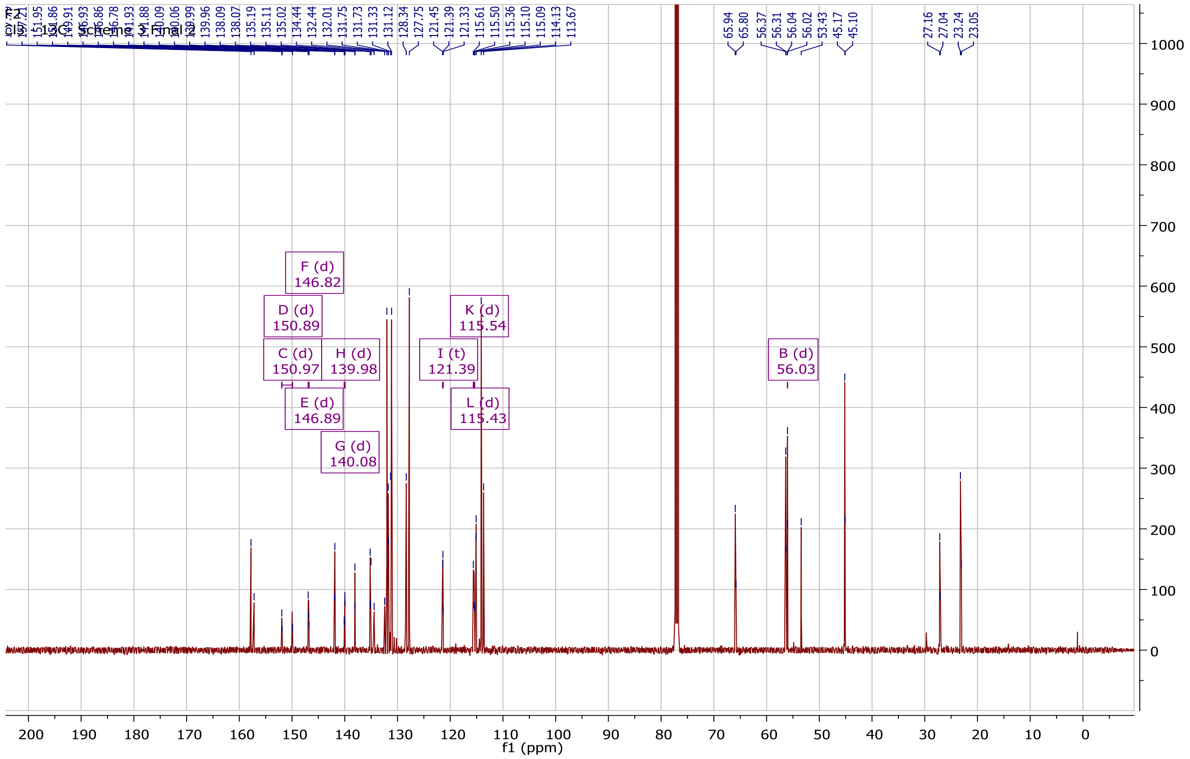

E/Z **-1-(2-{4-[1-(4-Chlorophenyl)-2-(4-fluoro-3-methoxyphenyl)-propenyl]-phenoxy}-ethyl)-pyrrolidine (23)**

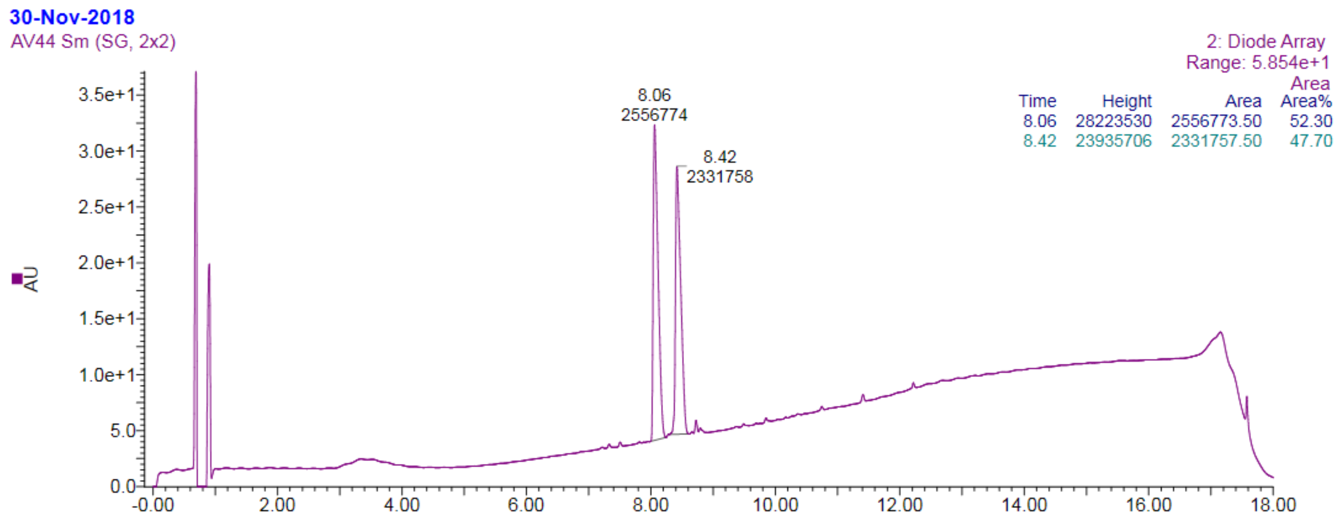

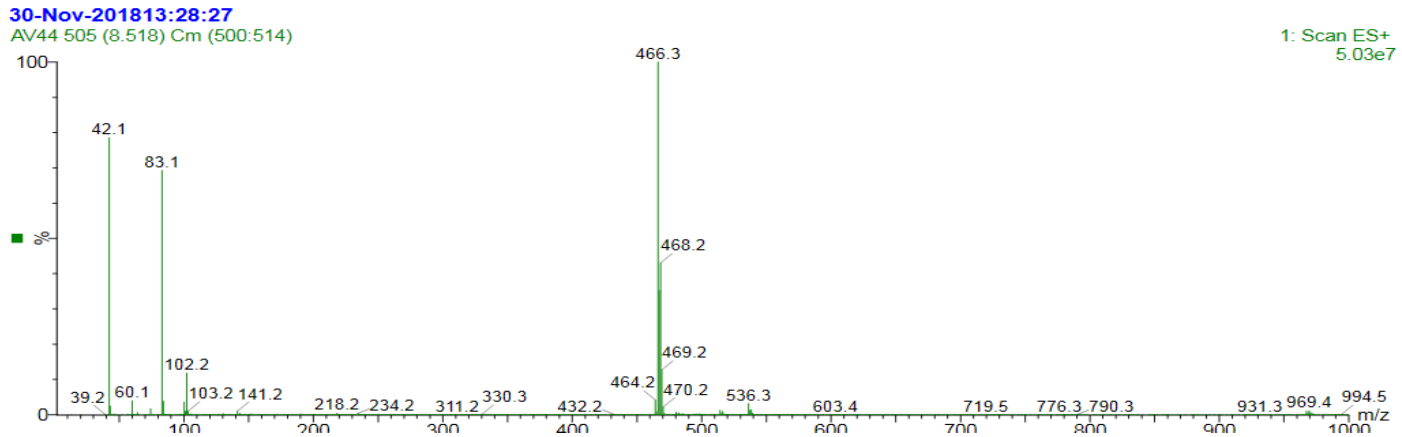

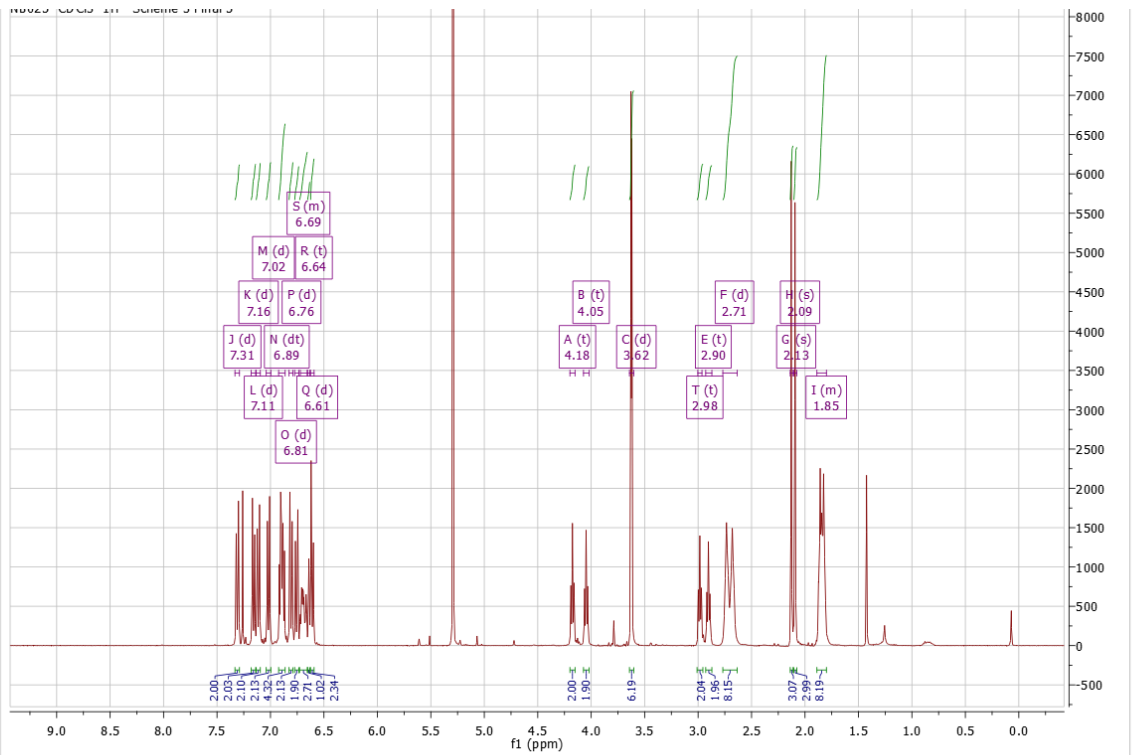
S (m) 6.69
M (d) 7.02
R (t) 6.64
K (d) 7.16
P (d) 6.76
J (d) 7.31
N (dt) 6.89
L (d) 7.11
Q (d) 6.61
O (d) 6.81
B (t) 4.05
A (t) 4.18
C (d) 3.62
F (d) 2.71
E (t) 2.90
T (t) 2.98
H (s) 2.09
G (s) 2.13
I (m) 1.85
f1 (ppm)

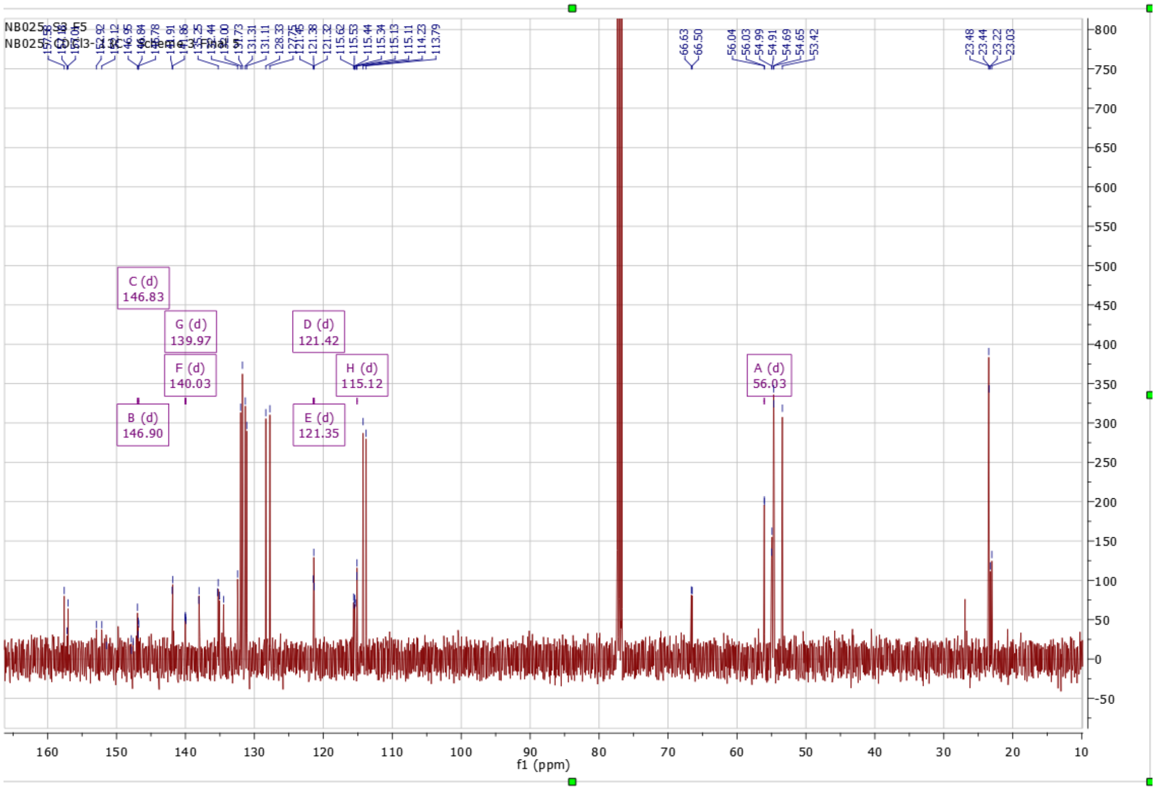
C (d) 146.83
G (d) 139.97
F (d) 140.03
B (d) 146.90
D (d) 121.42
H (d) 115.12
E (d) 121.35
A (d) 56.03
f1 (ppm)

***E/Z*-4-(2-{4-[1-(4-Chlorophenyl)-2-(4-fluoro-3-methoxyphenyl)-propenyl]-phenoxy}ethyl)-morpholine (25)**

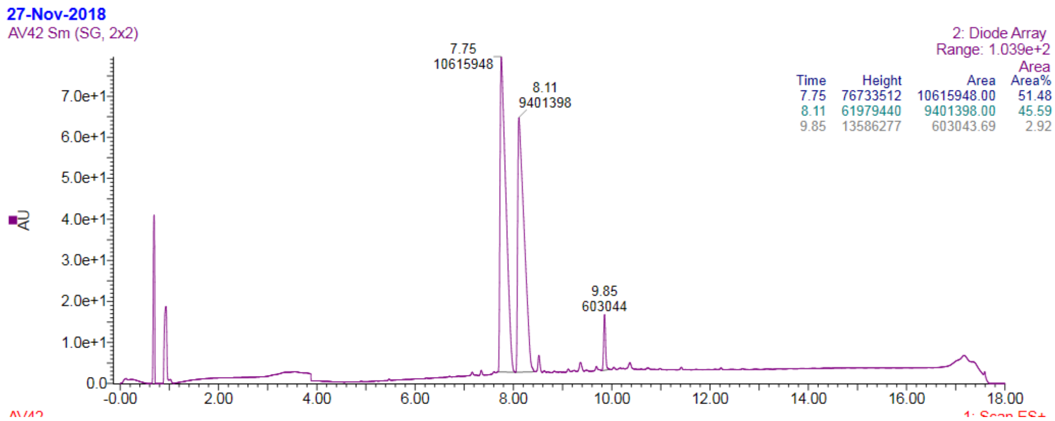

Time	Height	Area	Area%
7.75	76733512	10615948.00	51.48
8.11	61979440	9401398.00	45.59
9.85	13586277	603043.69	2.92

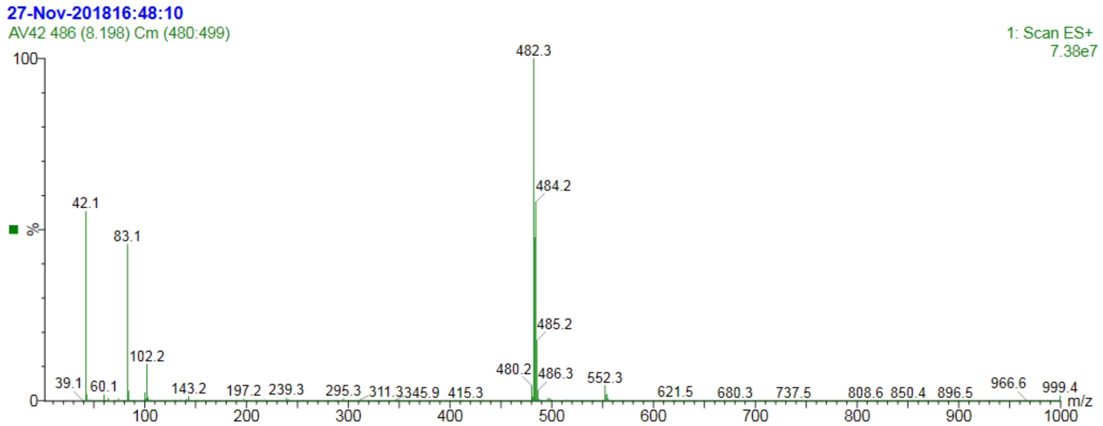

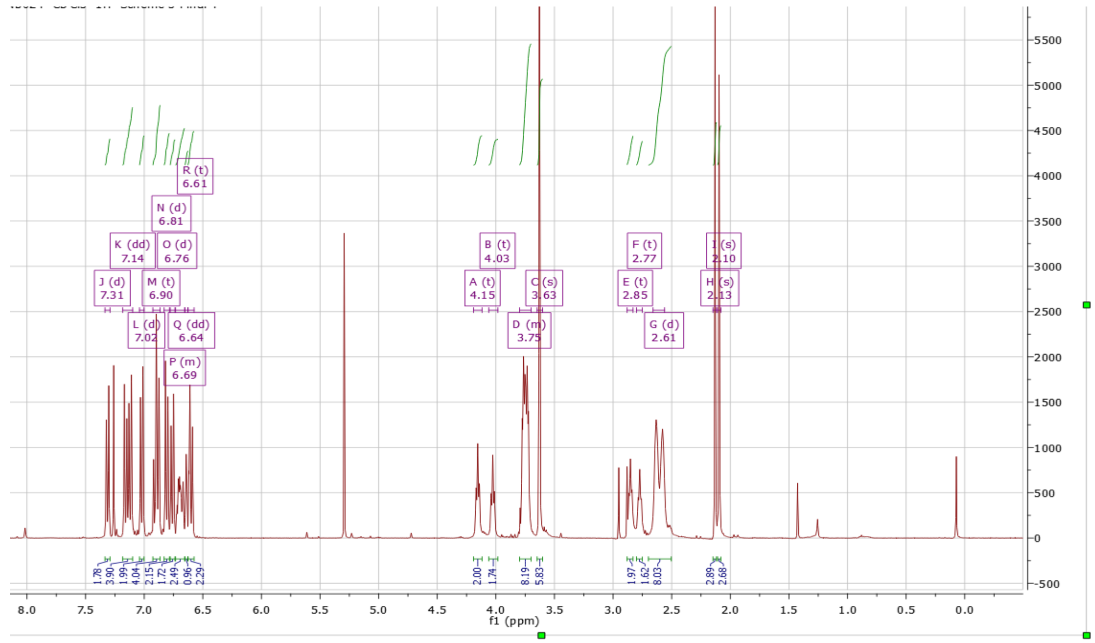

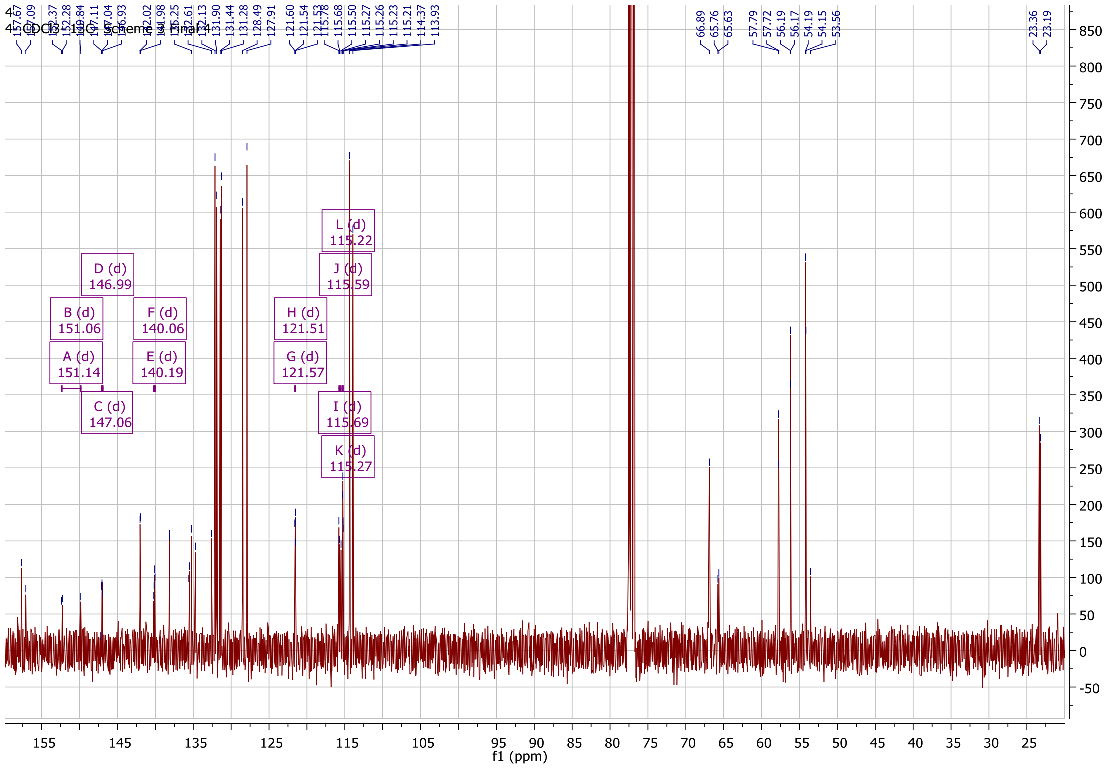

***E*/*Z*-(2-{4-[1-(4-Chlorophenyl)-2-(4-fluoro-3-methoxyphenyl)-propenyl]-phenoxy}ethyl)-dimethyl-amine (27)**

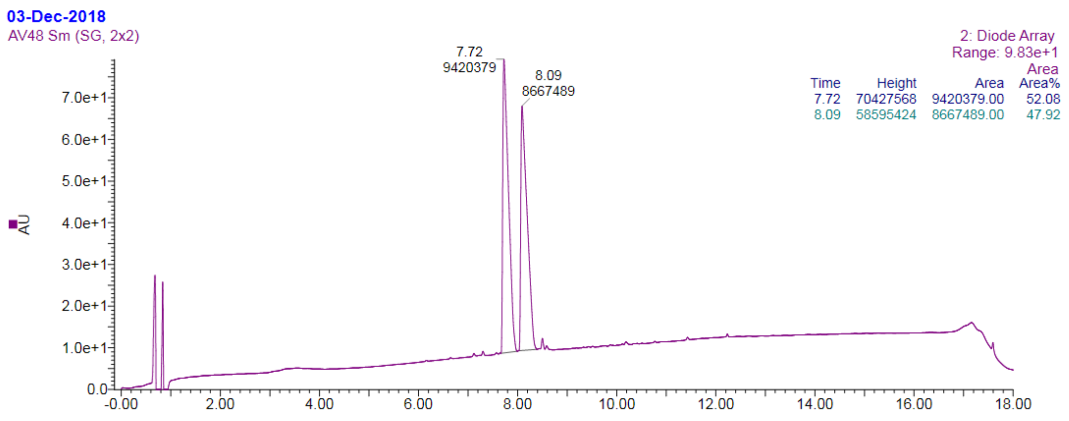
03-Dec-2018
AV48 Sm (SG, 2x2)
2: Diode Array
Range: 9.83e+1
7.72
9420379
8.09
8667489
Time Height Area Area%
7.72 70427568 9420379.00 52.08
8.09 58595424 8667489.00 47.92
AU

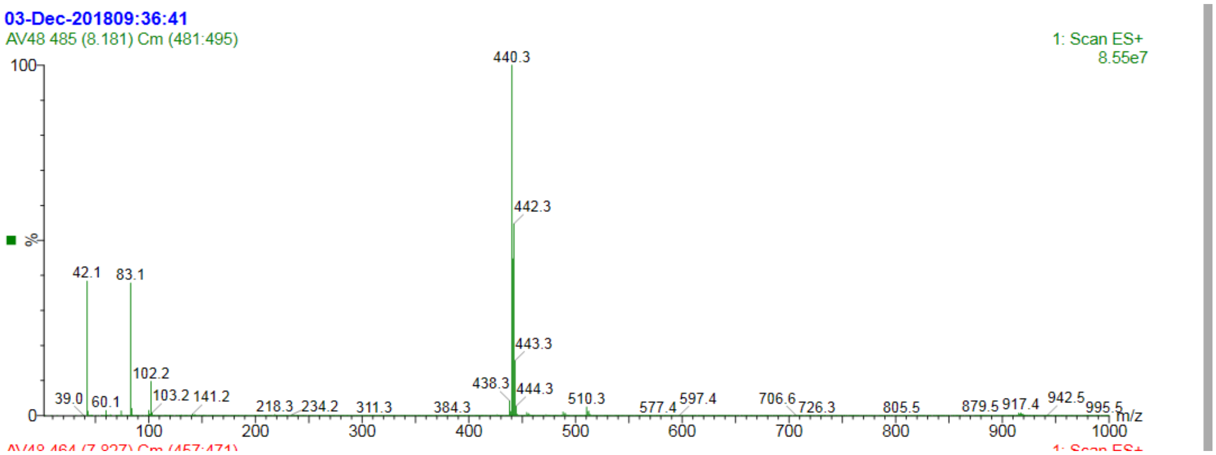
03-Dec-201809:36:41
AV48 485 (8.181) Cm (481:495)
1: Scan ES+
8.55e7
440.3
442.3
42.1
83.1
443.3
102.2
438.3
444.3
m/z

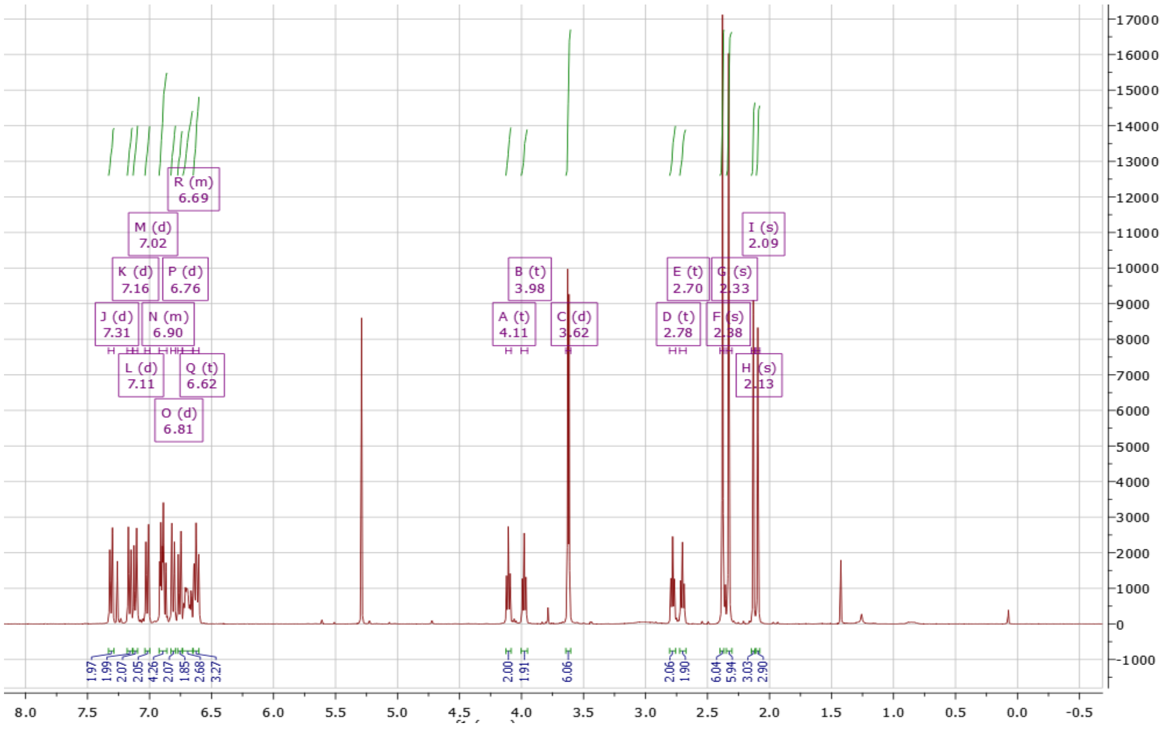

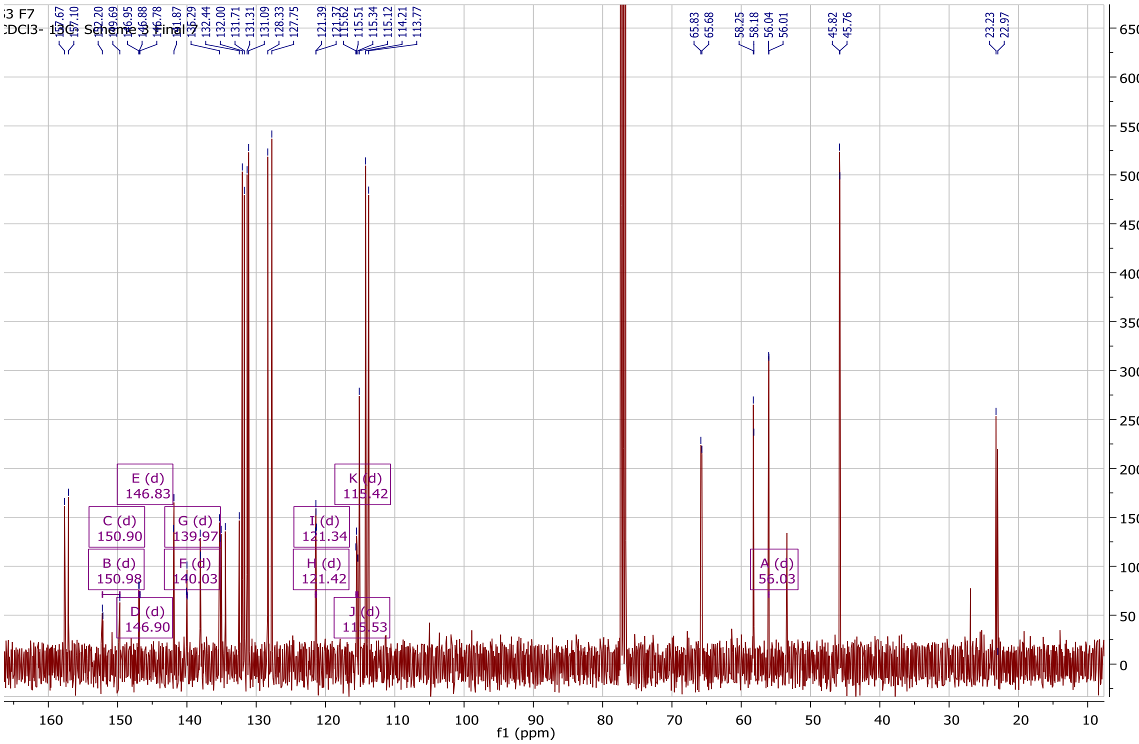

***E/Z*-(2-{4-[1-(4-Chlorophenyl)-2-(4-fluoro-3-methoxyphenyl)-propenyl]-phenoxy}-ethyl)-diethyl-amine (28).**

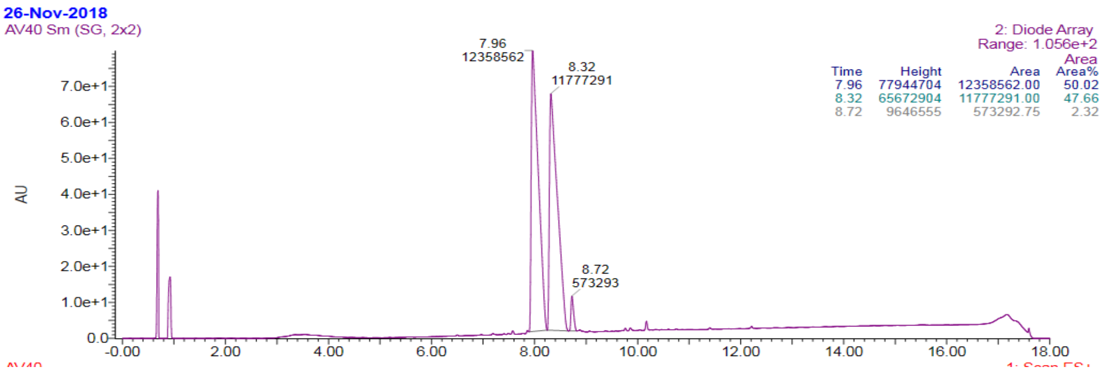
26-Nov-2018
AV40 Sm (SG, 2x2)
2: Diode Array
Range: 1.056e+2
7.96
12358562
8.32
11777291
8.72
573293
Time Height Area Area Area%
7.96 77944704 12358562.00 50.02
8.32 65672904 11777291.00 47.66
8.72 9646555 573292.75 2.32
AU
7.0e+1
6.0e+1
5.0e+1
4.0e+1
3.0e+1
2.0e+1
1.0e+1
0.0
-0.00
2.00
4.00
6.00
8.00
10.00
12.00
14.00
16.00
18.00

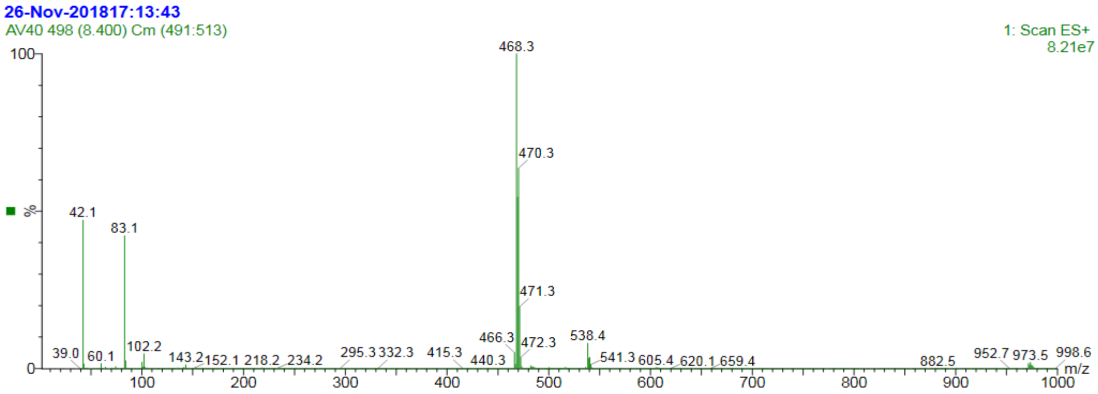
26-Nov-201817:13:43
AV40 498 (8.400) Cm (491:513)
1: Scan ES+
8.21e7
100
%
0
468.3
470.3
471.3
466.3
472.3
538.4
42.1
83.1
39.0
60.1
102.2
143.2
152.1
218.2
234.2
295.3
332.3
415.3
440.3
541.3
605.4
620.1
659.4
882.5
952.7
973.5
998.6
m/z
100
200
300
400
500
600
700
800
900
1000

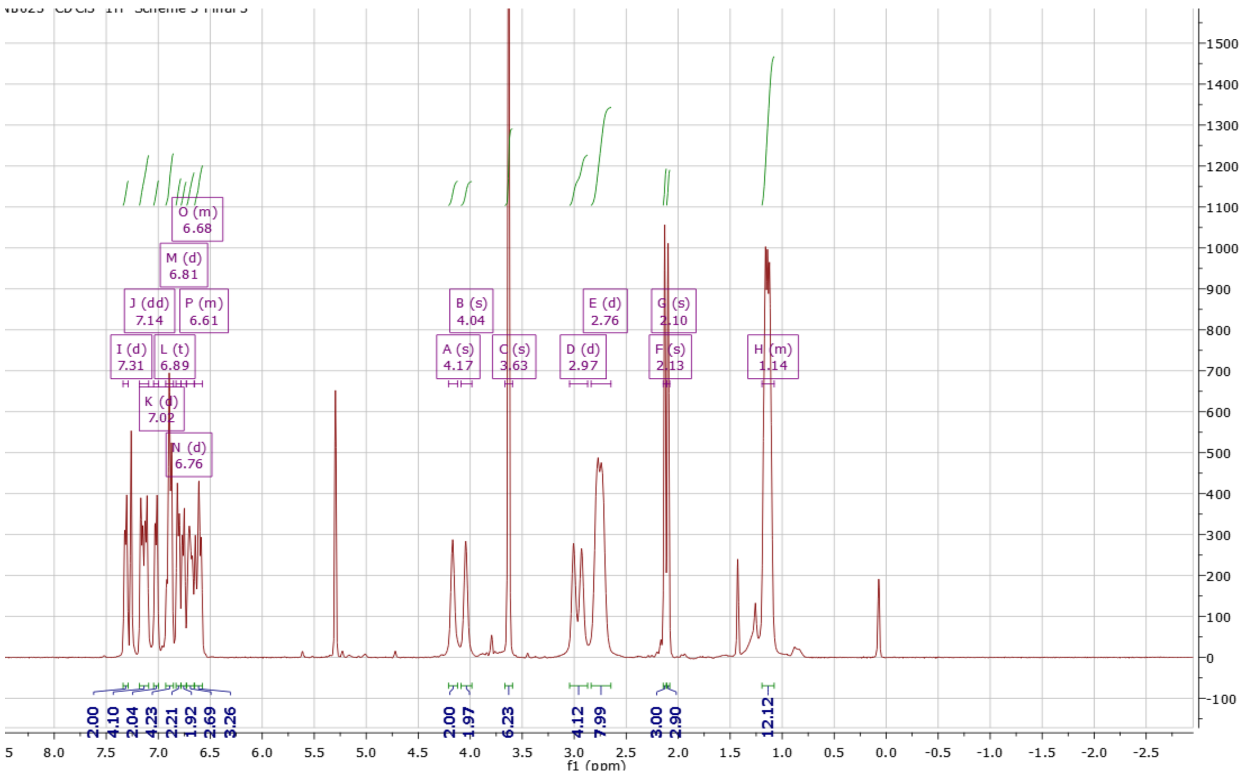
O (m) 6.68
M (d) 6.81
J (dd) 7.14
P (m) 6.61
I (d) 7.31
L (t) 6.89
K (d) 7.02
N (d) 6.76
B (s) 4.04
A (s) 4.17
C (s) 3.63
E (d) 2.76
D (d) 2.97
G (s) 2.10
F (s) 2.13
H (m) 1.14
2.00
4.10
2.04
4.23
2.21
1.92
2.69
3.26
2.00
1.97
6.23
4.12
7.99
3.00
2.90
12.12
f1 (ppm)

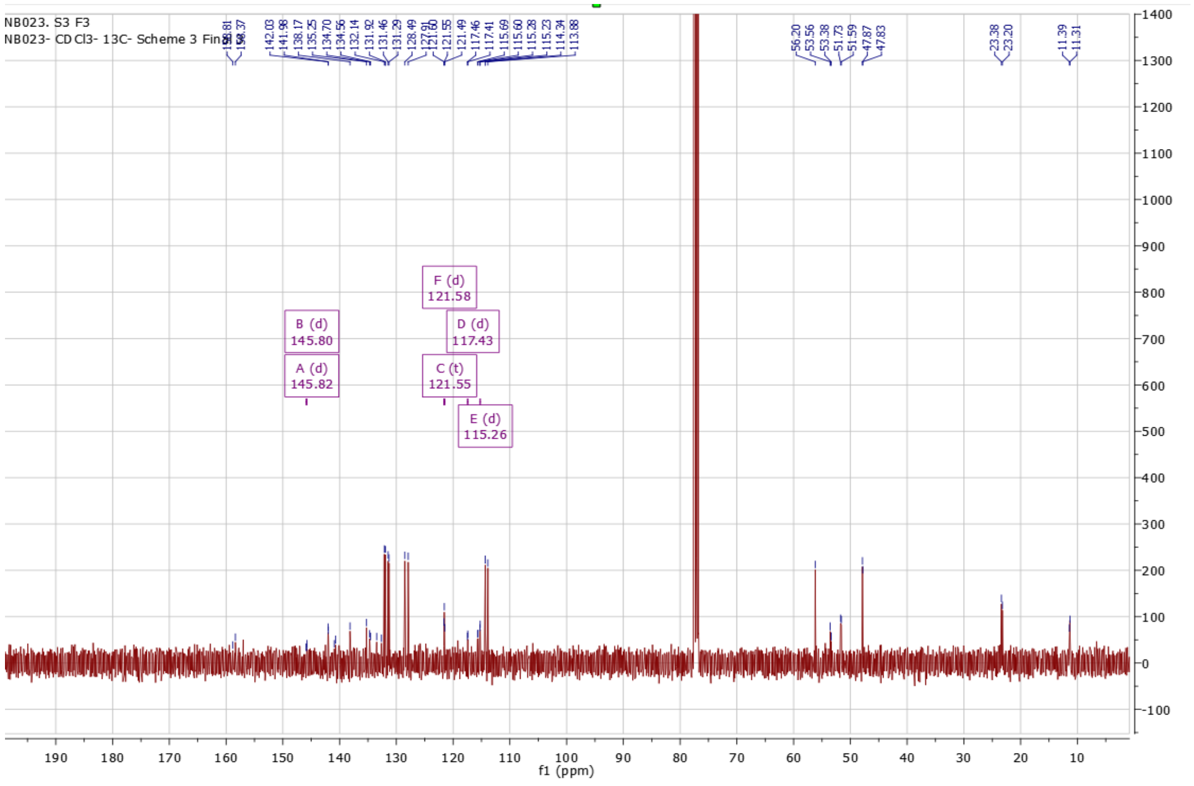
NB023. S3 F3
B (d) 145.80
A (d) 145.82
F (d) 121.58
D (d) 117.43
C (t) 121.55
E (d) 115.26
f1 (ppm)

2. Dose response curves for to determine EC_{50} values (agonistic activity) of selected compounds in YES assay, results of AlkP and uterotrophic assays

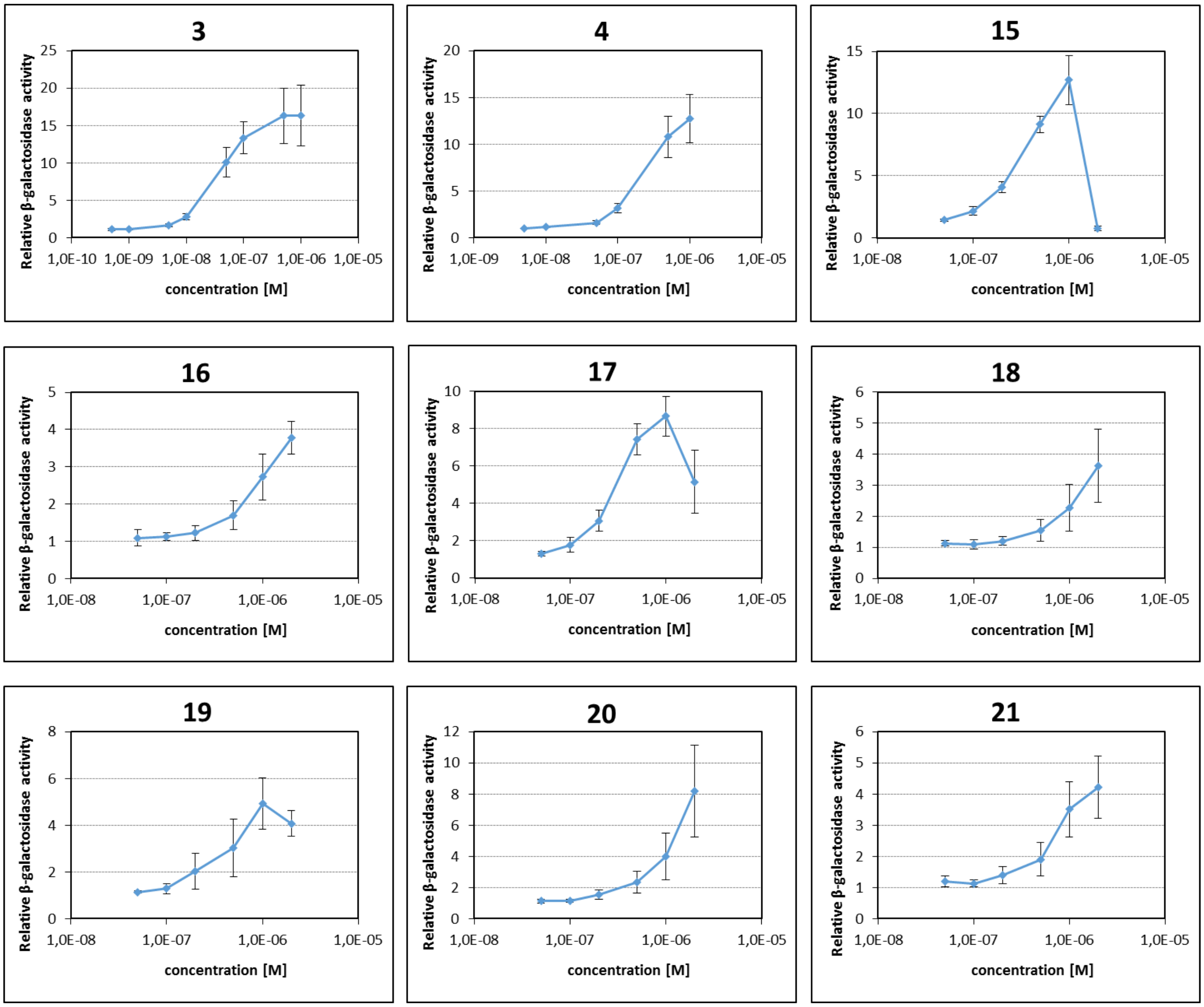

Figure S1: Relative ß-galactosidase activity of selected compounds after two days treatment in YES assay. DMSO was used as solvent control and set to 1, mean ± SD were calculated by three independent experiments.

Table S1: Relative Alkaline phosphatase activity (AlkP) after an incubation of 72 h in Ishikawa cells, compounds are tested at a concentration of 1 μM. DMSO was used as solvent control and set to 1, mean ± SD were calculated by three independent experiments.

**Code**	**Relative AlkP** activity<br>**Mean** ± **SD**
**3**	1.13 ± 0.43
**4**	1.10 ± 0.39
**5**	1.08 ± 0.33
**6**	2.55 ± 1.35
**7**	2.55 ± 1.80
**8**	1.16 ± 0.15
**9**	2.94 ± 1.29
**10**	2.55 ± 1.80
**11**	2.56 ± 0.83
**12**	1.43 ± 0.07
**13**	3.74 ± 2.05
**14**	6.58 ± 2.24
**15**	0.86 ± 0.12
**16**	0.98 ± 0.53
**17**	1.31 ± 0.41
**18**	1.01 ± 0.24
**19**	1.71 ± 0.08
**20**	1.12 ± 0.46
**21**	0.90 ± 0.13
**22-28**	Compounds were not selected for AlkP assay because of their low estrogenic effects in YES assay

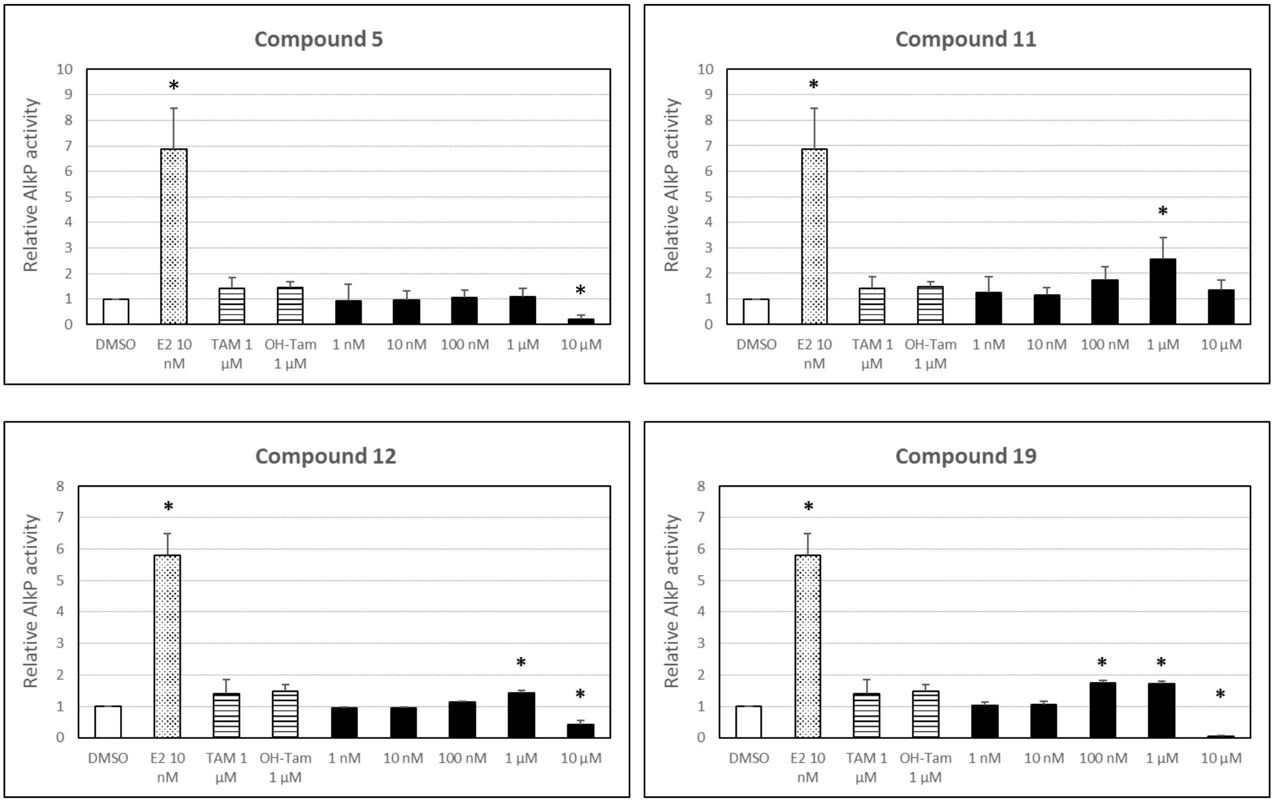

Figure S2: Relative Alkaline phosphatase activity (AlkP) for compounds **5, 11, 12, 19** after three days treatment in human endometrial adenocarcinoma Ishikawa cells. DMSO was used as solvent control and set to 1, mean ± SD were calculated by three independent experiments. Tam and 4-OH-Tam (1 µM) were tested for comparison. * $p < 0.05$ versus DMSO control (Tukey test)

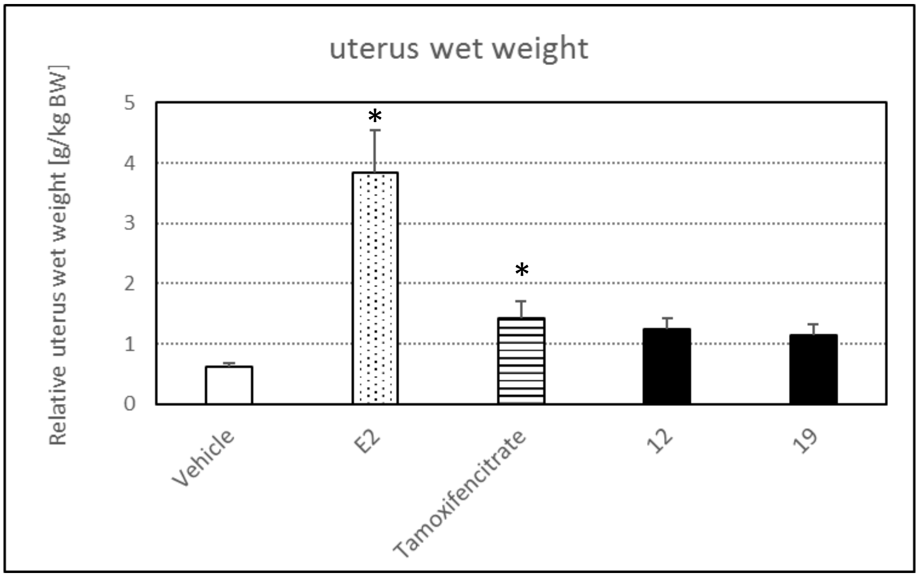

Figure S3: Relative uterus wet weight following a three days treatment of ovariectomized rats (vehicle = control) with E2 (10 µg/kg BW/ day), TAM (10 mg/kg BW/ day), compound **12** (10 mg/kg BW/ day), and compound **19** (10 mg/kg BW/ day). * $p < 0.05$ versus control (Tukey test)

Figure S4: DTP log (GI_{50}, TGI, LC_{50}) μM TAM (NSC 180973)

GI_{50} Mean Graph for Compound 180973

NCI Cancer Screen Current Data

Average GI_{50} over all cell lines is 3.94E-6 M

Cell Panel	Cell Line	Log GI_{50}
Leukemia	CCRF-CEM	-5.5
	HL-60(TB)	-5.6
	K-562	-5.7
	MOLT-4	-5.6
	RPMI-8226	-5.6
	SR	-5.9
Non-Small Cell Lung	A549/ATCC	-5.4
	EKVX	-5.2
	HOP-62	-5.0
	HOP-92	-5.6
	NCI-H226	-5.0
	NCI-H23	-5.3
	NCI-H322M	-5.1
	NCI-H460	-5.5
	NCI-H522	-5.2
Colon	COLO 205	-5.6
	HCC-2998	-5.5
	HCT-116	-5.4
	HCT-15	-5.5
	HT29	-5.6
	KM12	-5.5
	SW-620	-5.5
Central Nervous System	SF-268	-5.2
	SF-295	-5.7
	SF-539	-5.3
	SNB-19	-5.2
	SNB-75	-5.3
	U251	-5.5
Melanoma	LOX IMVI	-5.6
	MALME-3M	-5.5
	M14	-5.6
	MDA-MB-435	-5.5
	SK-MEL-2	-5.3
	SK-MEL-28	-5.4
	SK-MEL-5	-5.6
	UACC-257	-5.5
	UACC-62	-5.2
Ovarian	IGROV1	-5.3
	OVCAR-3	-5.4
	OVCAR-4	-5.2
	OVCAR-5	-5.2
	OVCAR-8	-5.1
	NCI/ADR-RES	-5.3
	SK-OV-3	-5.0
Renal	786-0	-5.4
	A498	-5.2
	ACHN	-5.2
	CAKI-1	-5.4
	RXF 393	-5.6
	SN12C	-5.4
	TK-10	-5.4
	UO-31	-5.2
Prostate	PC-3	-5.5
	DU-145	-5.2
Breast	MCF7	-5.8
	MDA-MB-231/ATCC	-5.2
	HS 578T	-5.4
	BT-549	-5.2
	T-47D	-5.6
	MDA-MB-468	-5.7

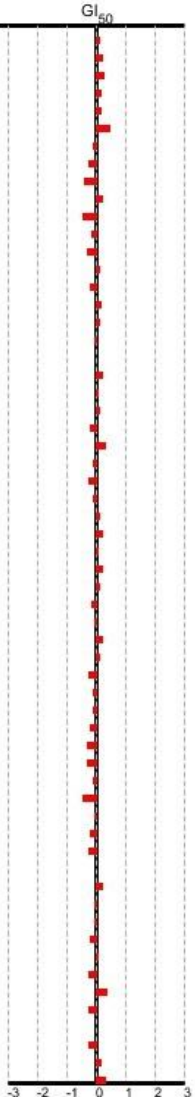

Figure S5: Dose response curves compound 11 on different subpanels (μM)

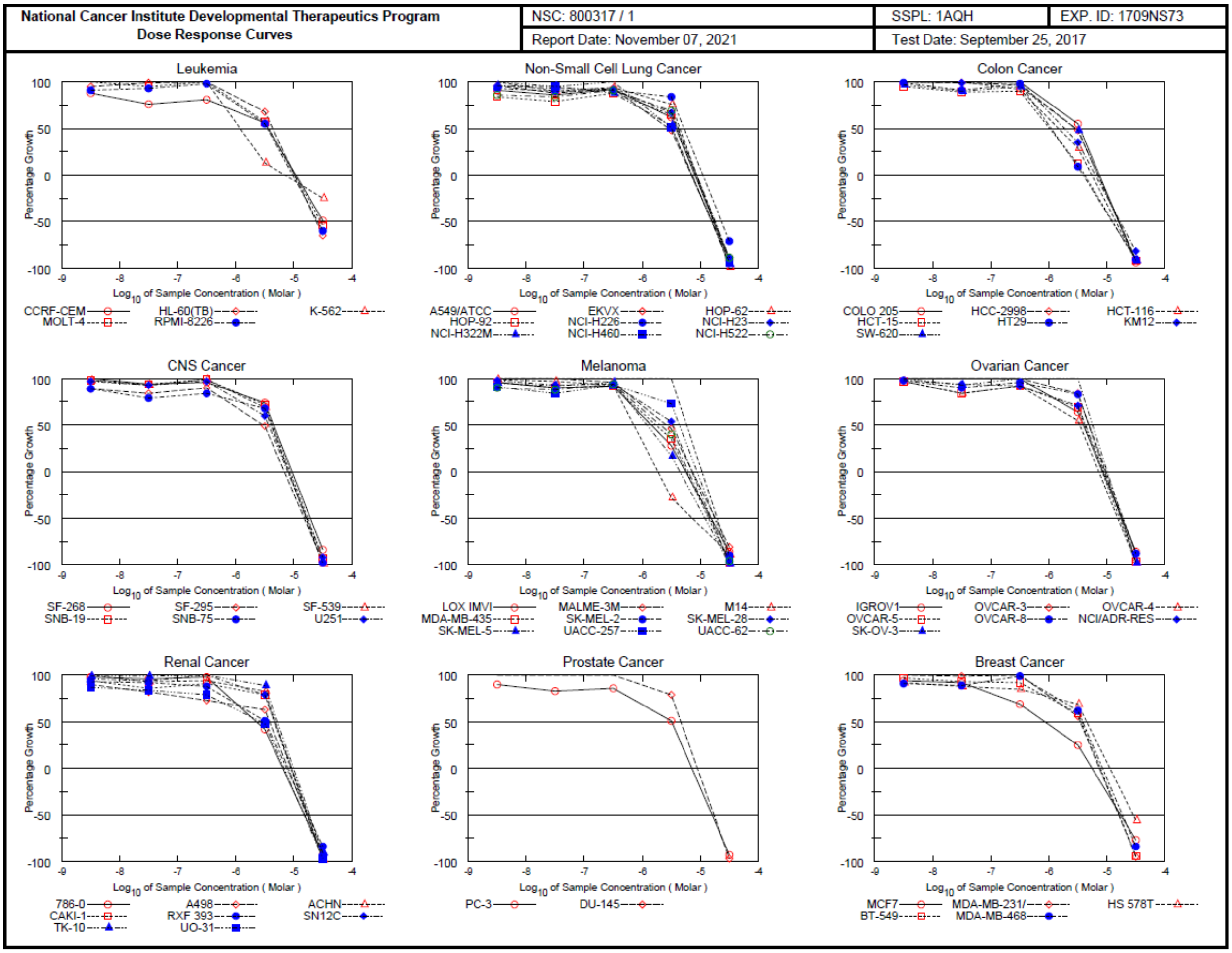

Figure S6: Dose response curves compound 12 on different subpanels (μM)

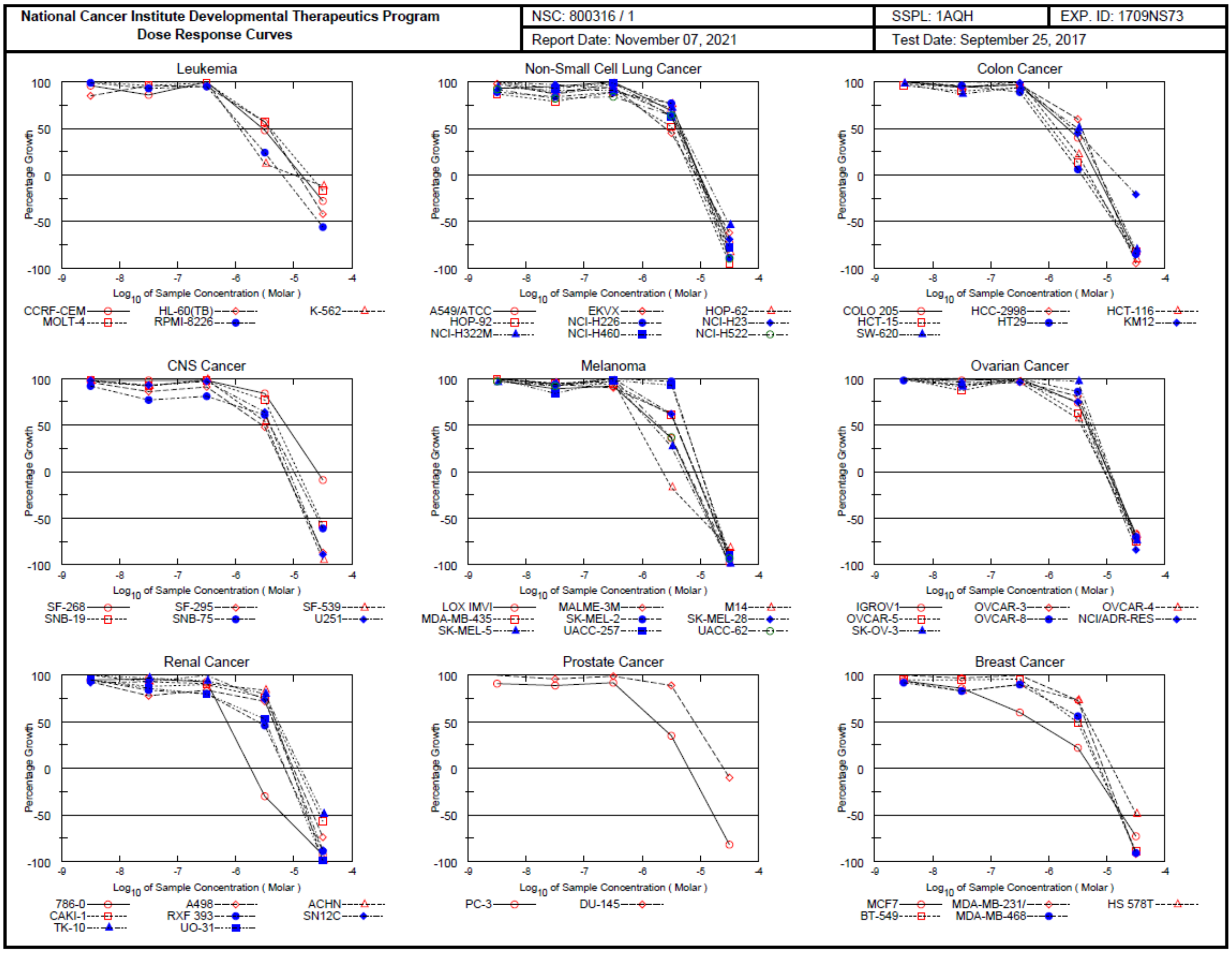

Figure S7: Dose response curves compound 16 on different subpanels (μM)

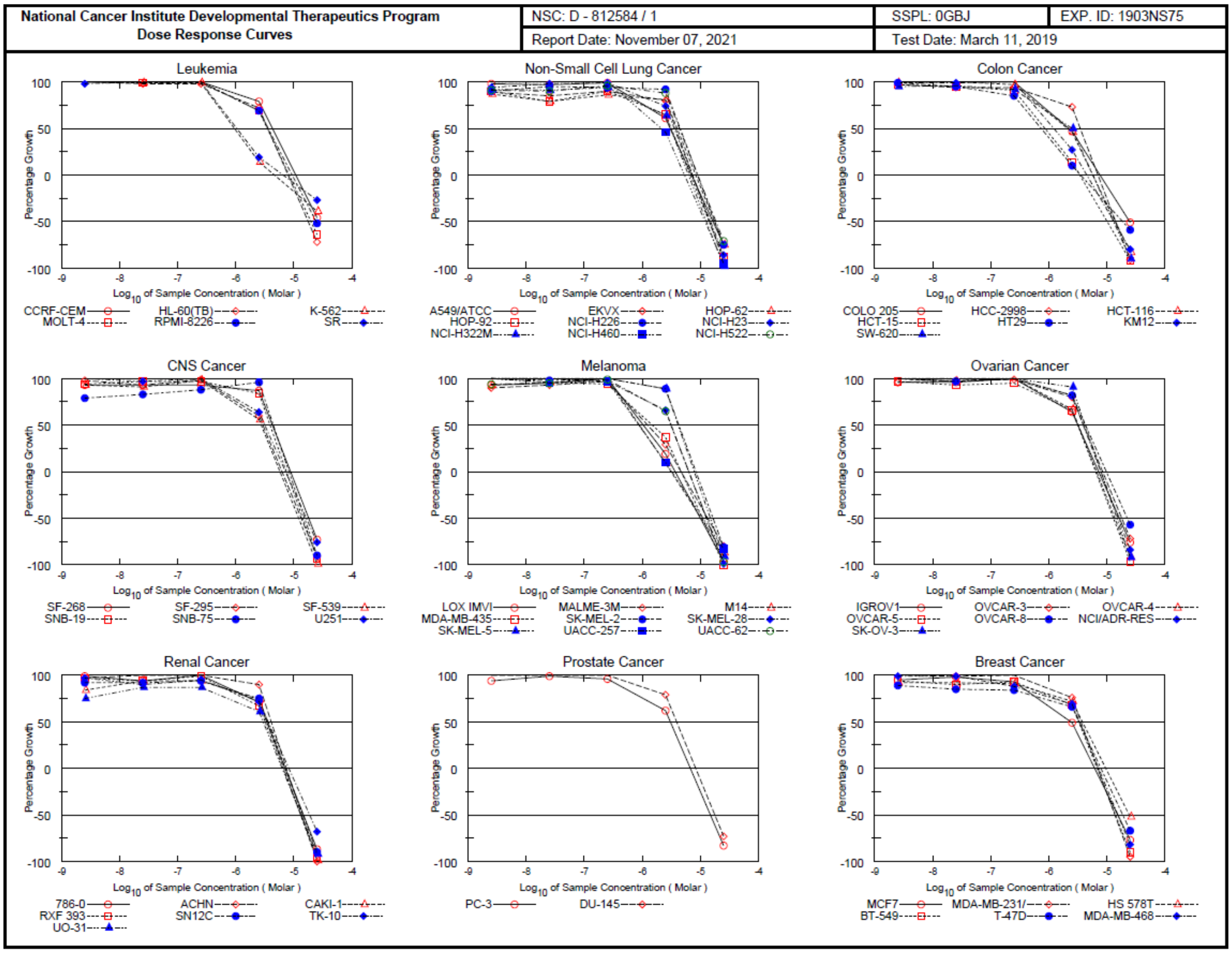

Figure S8: Dose response curves compound 17 on different subpanels (μM)

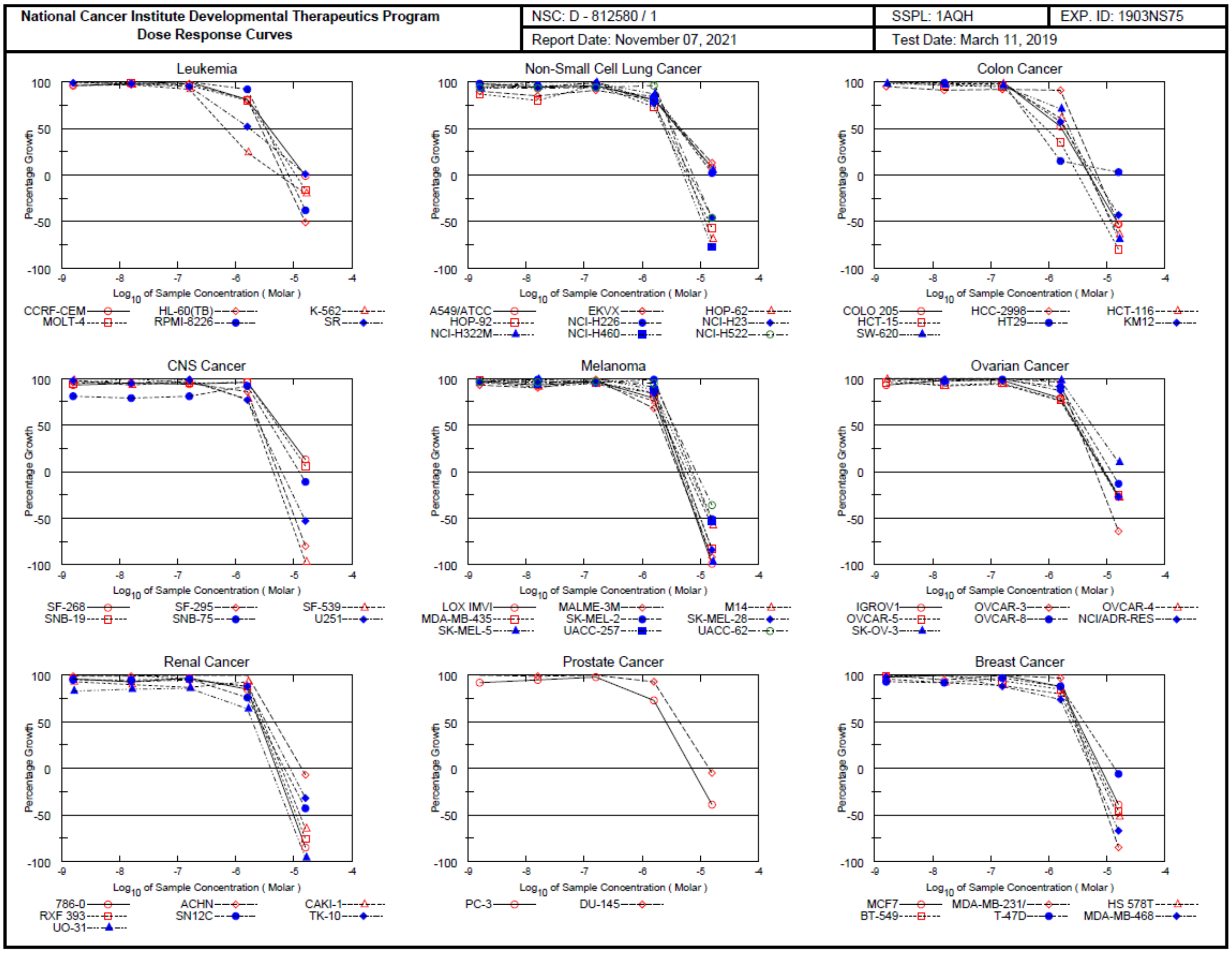

Figure S9: Dose response curves compound 19 on different subpanels (μM)

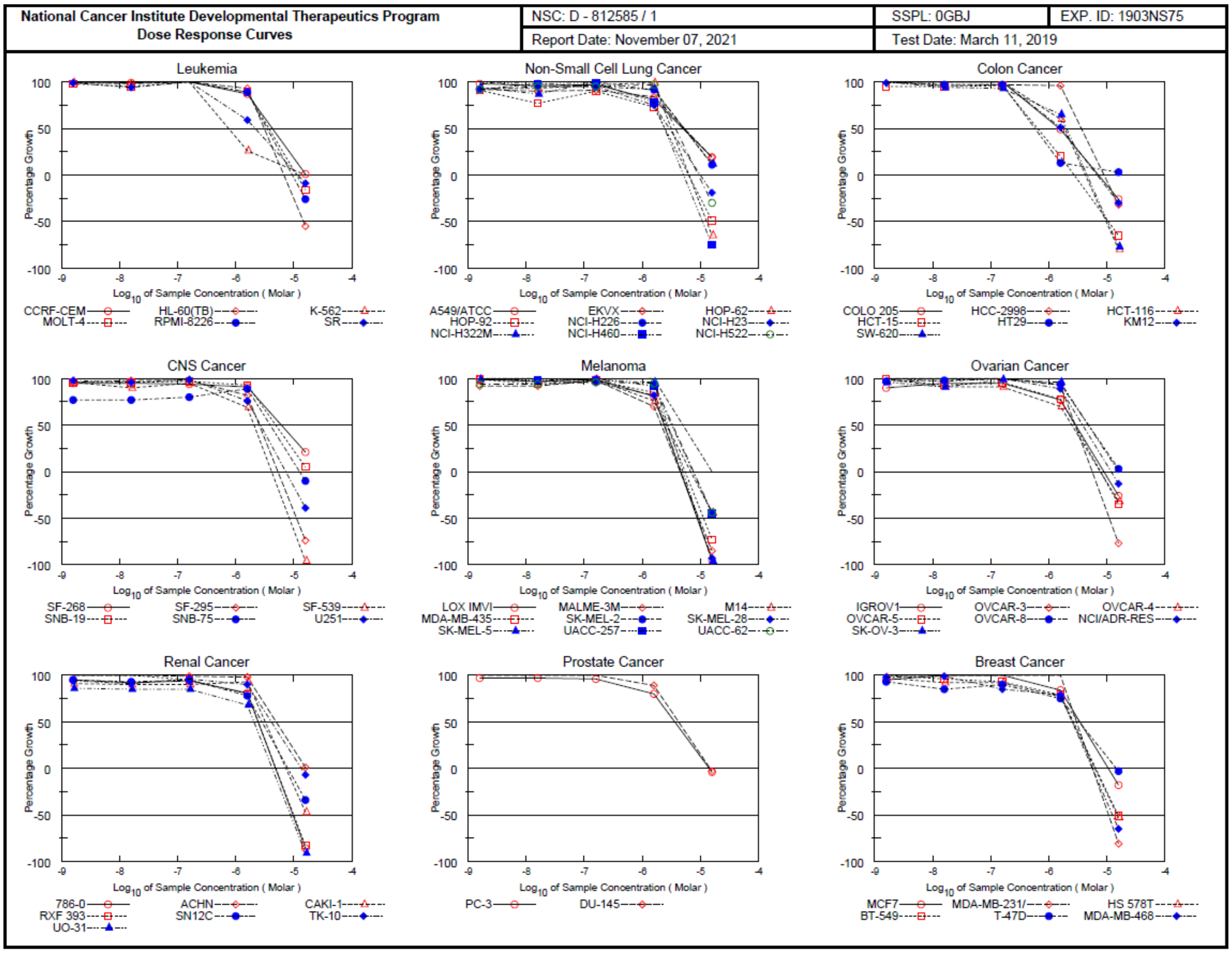

Figure S10: Dose response curves compound 19 on different subpanels (μM)

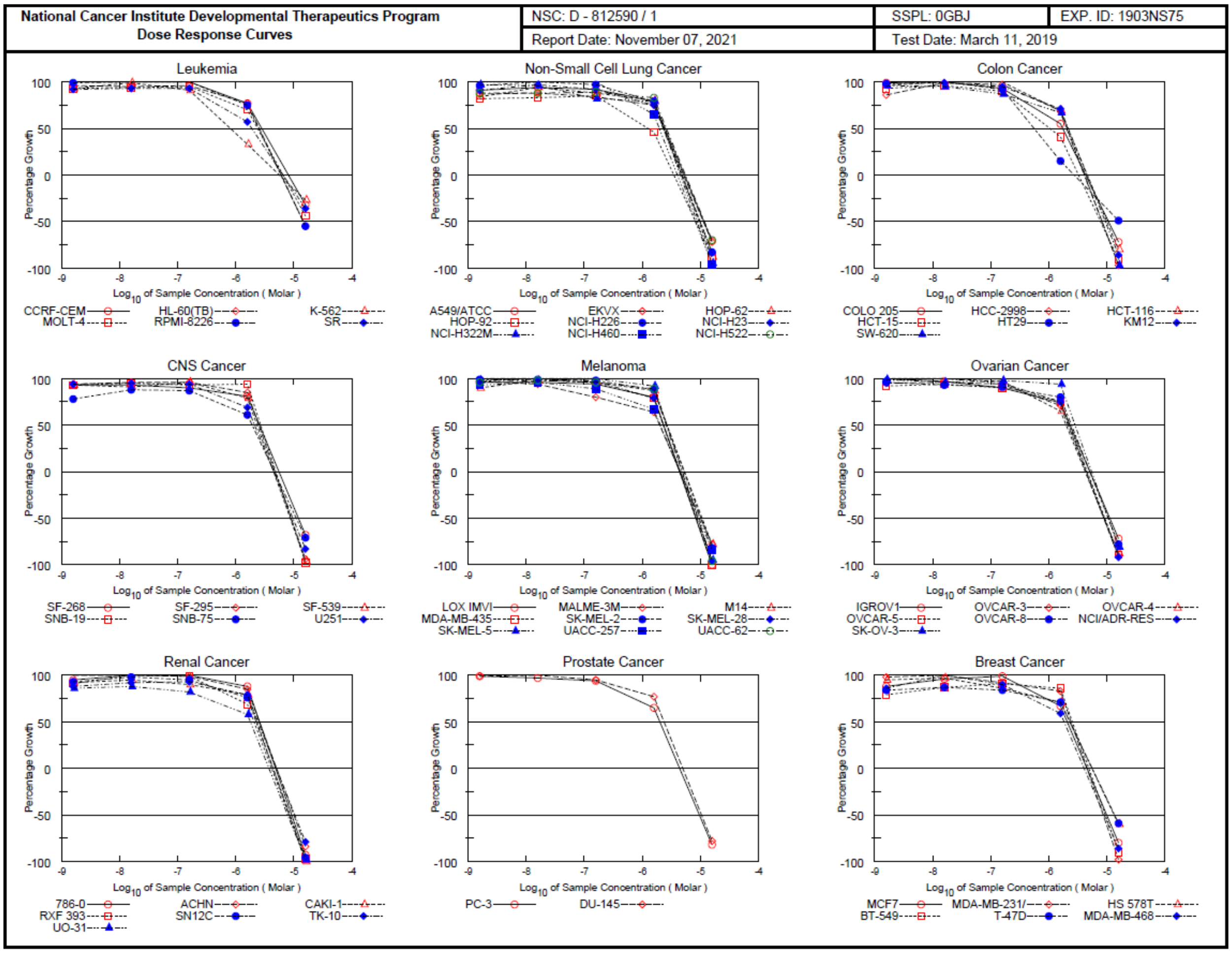

Supplement: Supplementary file 1 [file ijms-22-12575-s001.zip › ijms-1436095-supplementary.pdf]
